# “Neural Noise” in Auditory Responses in Young Autistic and Neurotypical Children

**DOI:** 10.1007/s10803-022-05797-4

**Published:** 2022-11-25

**Authors:** Patrick Dwyer, Svjetlana Vukusic, Zachary J. Williams, Clifford D. Saron, Susan M. Rivera

**Affiliations:** 1 Department of Psychology, UC Davis, Davis, CA USA; 2 Center for Mind and Brain, UC Davis, Davis, CA USA; 3 MIND Institute, UC Davis Health, Sacramento, CA USA; 4 Medical Scientist Training Program, Vanderbilt University School of Medicine, Nashville, TN USA; 5 Department of Hearing and Speech Sciences, Vanderbilt University Medical Center, Nashville, TN USA; 6 Vanderbilt Brain Institute, Vanderbilt University, Nashville, TN USA; 7 Frist Center for Autism and Innovation, Vanderbilt University, Nashville, TN USA; 8 Vanderbilt Kennedy Center, Vanderbilt University Medical Center, Nashville, TN USA; 9 College of Behavioral and Social Sciences, University of Maryland, College Park, MD USA

**Keywords:** Autism, Inter-trial variability, Electroencephalography, Inter-trial phase coherence (ITPC), Sensory processing, Loudness discomfort, Neural noise

## Abstract

Elevated “neural noise” has been advanced as an explanation of autism and autistic sensory experiences. However, functional neuroimaging measures of neural noise may be vulnerable to contamination by recording noise. This study explored variability of electrophysiological responses to tones of different intensities in 127 autistic and 79 typically-developing children aged 2–5 years old. A rigorous data processing pipeline, including advanced visualizations of different signal sources that were maximally independent across different time lags, was used to identify and eliminate putative recording noise. Inter-trial variability was measured using median absolute deviations (MADs) of EEG amplitudes across trials and inter-trial phase coherence (ITPC). ITPC was elevated in autism in the 50 and 60 dB intensity conditions, suggesting diminished (rather than elevated) neural noise in autism, although reduced ITPC to soft 50 dB sounds was associated with increased loudness discomfort. Autistic and non-autistic participants did not differ in MADs, and indeed, the vast majority of the statistical tests examined in this study yielded no significant effects. These results appear inconsistent with the neural noise account.

**Supplementary Information:**

The online version contains supplementary material available at 10.1007/s10803-022-05797-4.

## Introduction

Many autistic people’s^1^ accounts (e.g., Grandin, 1992; Willey, 1999; Williams, 1992) have long emphasized the importance of sensory experiences in Autism Spectrum Development (ASD; see also O’Neill & Jones, 1997). These attempts to emphasize the importance of sensory processing were initially neglected (Grandin & Panek, 2014); for some time, limited autism sensory research was conducted (O’Neill & Jones, 1997), and in this period, neurocognitive accounts of autism focused on social-communication characteristics (Rogers & Ozonoff, 2005). Fortunately, this has changed: a glance at a graph depicting the annual number of research articles addressing autistic sensory processing suggests that the output of research on the topic has been accelerating since approximately 2005 (Ben-Sasson et al., 2019). These new studies have provided ample evidence to highlight the importance of sensory processing to autistic people’s lived experiences and well-being. For example, studies suggest that sensory sensitivities in ASD are associated with, or even an aspect of, quality of life (Lin & Huang, 2019; McConachie et al., 2019). Autistic sensory experiences and behaviours are related to participation in everyday activities (Ismael et al., 2018; Little et al., 2015). Selective eating in autism is associated with sensory discomfort as well as behavioural “rigidity” (Zickgraf et al., 2020), and dietary patterns in autism are in turn related to microbiota composition and gastrointestinal symptoms (Berding & Donovan, 2018; Yap et al., 2021). Sensory processing in autism is also related to sleep quality (Tzischinsky et al., 2018) and longitudinal research shows that differences in sensory behaviour predict later anxiety outcomes in autism (Green et al., 2012; see also Williams et al., 2021a).

Furthermore, beyond the importance of sensory processing in autistic people’s experiences, participation in the world, and well-being, there is reason to believe that early differences in how autistic people attend to and process sensory stimuli in their environments could have important influences on non-sensory autism characteristics. Altered autistic sensory processing can be observed behaviourally and neurophysiologically as early as infancy (Baranek, 1999; Kolesnik et al., 2019) and repetition suppression to sensory stimuli in infants at elevated likelihood of ASD has been found to be associated with later autistic traits (Piccardi et al., 2021). Early differences in sensory processing patterns predict later social and language outcomes in ASD (Baranek et al., 2018; Damiano-Goodwin et al., 2018; Grzadzinski et al., 2020; Kolesnik et al., 2019). Indeed, even in autistic older individuals, distracting tactile sensory inputs can cause autistic participants to demonstrate atypical patterns of brain activity in social tasks (Green et al., 2018).

### Intra-Individual Noise Variability

One neurobiological-level explanation that has been offered for altered sensory experiences in autism is the theory autism is characterized by high levels of endogenous, intra-individual variability or “neural noise” (Haigh, 2018; Ward, 2018). According to this account, brain responses to sensory stimulation in ASD are unstable and unreliable, such that autistic individuals cannot easily extract information from their environments. Such intra-individual neural noise might not only make it more difficult for autistic people to understand complex social situations but could also contribute towards experiences of sensory distress and overload.

If autistic people do have more unstable sensory responses than typically-developing individuals, this difference might reflect an atypical balance of excitation and inhibition, which has been advanced as an organizing framework for understanding autism at the neural level (Rubenstein & Merzenich, 2003; Sohal & Rubenstein, 2019). If the ratio of inhibition to excitation is reduced in ASD, even in specific circuits along dynamic timescales, inhibitory processes might fail to regulate neural sensory processing well enough to contribute to a stable experience of the world. Such differences in excitation:inhibition ratios could reflect concentrations of neurotransmitters such as GABA and glutamate, and some recent magnetic resonance spectroscopy studies examining levels of these neurotransmitters have found ASD-Typical Development (TD) differences consistent with the excitation-inhibition imbalance account (Puts et al., 2017; Sapey-Triomphe et al., 2019); studies of non-human animal models of a number of different genetic variants associated with autism have often yielded similar results (reviewed by Bozzi et al., 2018; Castro & Monteiro, 2022; Sierra-Arregui et al., 2020). GABAergic neurotransmission appears particularly atypical in some animal models of monogenetic variants associated with autism, as well as in animals exposed to valproate (Bozzi et al., 2018; Castro & Monteiro, 2022; Sierra-Arregui et al., 2020), although it is unclear whether these findings might generalize to polygenetic and idiopathic autism. Indeed, some human studies have failed to observe ASD-TD group differences in GABA or glutamate (Kolodny et al., 2020; Umesawa et al., 2020). Some studies even report results that are seemingly contrary to the predictions of the excitation-inhibition account of autism, such as reduced glutamate and glutamine (Edmondson et al., 2020) or increased GABA (Fung et al., 2020; Maier et al., 2022) in autistic participants (see also Dickinson et al., 2016 for a critical review of studies in this area). One study suggests apparent reductions in GABAergic neurons in some mouse models may actually have reflected altered protein function, potentially resulting in enhanced inhibition to excitation, instead of vice versa (Filice et al., 2016).

Functional neuroscience research in humans does not clarify the true level of intra-individual variability at the neural level in ASD. Various neuroscience studies offer evidence both for (e.g., Dinstein et al., 2012; Latinus et al., 2019; Milne, 2011) and against (e.g., Butler et al., 2017; Randeniya et al., 2022) the idea of increased intra-individual variability of sensory responses in autism. Some authors even argue that intra-individual noise is *reduced*, not elevated, in autism (Davis & Plaisted-Grant, 2015). One fundamental challenge dogging empirical research on intra-individual neural variability may be the danger of non-neural, artefactual sources of noise. While data processing techniques can be used to remove putatively non-neural artefacts, there appears to be no way of absolutely guaranteeing that remaining, putatively neural data are definitely neural in origin. This appears potentially problematic, given that neuroscience data quality in ASD samples can be poorer than in TD samples (DiStefano et al., 2019; Yerys et al., 2009), and it emphasizes the importance of rigorous data collection and processing procedures.

The intra-individual noise theory must also explain phenomena with which it appears somewhat inconsistent. Autistic individuals often exhibit enhanced or at least unimpaired sensory performance on locally-oriented behavioural tasks (see, e.g., Mottron et al., 2006; Van der Hallen et al., 2015), and if not superior then at least unimpaired sensory acuity (e.g., Albrecht et al., 2014; Bölte et al., 2012; Tavassoli et al., 2011), whereas neural noise would seem to predict poor performance. It has been suggested that this might reflect stochastic resonance, such that an increase in noise to an optimal level might counterintuitively enhance autistic performance (Simmons et al., 2009). However, in ASD, recent research indicates that better visual search performance is related to increased rather than diminished levels of GABA (Edmondson et al., 2020), which appears to conflict with a stochastic resonance-based argument.^2^ Furthermore, it is noteworthy that autistic people’s qualitative accounts of their sensory experiences often mention how experiences are affected by contextual factors such as one’s prior internal emotional states or one’s degree of control over stimuli (McLennan et al., 2021; Robertson & Simmons, 2015; Smith & Sharp, 2013), or over development (Kirby et al., 2015); these would be examples of regular, somewhat predictable variations, not random ones.

Of course, these subjective experiences need not accurately reflect underlying neural mechanisms. However, important questions exist regarding the neural consequences of atypical balances of excitation and inhibition. Although proponents of the excitation-inhibition account focus on how increases in noise (i.e., background activity as well as neural responses to inputs that are not “behaviourally meaningful”) would lead to an overall diminution of signal-to-ratio (Rubenstein & Merzenich, 2003; Sohal & Rubenstein, 2019), the account does suggest that neural responses to sensory signals are also increased in autism (Sohal & Rubenstein, 2019). The account’s proponents believe this increase in signal would be outweighed by the increase in noise, but one could theoretically argue otherwise. Indeed, it may be important to interrogate the meaning of the term “noise.” For example, some background sensory stimuli might be regarded as “noise” if they are considered to be behaviourally-irrelevant by most typically-developing people, but they would still be external stimuli, and in some sense, would appear to represent a “signal.” Some positive aspects of autistic sensory interests and hyper-focus could potentially be seen in this light and might be regarded as behaviourally-meaningful by many autistic people (see, e.g., Jones et al., 2003; Smith & Sharp, 2013).

Thus, there may be reason to believe that balances of signal and noise in autism could be complex and context-dependent. This conclusion might also follow if one were to (at least for the sake of argument) accept the premise that stochastic resonance can explain enhanced local perception in autism. If inter-trial variability of sensory responses might be elevated in autism in some contexts but not others, this could potentially help account for inconsistencies in prior literature. As noted by Butler et al. (2017), increased inter-trial latency jitter of event-related responses should lead to changes in the morphology of ERPs, such as attenuation or broadening (see also Luck, 2014a, pp. 267–271). While amplitudes of many auditory ERPs appear similar in ASD and TD, others do not (reviewed by Williams et al., 2021b). The canonical auditory ERPs evident in children from the age range of the present study include the P1 response, a large positive voltage deflection occurring approximately ~ 100–150 ms after stimulus onset, and the frontocentral N2, a negative voltage deflection occurring approximately ~ 250 ms after stimulus onset (Čeponiene et al., 2003; Ponton et al., 2002; Shafer et al., 2015). These ERP responses were previously described in the present study’s sample by Dwyer and colleagues (2021a): at the average level, the N2 response amplitude was attenuated in ASD, which is consistent with prior research (reviewed by Williams et al., 2021b). Insofar as this pattern of attenuated N2 responses in ASD could be consistent with increased autistic inter-trial variability, the present study may offer a particularly compelling opportunity to test the predictions of the neural noise account. In contrast, P1 response amplitudes do not appear to differ between ASD and TD (Williams et al., 2021b). Although the P1 can be enhanced by selective attention (Coch et al., 2005; Karns et al., 2015), it is considered a largely bottom-up as well as “obligatory” response (Donkers et al., 2015), and a clear P1 is more often observed in young children than an N2 (Dwyer et al., 2021b; Shafer et al., 2015). Thus, there seems to be relatively little reason to expect variability of the P1 to be elevated in ASD, perhaps unless heightened variability is often associated with autistic neural processing.

Furthermore, the present study includes stimuli of multiple intensities. Presentation of stimuli of multiple intensities may be advantageous in studies of “neural noise.” The phase consistency of EEG responses appears to increase with stimulus intensity (Schadow et al., 2007), suggesting that inter-trial variability is modulated by stimulus intensity. It is remains unclear whether ASD-TD group differences in “neural noise” would be intensity-dependent, e.g., due to any potential group differences in effectiveness of adaptation of “neural noise” levels to take advantage of the phenomenon of stochastic resonance, and therefore more easily observable at some intensities than others.

### Present Study

In the present study, inter-trial variability was examined using electrophysiology in a large sample of young autistic and typically-developing children from the Autism Phenome Project (APP) at the UC Davis MIND Institute. Complex tone stimuli of four different intensities were presented in a pseudo-randomly interspersed manner. While participants listened to these stimuli, they also watched a quiet video chosen specifically to be of interest to them personally. After data were collected, an intensive data processing pipeline that included second-order blind source identification (SOBI) independent components analysis (ICA; Belouchrani et al., 1997) was used to eliminate putatively artefactual noise sources, to minimize confounding inter-trial variability in neural responses with recording artefacts. In contrast to the theory of elevated “neural noise” in ASD, we believed it is possible that some prior findings of elevated “neural noise” in ASD might be at least partly influenced by recording artefacts, and that atypical balances of excitation-inhibition might enhance sensory “signal” as much or more than noise in some contexts or individuals. We therefore predicted that:

1. there would be no ASD-TD group differences in inter-trial variability of EEG responses to auditory stimuli; and
2. in autistic individuals, there would also be no associations between parent-reported loudness discomfort and inter-trial variability of EEG responses to auditory stimuli.

## Materials and Methods

### Participants

As part of the APP, attempts were made to collect ERP data from 216 autistic and 104 typically-developing children, aged between 2 and 5. These participants’ medical histories were screened for suspicion of hearing loss prior to their participation in the ERP portion of the APP. Autistic participants were required to meet criteria for a pervasive developmental disorder (based on DSM-IV and Collaborative Programs of Excellence in Autism Network criteria) and exceed ADOS-G (Lord et al., 2000) cut-off scores as well as cut-offs for either the social or communication subscales of the ADI-R (Lord et al., 1994) Further information about the APP and participant recruitment can be found in previous publications (e.g., Libero et al., 2016; Nordahl et al., 2011).

Technical problems with recordings, stimuli, etc., prevented collection of usable data from 8 participants (4 ASD, 4 TD). In 47 cases (40 ASD, 7 TD), recordings were not started or were terminated early due to participants resisting capping or moving excessively. 47 participants (37 ASD, 10 TD) provided data with an excessive number of bad channels (> 6–7), bridged channels, or artefacts. Five participants (3 ASD, 2 TD) either fell asleep or appeared sufficiently sleepy that their EEG data were considered likely to be seriously affected. 2 ASD participants were excluded due to neuroanatomical abnormalities revealed by magnetic resonance imaging collected in the APP. 5 participants (3 ASD, 2 TD) were excluded due to abrupt changes in global field power at the single trial level over the course of the experiment, plotted using ERPimage (Delorme et al., 2015), that were assumed to be recording-related rather than neural in origin and thus probable confounds in analyses at the single-trial level. Thus, a total of 89 autistic and 25 typically-developing participants were excluded after attempts were made to collect EEG data. Autistic participants were significantly less likely to provide usable data, χ^2^ = 8.29, *p* = 0.004, *OR* = 0.45 (CI_95%_ [0.27, 0.76]).

A further 11 participants were excluded, after attempts at EEG data collection, as ineligible for the study (10 excluded due to not meeting criteria for the autistic or typically-developing groups at the time-point reported here, 1 entered study in typically-developing group but diagnosed as autistic at a later time-point).

The final sample of children with usable data included in the present study compromised 79 typically-developing participants (27 female, 52 male) and 127 autistic participants (20 female, 107 male) (Table 1). Autistic participants were significantly less likely to be female, χ^2^ = 8.38, *p* = 0.004, *OR* = 0.36 (CI_95%_ [0.18, 0.70]). Families received a gift card in return for their participation in the study.

**Table 1:** Characteristics of typically-developing and autistic participants with usable electrophysiological data

	TD		ASD		*p*	Cliff’s δ [95% CI]
	Mean (SD)	Range	Mean (SD)	Range		
Chronological age (months)	37.06 (6.48)	25.80–56.33	38.49 (5.97)	25.50–54.87	.031	− .18 [− .33, − .02]
MSEL developmental quotient (DQ)	106.02 (11.50)	79.89–128.62	65.41 (20.54)	30.39–132.45	< .0001	.89 [.82, .94]
VABS-II adaptive behavior composite	110.63 (11.66)	82–135	75.44 (11.07)	53–104	< .0001	.96 [.27, > .99]
CBCL internalizing T-score	44.64 (9.92)	29–65	61.43 (9.40)	41–82	< .0001	− .77 [− .85, − .66]
CBCL externalizing T-score	47.41 (10.55)	28–71	58.88 (10.21)	28–85	< .0001	− .56 [− .68, − .41]
SPHI	− 0.45 (0.68)	− 1.45–1.11	0.04 (0.83)	− 1.45–2.16	.0002	− .34[− .49, − .16]
SSP tactile sensitivity (reverse-scored for consistency with SPHI)	5.27 (1.80)	4–14	8.31 (3.25)	4–18	< .0001	− .60 [− .72, − .45]
Total Trials	1166.75 (197.59)	711–1530	1142.14 (205.19)	639–1643	.55	.05 [− .11, .21]
Usable trials	935.95 (214.63)	448–1410	873.72 (193.33)	509–1375	.055	.16 [− .01, .32]
Rejected TRIALS	230.80 (103.29)	59–495	268.43 (101.93)	80–667	.014	− .20 [− .36, − .04]

Statistical comparisons employ Wilcoxon rank-sum tests. Cliff’s δ (Cliff, 1993) is reported as an effect size. Measures include the Mullen Scales of Early Learning (MSEL; Mullen, 1995) assessment, the Vineland Adaptive Behavior Scales (VABS)-II (Sparrow et al., 2005) which was administered as a parent-report questionnaire, the parent-report preschool-age form of the Childhood Behavior Checklist (CBCL; Achenbach & Rescorla, 2000), the Sensory Profile Hyperacusis Index (SPHI, described below), and raw scores from the parent-report SSP’s Tactile Sensitivity factor, from the factor solution described by Williams et al. (2018)

Note that SSP tactile sensitivity scores are reversed for consistency with SPHI, such that higher scores on both SPHI and the SSP tactile sensitivity factor reflect greater sensory sensitivity or discomfort

To examine whether participants excluded from the study based on bad EEG data differed from those participants who were included in the final sample, we compared their scores on a number of variables. These analyses are reported in the supplementary materials (Online Appendix A; Supplementary Figs. 1–6).

### Sensory Profile Hyperacusis Index (SPHI)

Loudness discomfort was measured with the Sensory Profile Hyperacusis Index (SPHI; Williams et al., 2020). This measure is derived from the Short Sensory Profile (SSP; McIntosh et al. 1999), a caregiver-report questionnaire commonly used in investigations of sensory processing in autistic children (Williams et al., 2018). Five SSP items reflecting auditory sensitivities and auditory filtering challenges (items 22, 24, 25, 34, and 35) are included in the SPHI. A bifactor item response theory model is used to estimate loudness discomfort scores based on the expected a posteriori (Bock & Mislevy, 1982) estimate of standing on the “general factor” underlying all five items. SPHI scores (on a Z-score scale) range from − 1.45 to 2.16, with higher scores reflecting greater loudness discomfort. Usable SPHI data were available from 106 autistic and 64 typically-developing participants.

### EEG Task

Participants were seated on a caregiver’s lap in a dimly-lit, audiometrically-quiet, shielded chamber and allowed to watch a quiet video of their choice or a video that their caregiver believed would be of interest to them. While they watched this video, Sony MDR-222KD binaural headphones calibrated with a B&K artificial ear (model 4153) and sound meter (model 2229) were used to passively present 50 ms (including 5 ms rise and decay time) complex tones (sine waves of equal amplitude overlaid at the following 7 frequencies (musical notes): 249 Hz (B3); 616 Hz (D5), 788 Hz (G5), 1042 Hz (C6), 1410 Hz (F6), 1952 Hz (B6), and 2749 Hz (F7)) presented at a randomly variable ISI of 1-2 s. Tones randomly varied in intensity (50 dB, 60 dB, 70 dB, and 80 dB SPL), and the presentation order was pseudorandomized such that multiple tones of the same intensity were never presented in succession. Presentation of tones was temporarily paused as required (i.e., when participants appeared to become restless). Approximately ~ 1100–1200 trials (~ 275–300 trials/condition) were collected from each participant (Table 1).

For comparison to the present study’s stimuli, background noise levels indoors in urban homes (i.e., with adjacent traffic) have been estimated at 48 dB A (Pearsons et al., 1977), while people two metres apart conversing in a moderately noisy environment such as a department store might speak at 61 dB A (Pearsons et al., 1977). 80 dB A corresponds to a freight train passing around thirty metres away (Occupational Safety and Health Administration, 2022) or a garburator (i.e., garbage disposal) operating one metre away (Canadian Centre for Occupational Health & Safety, 2019).

Further details regarding the EEG task are available in De Meo-Monteil et al. (2019).

### EEG Data Acquisition and Processing

EEG was collected with a 61-channel cap (www.easycap.de) and a Compumedics Neuroscan Synamp II amplifier sampling at a rate of 1000 Hz with Cz as a reference. EEG data were then filtered offline in BESA 5.2 (www.besa.de) with a low cut-off of 0.4 Hz (12 dB/octave roll-off). After low-cut filtering, the data were separated into epochs (spanning − 200 ms to 900 ms, including 300 ms necessary for subsequent independent components analysis), average-referenced, baseline-corrected (using the period from − 100 to 0 ms), and manually inspected for noisy channels, which were removed in preparation for later interpolation. The artifact scan tool of BESA 5.2 was then used to screen for and remove epochs with extreme amplitudes; amplitude thresholds were manually set for each participant to optimally balance removal of noise with retention of usable trials through creating a Raster plot of epochs sorted by extreme amplitude, then selecting specific epochs for examination of channel-by-channel time courses in order to find a subjective point from the Raster plot at which one could often select three epochs on one side and find them good, and three on the other and find them bad. All epochs were then manually inspected for abrupt voltage changes suggestive of temporary disconnection of electrode channels; epochs with such artefacts were removed. In the context of intra-individual variability, it is important to note, as shown in Table 1, that significantly more trials were rejected in the ASD group than the TD group, suggesting that recording quality was poorer in ASD.

Our calibration of artefact rejection thresholds to each participant’s data differs from some prior autism “neural noise” EEG studies, which may report simply using a single artefact rejection threshold for all participants (e.g., Butler et al., 2017; Randeniya et al., 2022).

Furthermore, to better address the potential confound of non-neural recording-related noise, we sought to remove putatively non-neural signal sources from the data, while taking care to preserve putatively neural signals. Therefore, the remaining epochs were submitted to a Second-Order Blind source Identification (SOBI; Belouchrani et al., 1997; Tang et al., 2005) independent components analysis (ICA). SOBI employs joint diagonalization of covariance matrices across different time delays, thereby taking into account temporal information from the EEG data, in order to separate the data into maximally uncorrelated “sources” with different spatial topographies and time courses. A semi-automatic artifact removal tool (SMART, Saggar et al., 2012, https://stanford.edu/~saggar/Software.html) was used to characterize the spatial topography, power spectra, autocorrelation, and time series of each SOBI source. These outputs were used to manually judge sources to be either putatively non-neural in origin (e.g., EMG, EOG, and blinks) or putatively neural. SOBI and SMART were applied separately to the first and second half of the data, consistent with recommendations for exploration of the effects of ICA on the data (Luck, 2014b).

The present study’s use of SOBI ICA presents a contrast with some prior EEG studies of “neural noise” in autism. Although use of ICA in autism “neural noise” studies is recommended in order to separate neural and non-neural signals (Butler et al., 2017), not all prior studies report using this technique (e.g., Butler et al., 2017; Randeniya et al., 2022). Moreover, studies using ICA may use techniques that do not consider temporal lags as SOBI does (e.g., Milne, 2011) or that are not fully described (e.g., Kovarski et al., 2019; Latinus et al., 2019). For example, it is unclear whether some prior studies may have relied on automatic detection of artefacts rather than on manual inspection of component timeseries, autocorrelation, power spectra, and topographies as in the present study. Notably, some studies may primarily use ICA to reject ocular artefacts (e.g., Kovarski et al., 2019; Latinus et al., 2019), whereas in the present study, SOBI was used to remove both ocular and muscular artefacts.

Data were subsequently reconstructed with putatively non-neural sources removed, and the artefact scan tool of BESA 5.2 was used once again to remove any remaining extreme amplitudes. Averages from each participant were computed and Cartool (Brunet et al., 2011) was used to screen the data for any channels that appeared to be systematically deviant from adjacent channels in the averaged data. Returning to the trial-by-trial data, these channels, as well as previously removed noisy channels, were interpolated using a spherical spline approach (Perrin et al., 1989) as implemented by Fieldtrip (Oostenveld et al., 2011) and baseline correction (using the 100 ms prior to stimulus onset) was repeated. Finally, separate trial epochs (now spanning 200 ms pre-stimulus onset to 599 ms post-stimulus onset) from each participant were filtered using ERPLAB (Lopez-Calderon & Luck, 2014). For median absolute deviation analyses, high-cut filters were second-order Butterworth with 40 Hz cutoff and 12 dB/octave roll/off. For inter-trial coherence analyses, Butterworth filters with 55 Hz cutoff were used. A Park-McClellan notch filter was applied at 60 Hz.

### Median Absolute Deviation (MAD) Analysis

For the first intra-individual variability analysis, the median absolute deviations^3^ of participants’ voltage amplitudes across all trials were extracted separately for each time-point between − 100 ms and 350 ms relative to stimulus onset, electrode, and intensity condition. That is, for a given participant, separately at each channel and in each condition, the median absolute deviation of single-trial EEG amplitudes 100 ms before stimulus onset was obtained, then the median absolute deviation (MAD) of single-trial EEG amplitudes 99 ms before stimulus onset, then the median absolute deviation of single-trial EEG amplitudes 98 ms before stimulus onset, and so on. 61 channels and 451 time-points were examined, yielding a total of 27,511 median absolute deviation values for each participant in each condition, representing the variability of that participant’s EEG responses across trials. Separately in each condition, median absolute deviations over a broad time window of 1–350 ms were then statistically compared across groups.

To correct for multiple comparisons involved in comparing these median absolute deviations across diagnostic groups, cluster-based permutation *t*-tests (Maris & Oostenveld, 2007) with 10,000 permutations were used. In this procedure, data points at which group differences exceed an initial significance threshold (corresponding in our study to a non-permuted two-tailed alpha of .05) are grouped together based on spatiotemporal adjacency, and the *t*-statistics for these spatiotemporally adjacent data points are summed to produce a cluster statistic. This cluster statistic can then be compared to a distribution of the largest cluster statistics obtained from permutations of the data; this permutation distribution essentially represents the largest cluster statistics that can be expected based on chance alone.

#### Correlation Analysis

Furthermore, in the autistic group, we explored ordinal Spearman’s ρ (rho) correlations between median absolute deviations and SPHI loudness discomfort scores using cluster-based permutation tests (Maris & Oostenveld, 2007) spanning every single channel and time point between 1 and 350 ms, separately in each intensity condition. If a spatiotemporally contiguous set of correlation *t*-statistics exceeded a parametric statistical significance threshold, the sum of their *t*-statistics was compared against a distribution of summed statistics obtained from randomly permuting the data 10,000 times.

#### Supplementary Analyses

We carried out a supplementary analysis comparing MADs of male and female participants in each diagnostic group using cluster-based permutation *t*-tests (Online Supplementary Materials; Appendix B).

We also carried out a cluster-based permutation analysis of correlations between MADs and SPHI loudness discomfort scores in the typically-developing group (Online Supplementary Materials; Appendix C).

### Inter-Trial Phase Coherence (ITPC) Analysis

Inter-trial phase coherence (ITPC), the degree to which instantaneous oscillatory phase in different frequencies and at different time points was consistent across trials, was examined separately in each intensity condition. ITPC values approaching 0 suggest a high degree of unreliability and inter-trial variability in responses to stimulus presentation, while ITPC values approaching 1 suggest low inter-trial variability. In this study, ITPC was examined in frequencies falling between 6 and 40 Hz, with 2 Hz steps between each frequency; Morlet wavelets with linearly increasing cycles from 1 cycle at 6 Hz to 3 cycles at 40 Hz (Delorme & Makeig, 2004) were used to extract ITPC values between − 107 and 506 ms. Separately in each condition, ITPC values between 1 and 350 ms were then entered into cluster-based permutation *t*-tests (Maris & Oostenveld, 2007) and compared across diagnostic groups. Data points at which group differences exceeded an initial significance threshold (corresponding in our study to a non-permuted two-tailed alpha of .05) were grouped based on spatial (electrode), temporal (time-point), and frequency contiguity. *T*-statistics from each contiguous group were then summed to produce cluster *t*-statistics, which were compared to a distribution of summed *t*-statistics generated from randomly permuting the data 10,000 times to determine whether the observed summed cluster *t*-statistics are larger than can be expected if the null hypothesis were true.

#### Correlation Analysis

Finally, in the autistic group, we explored ordinal Spearman’s ρ (rho) correlations between ITPC and SPHI loudness discomfort scores using cluster-based permutation tests (Maris & Oostenveld, 2007) spanning every single channel, all frequencies between 6 and 40 Hz, and all time points between 1 and 350 ms, separately in each intensity condition. If a set of contiguous correlation *t*-statistics exceeded a parametric statistical significance threshold, the sum of their *t*-statistics was compared against a distribution of summed statistics obtained from randomly permuting the data 10,000 times.

#### Supplementary Analyses

We carried out a supplementary analysis comparing ITPC of male and female participants in each diagnostic group using cluster-based permutation *t*-tests (Online Supplementary Materials; Appendix B).

We also carried out a cluster-based permutation analysis of correlations between ITPC and SPHI loudness discomfort scores in the typically-developing group (Online Supplementary Materials; Appendix C).

## Results

The mean of individual-level estimated SPHI reliability values was .85 in the ASD group and .83 in the TD group.

### Participants

Sample characteristics are presented in Table 1. Notably, given our analyses of correlations of EEG variability metrics with loudness discomfort as indexed by the SPHI, we observed significantly greater loudness discomfort in autistic than typically-developing participants.

However, as reported in Supplementary Materials (Online Appendix A), we observed few differences between participants who did or did not provide usable data. Autistic participants with lower cognitive ability scores were less likely to provide usable data, though this effect should be interpreted with caution as it might reflect general disinterest in complying with experimenter requests in both EEG and cognitive assessment contexts. Surprisingly, typically-developing participants with greater internalizing and externalizing symptoms nonsignificantly trended towards being more likely to provide usable EEG data.

### Median Absolute Deviations (MADs)

**Fig. 1:**
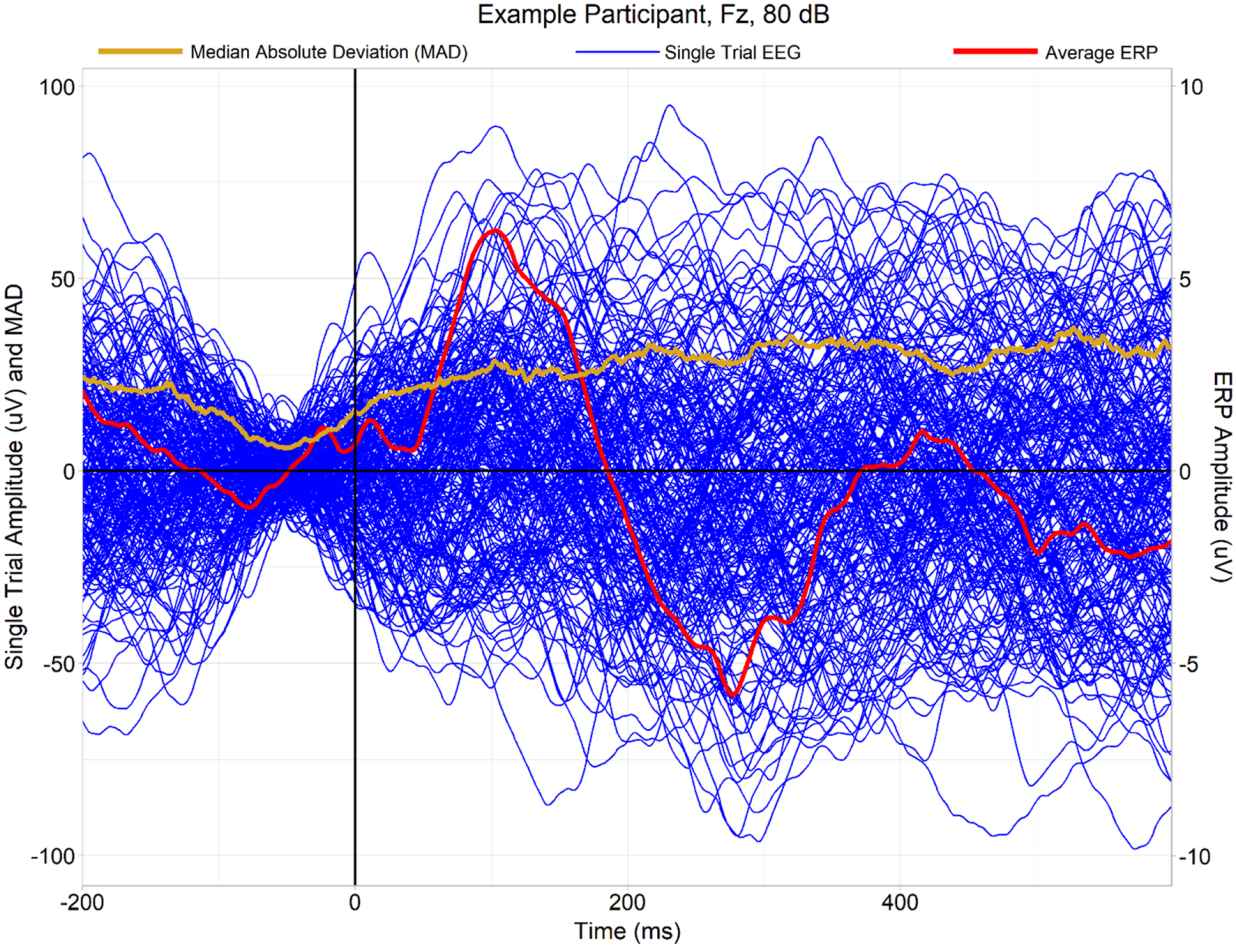
An example of a single participant’s trial-by-trial EEG waveforms observed at electrode Fz in the 80 dB condition (blue lines, superimposed above one another), as well as the averaged event-related potential derivable from the single trials (red line, scale exaggerated 10 × for ease of visual inspection). This participant exhibits clear P1 and N2 ERP peaks. Furthermore, the median absolute deviation (MAD) of the single trials at each time point is plotted (orange/gold line). The MAD diminishes during the baseline period, when variability of single trials is low due to baseline subtraction (which used the—100 ms to 0 ms interval), and it increases gradually thereafter. Unlike ITPC, which highlights event-related changes in phase synchrony (see Fig. 3), the MAD appears to more closely reflect the overall level of variability/“noise” present in the data

Median absolute deviations (MADs), indexing amplitude variability across trials, appeared smallest in the middle of the baseline period and they appeared to increase thereafter in each group (Fig. 1); this pattern is expected insofar as subtractive baseline correction of epochs should maximally reduce variability of trial amplitudes closest to the centre of the window used for baselining.

#### Group Comparisons

**Fig. 2:**
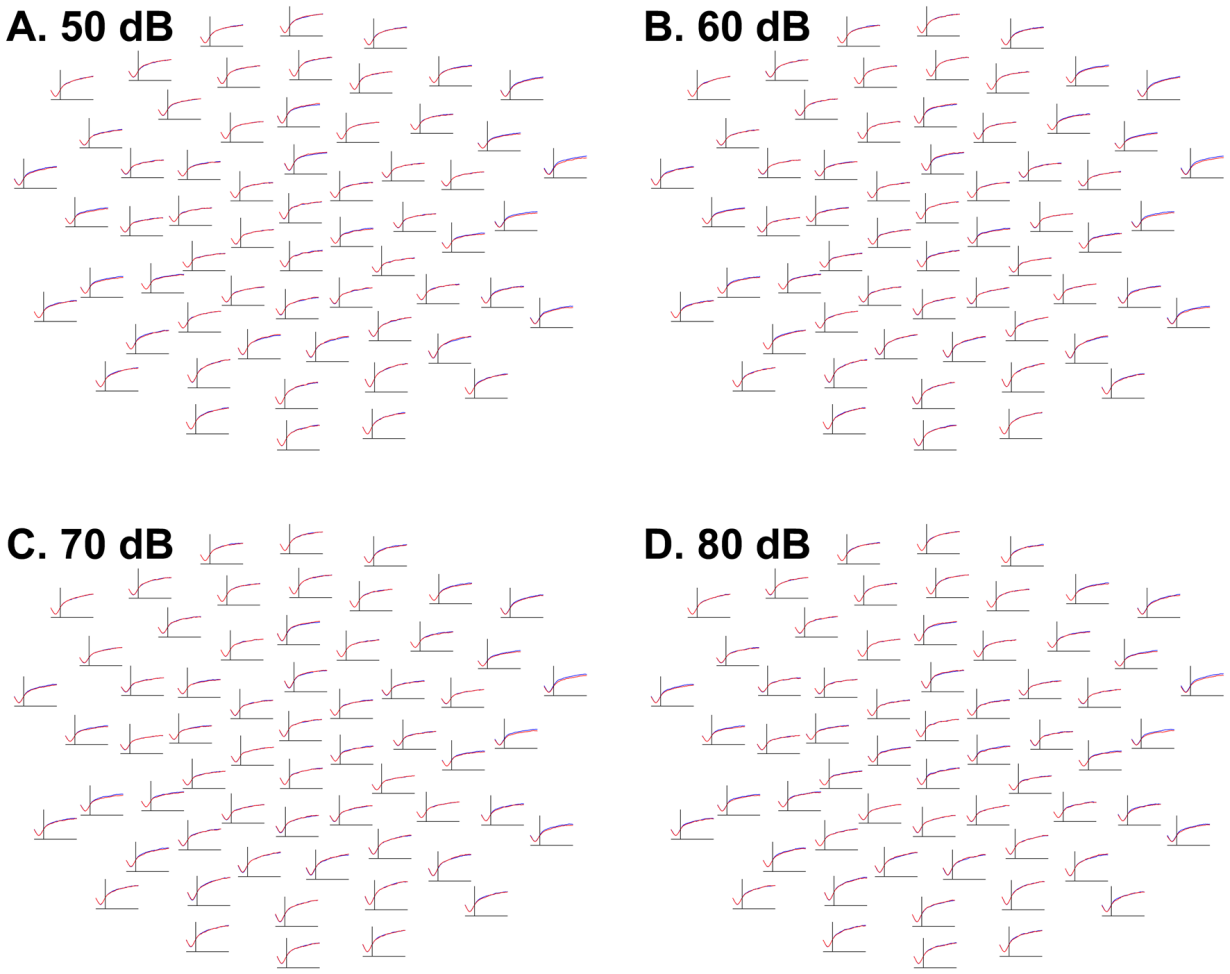
Waveforms at each electrode depicting, separately in each intensity condition and diagnostic group, the evolution of median absolute deviations of EEG amplitudes across trials, or the degree of inter-trial, intra-individual variability of EEG responses, between − 100 ms (left of each channel subplot) and 350 ms (right of each channel subplot). The Y-axis, representing median absolute deviation values, ranges from 2.5 (bottom of each channel subplot) to 25.0 (top of each channel subplot). Median absolute deviations averaged across autistic participants are blue; those averaged across typically-developing participants are red. Frontal channels are shown at the top and posterior channels at the bottom of each intensity condition panel. Although baseline correction caused variability to increase in each group farther away from the baseline, there were no significant differences between groups in any intensity condition. **A** (top left)*.* Waveforms depicting median absolute deviations of EEG amplitudes to 50 dB tones. **B** (top right). Waveforms depicting median absolute deviations of EEG amplitudes to 60 dB tones. **C** (bottom left). Waveforms depicting median absolute deviations of EEG amplitudes to 70 dB tones. **D** (bottom right). Waveforms depicting median absolute deviations of EEG amplitudes to 80 dB tones

There was no evidence that autistic and typically-developing participants differed in median absolute deviation values in any of the four intensity conditions (50 dB: lowest *p* = .46; 60 dB: lowest *p* = .46; 70 dB: lowest *p* = .26; 80 dB: lowest *p* = .30; Fig. 2).

As a failure to observe significant effects is not evidence of absence, we conducted a Bayesian analysis comparing MADs between groups (Online Supplementary Materials, Appendix D). Rather than using a mass multivariate cluster-based permutation approach, this supplementary analysis examined averaged MADs over regions of interest corresponding to the P1 and N2 ERPs, although given a lack of clear evidence of modulation of MADs by event-related responses such as ERPs (for example, see Fig. 1), this is better seen as a *post-hoc*, exploratory means of constraining multiple comparisons (channels*time-points) rather than as a means of exploring variability in the P1 and N2 ERPs themselves. The analysis found substantial evidence against the existence of meaningful group differences (defined by absolute values of Cohen’s *d* of at least 0.2) in MADs over these spatiotemporal regions.

#### Correlations

Moreover, in autistic participants, there was no evidence of significant correlations between loudness discomfort (i.e., SPHI) scores and median absolute deviations of EEG amplitudes across trials in the 50 dB condition (lowest *p* = .33), the 60 dB condition (lowest *p* = .40), the 70 dB condition (lowest *p* = .34), or the 80 dB condition (lowest *p* = .22). Figures depicting correlation coefficients at each time-point and channel are provided in Online Supplementary Materials, Appendix E (Supplementary Figs. 22–25).

#### Other Supplementary MAD Analyses

As reported in Online Appendix B, we observed a nonsignificant trend towards lower MADs in autistic female participants than autistic male participants in the 50 dB condition, driven by a set of posterior channels (Supplementary Fig. 7); however, no other sex comparisons closely approached statistical significance. The Bayesian analysis in Online Appendix D found significant evidence against meaningful effects of sex, or interactions of diagnostic group and sex, on MADs over frontocentral channels.

As reported in Online Appendix C, we observed no significant correlations between MADs and loudness discomfort in the TD group.

### Inter-Trial Phase Coherence (ITPC)

Clear increases in consistency of instantaneous phase were observable following stimulus onset (e.g., Fig. 3; see also Online Supplementary Materials, Appendix F, Supplementary Figs. 26–32).

**Fig. 3:**
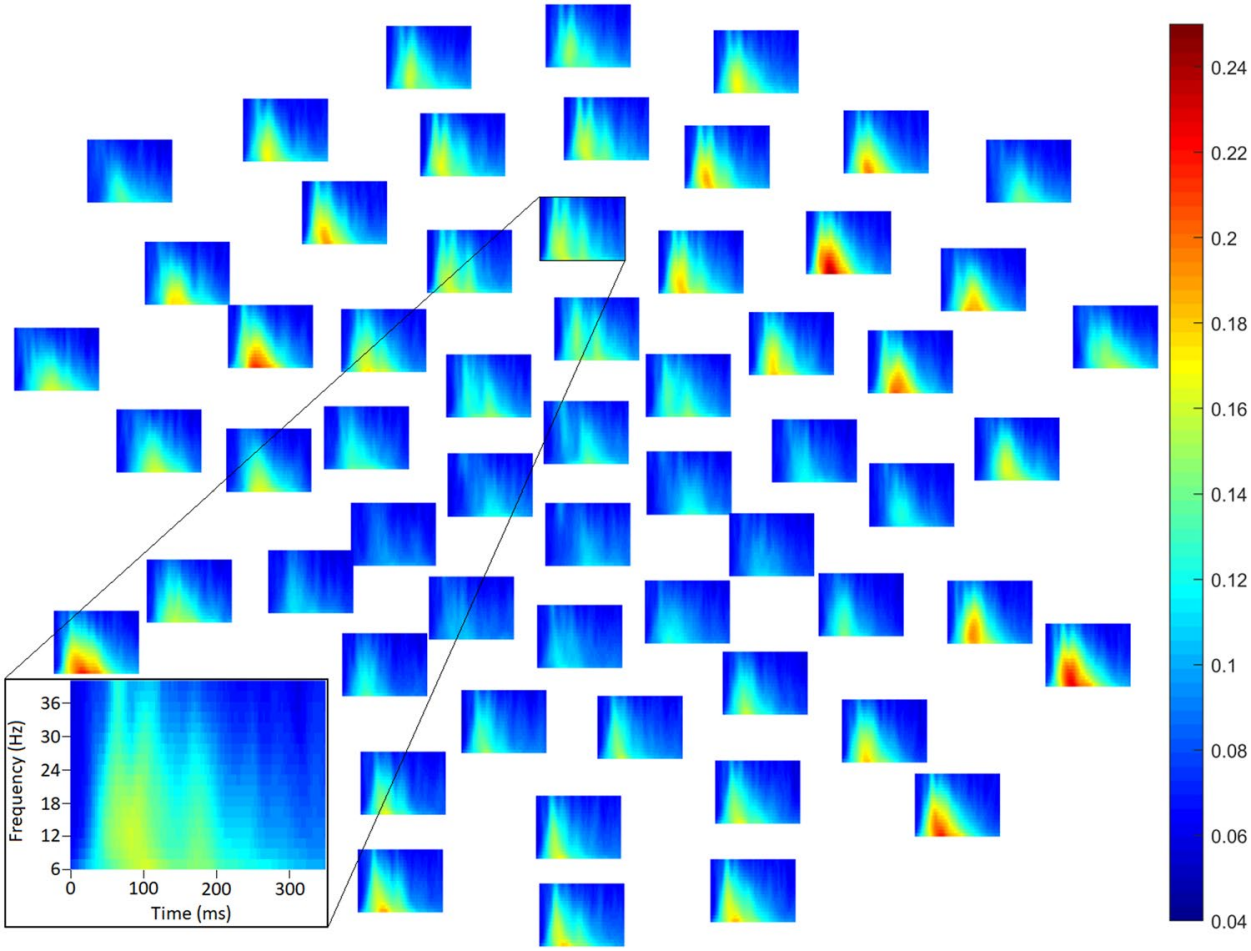
Average inter-trial phase coherence (ITPC) in the autistic group in the 80 dB intensity condition in each frequency (low at bottom, high at top) and each channel (subplots; front of head above) at time points between 1 and 350 ms (from left to right within each subplot). Channel Fz is highlighted in inset (bottom left). Larger values reflect greater consistency of instantaneous phase across trials. Substantial event-related increases in ITPC are observable, and in this sense, ITPC may capture variability—particularly latency jitter—of event-related responses. This complements MADs, which as depicted in Fig. 1 appear to reflect the overall level of variability of single-trial EEG amplitudes. Similar plots showed averaged ITPC in other group and intensity conditions are available in supplementary materials (Supplementary Figs. 5–11)

#### Group Comparisons

**Fig. 4:**
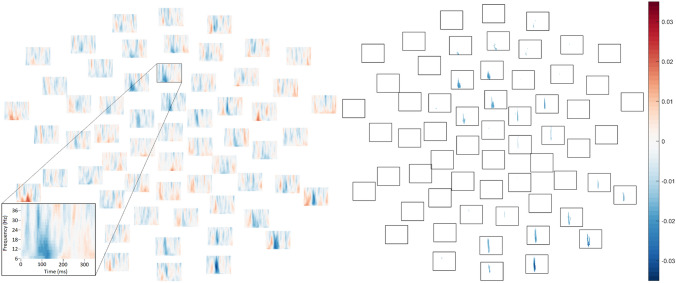
In the 50 dB intensity condition, between mean ITPC in TD group and ASD group (TD–ASD) in each frequency (low at bottom, high at top) and each channel at time points between 1 and 350 ms (from left to right). Positive values reflect greater ITPC in the typically-developing group and negative values reflect greater ITPC in the autistic group. Per the cluster-based permutation *t*-test comparing diagnostic groups, a significant cluster with greater ITPC in the ASD group was observed, *p* = .05. *Left*. Differences between mean ITPC in TD and ASD across all electrodes, time-points, and frequencies. *Right*. The boundaries of the statistically significant cluster wherein ITPC was elevated in ASD relative to TD

In the 50 dB condition, ITPC was significantly *greater* in ASD relative to TD, *p* = .048, as reflected by negative (blue) difference values over widely-distributed channels in Fig. 4 (see also Supplementary Figs. 26–27). While the boundaries of the significant effect should not be interpreted literally due to the thresholding inherent in the “cluster”-forming algorithm (see Sassenhagen & Draschkow, 2019), as well as due to limitations of time–frequency resolution, the 50 dB group difference was observed across all frequencies in a “cluster” between 101 and 163 ms, or approximately around the time period of the voltage P1 response.

**Fig. 5:**
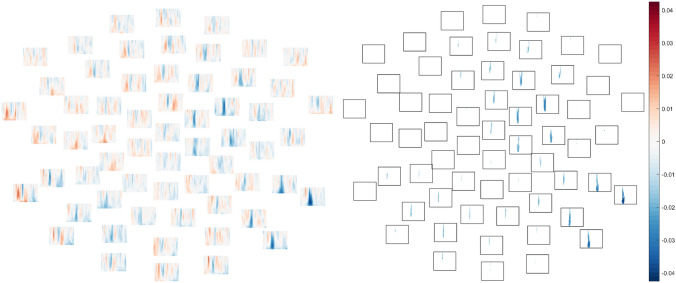
In the 60 dB intensity condition, between mean ITPC in TD group and ASD group (TD–ASD) in each frequency (low at bottom, high at top) and each channel at time points between 1 and 350 ms (from left to right). Positive values reflect greater ITPC in the typically-developing group and negative values reflect greater ITPC in the autistic group. Per the cluster-based permutation *t*-test comparing diagnostic groups, a significant cluster with greater ITPC in the ASD group was observed, *p* = .03. *Left*. Differences between mean ITPC in TD and ASD across all electrodes, time-points, and frequencies. *Right*. The boundaries of the statistically significant cluster wherein ITPC was elevated in ASD relative to TD

Furthermore, elevated ITPC was also observed in ASD relative to TD in the 60 dB condition, *p* = .028, specifically in a cluster spanning 114–160 ms, again around the time of the P1 (Fig. 5; see also Supplementary Figs. 28–29).

**Fig. 6:**
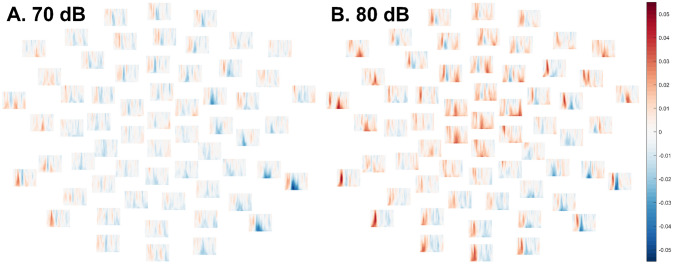
Differences between mean ITPC in TD group and ASD group (TD–ASD) in each frequency (low at bottom, high at top) and each channel at time points between 1 and 350 ms (from left to right). Positive values reflect greater ITPC in the typically-developing group and negative values reflect greater ITPC in the autistic group. **A** (left). Group differences in ITPC from the 70 dB intensity condition. No group differences approached statistical significance in the cluster-based permutation test. **B** (right). Group differences in ITPC from the 80 dB intensity condition. There was a trend for ITPC to be elevated in typically-developing relative to autistic participants, but this did not attain significance after the cluster-based permutation test, *p* = .10. Moreover, the cluster began surprisingly quickly after stimulus onset (9–101 ms)

No differences between groups approached significance in the 70 dB condition (lowest *p* = .37, Fig. 6A; see also Supplementary Figs. 30–31) or the 80 dB condition, *p* = .099 (Fig. 6B; see also Fig. 3, Supplementary Fig. 32).

#### Correlations

**Fig. 7:**
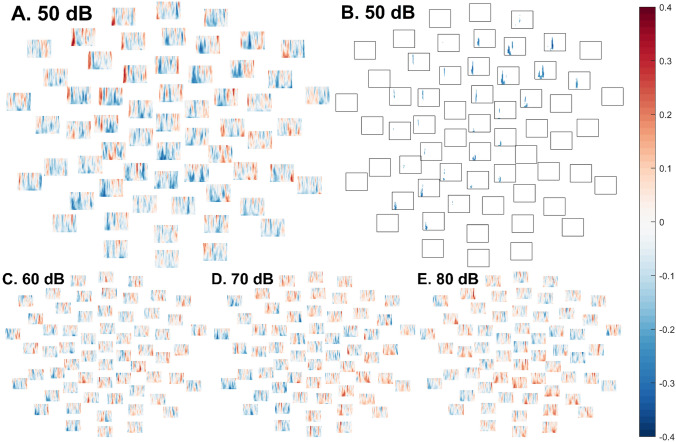
In the autistic group, spectral plots at each electrode depicting, separately in each intensity condition, Spearman’s ρ ordinal correlation coefficients between loudness discomfort and ITPC of EEG responses in each frequency (40 Hz at top; 6 Hz at bottom of each subplot) between 1 ms (left of each channel subplot) and 350 ms (right of each channel subplot). **A** (top left)*.* Spectral plots depicting correlations between ITPC of EEG responses to 50 dB tones and loudness discomfort scores. In the cluster-based permutation test, there was a significant negative cluster (reflecting greater loudness discomfort in autistic participants with lower phase coherence) in the 50 dB condition. **B** (top right). The boundaries of the statistically significant cluster wherein greater phase coherence across 50 dB trials was associated with reduced loudness discomfort in autistic participants. **C** (bottom left). Spectral plots depicting correlations between ITPC of EEG responses to 60 dB tones and loudness discomfort scores. **D** (bottom centre). Spectral plots depicting correlations between ITPC of EEG responses to 70 dB tones and loudness discomfort scores. **E** (bottom right). Spectral plots depicting correlations between ITPC of EEG responses to 80 dB tones and loudness discomfort scores

In the 50 dB condition, in the autistic group, there was a negative ordinal correlation between ITPC and SPHI scores (Fig. 7A), reflecting greater loudness discomfort in participants with reduced consistency of phase across trials, *p* = .027. While the boundaries of the effect should not be interpreted literally, the cluster spanned 38–149 ms (Fig. 7B). To aid in interpretation, supplementary, post hoc item-level analyses are presented in Online Appendix G.

In the ASD group, correlations between ITPC and SPHI loudness discomfort scores did not approach significance in the 60 dB condition (lowest *p* = .78, Fig. 7C), the 70 dB condition (lowest *p* = .87, Fig. 7D), or the 80 dB condition (lowest *p* ≥ .99, Fig. 7E).

#### Other Supplementary ITPC Analyses

As reported in Online Appendix B, we observed no significant sex differences in ITPC in either diagnostic group.

As reported in Online Appendix C, we observed no significant correlations between ITPC and loudness discomfort in the TD group.

## Discussion

Overall, the present study results provide no evidence to support the theory that inter-trial variability in sensory responses is elevated in autism. “Neural noise,” whether indexed via ITPC or MADs of EEG amplitudes across trials, was not elevated in the autistic group in any condition. Indeed, we observed enhanced ITPC—reflecting *reduced* neural noise—in autistic participants in the 50 and 60 dB intensity conditions. However, in autistic participants, we also observed a significant negative correlation between ITPC in the 50 dB condition and loudness discomfort: that is, loudness discomfort was greater in autistic participants with high inter-trial variability of EEG responses to 50 dB tones. Insofar as these ASD-TD group differences were observed in no more than two of eight comparisons (with *p*-values close to the significance threshold), and insofar as a correlation between neural noise and loudness discomfort was observed in only one of eight comparisons, the present study might be parsimoniously interpreted to suggest that there are neither clear, consistent ASD-TD group differences in neural noise, nor clear, consistent associations between the magnitude of neural noise and parent-reported loudness discomfort.

### Group Comparisons

In the present study, we found that ITPC was not significantly reduced in autism, suggesting that latency jitter of event-related responses was not increased in autism. Similarly, MADs of EEG amplitudes were not larger in autism, suggesting that variability of ongoing EEG activity is not elevated in autistic participants. Indeed, supplementary Bayesian analyses of MADs over specific frontocentral spatiotemporal windows found significant evidence against the existence of meaningful group differences. These findings would initially appear to be broadly consistent with those of Butler et al. (2017) and Randeniya et al. (2022), even though the present study was conducted in a far younger population and despite the presence of group differences in the amplitude of the N2 event-related response (see Dwyer et al., 2021a) that could theoretically have reflected inter-trial variability. In contrast, these results are clearly inconsistent with a number of prior EEG and MEG studies (e.g., Edgar et al., 2015; Kovarski et al., 2019; Milne, 2011). Further research may be necessary to determine whether these discrepancies reflect heterogeneity within autism or methodological differences between studies, such as the effectiveness of the data processing procedures used to remove recording noise.

Moreover, the present study observed apparent elevations of ITPC in autism in responses to 50 and 60 dB sounds, suggesting that neural noise was *reduced* in autism, not increased. This quite clearly contradicts the predictions of the elevated inter-trial variability account. However, in contradistinction to Davis and Plaisted-Grant (2015), we do not interpret this finding as necessarily suggestive of altered neural noise in ASD. The majority of the analyses we conducted—six of eight—showed that there were no significant group differences in metrics of inter-trial variability, and the remaining two effects only barely exceeded thresholds for statistical significance. Had a multiple comparison correction for these eight analyses been applied, the elevations of ITPC in the ASD group would no longer be considered statistically significant.

Furthermore, there exists a potential for ITPC results to be affected by differences in oscillatory power as well as by ERP amplitudes and latencies (see van Diepen & Mazaheri, 2018). The ITPC differences were observed in the approximate time window of the P1 response, and Dwyer et al. (2021a) do report—albeit in responses to louder 80 dB tones—significant group differences in P1 latencies between ASD and TD in the present study sample.

Finally, it is important to note that the reduced neural noise account of autism proposed by Davis and Plaisted-Grant (2015) focuses on “local” noise at the intra-trial level, not the inter-trial level examined in the present study. It is thus not clear that these results have direct bearing on the precise predictions advanced by Davis and Plaisted-Grant.

Importantly, the present study results apply only to neural noise in the form of random variability between responses to passive presentations of external stimuli. One form of neural noise that may be readily apparent in the subjective experiences of many autistic individuals is tinnitus, which appears to be highly prevalent in autism (Danesh et al., 2015; Williams, 2022). However, if tinnitus can be considered “neural noise,” it would seem very different in nature from inter-trial variability. As it is not linked to an external stimulus, tinnitus might be considered a form of relatively spontaneous, internal neural noise. In contrast, inter-trial variability in responses to external stimuli might seem to reflect noise/variation in the degree to which a *signal* is amplified/excited or inhibited at a given moment. Thus, if there is elevated neural noise in autism, inter-trial variability of sensory responses might not be the right domain in which to search for it. Furthermore, given present study’s passive paradigm, the auditory responses examined here can be assumed to reflect primarily bottom-up influences, consistently with how the P1 and N2 responses are commonly viewed (Donkers et al., 2015). It remains unclear whether autistic people might show “neural noise” in top-down, task-related processing, as might for example be indexed by the P3a (see Polich, 2011).

### Correlations

Unexpectedly, in autistic participants, we observed a significant negative correlation between ITPC of responses to 50 dB tones and caregiver-reported loudness discomfort, meaning that autistic participants with less consistency of instantaneous phase across trials were reported to experience greater sensory distress. As 50 dB was the softest intensity at which tones were presented in this study, available sensory signal would presumably be relatively low compared to other conditions, allowing neural noise to exert a greater relative influence on the consistency of the timing of the event-related response across trials. This more variable sensory brain response could lead to subjective instability of the sensory experience, producing discomfort. However, it seems counterintuitive that noisy, inconsistent brain responses to *soft* sounds would result in a phenotype of sensory distress and discomfort more so than for responses to *loud* sounds. If the association observed in the present study is genuine, it might instead reflect difficulties filtering out soft sounds, perhaps related to misophonia (Williams et al., 2021c).

It also appears somewhat counterintuitive that autistic people—who experience greater sensory sensitivities than non-autistic people—would have greater ITPC of responses to 50 dB tones than non-autistic people, as we observed in our group comparisons, if reduced ITPC of such responses is indeed linked to heightened sensory sensitivity. In this light, it is important to note that there were no significant associations between loudness discomfort and ITPC in the 60, 70, or 80 dB conditions, nor were there significant associations between MADs of EEG responses and loudness discomfort in any intensity condition. Thus, a significant negative correlation between ITPC and loudness discomfort was observed in only one of the eight associations we examined, and this effect would not have survived a correction for multiple comparisons. We therefore find it difficult to be confident that either the ASD-TD group differences or the association between ITPC and loudness discomfort are real effects. Autistic experiences of sensory discomfort and overload might well reflect other mechanisms, such as heightened attentional vigilance, heightened susceptibility towards attentional capture, heightened emotional or autonomic responses, or reduced habituation to repeated stimuli (Williams et al., 2021c).

### Limitations

We believe that the present study has a number of strengths, particularly its large sample, the large number of trials obtained from each participant, and the rigorous data cleaning pipeline used to remove putatively non-neural, recording-related noise that might confound inter-trial variability analyses. However, we do wish to draw readers’ attention to some limitations of our study.

First, the present study’s epochs (− 200 ms to 599 ms) are, while similar to or better than those of some prior studies of intra-individual neural noise in ASD, relatively short for time–frequency analyses on ITPC. Unfortunately, there is little temporal room to expand epochs, as the shortest interstimulus intervals were only 1000 ms and as our independent components analysis requires a further lagged period (in this study, 300 ms, or through to 899 ms post-stimulus) for examining the covariance of signals. This reflects the fact that the present paradigm was designed with the requirements of ERP analyses, not time–frequency analyses, in mind. However, the main effects of this reduced range are to limit our time–frequency resolution and to prevent us from examining frequencies below ~ 6 Hz. We do not believe these restrictions compromise our ability to achieve our aim, which is simply to search for overall group differences in inter-trial variability and “neural noise” rather than to isolate them to particular frequencies.

Second, the present study data were collected in a relatively uncontrolled environment. Participants passively listened to auditory tones while watching a quiet video. The quiet video was not identical across participants, and in the absence of any clear task, different participants may have varied considerably in which aspects of their environments they attended to at any given moment, as well as any other thoughts or cognitive processes they may have been engaging in. All of these factors could have contributed to and influenced the variability of the EEG signal across trials. However, the present study’s less controlled environment could also be seen as an advantage, insofar as real-world settings outside the laboratory are often similarly uncontrolled.

Third, a large number of participants—particularly autistic participants—were excluded from the present study due to poor-quality EEG data. Analyses in supplementary materials indicate that autistic participants who provided incomplete or otherwise poor-quality EEG data exhibited lower cognitive abilities, on average, than autistic participants who provided usable EEG data. While the present study’s diverse range of cognitive abilities ensures that participants with lower cognitive ability scores are nevertheless well-represented, in contrast to much prior autism research that excludes participants with intellectual disabilities (Russell et al., 2019), it remains possible that autistic participants excluded from the study may have systematically differed from retained participants in other, unmeasured variables.

Fourth, our analyses did not control for sex, which differed across our diagnostic groups, although exploratory analyses of sex differences are presented in supplementary materials (Online Appendix B).

Finally, due to the young age of our participants, and their diverse cognitive and language abilities, we relied on a parent-report measure of sensory behaviour. Prior autism research indicates that parent reports are only moderately correlated with self-report sensory measures, and unlike self-reports, they fail to converge with changes in heart rate during noise exposure (Keith et al., 2019). This may reflect parents’ lack of direct access to their children’s internal, conscious experiences. In future research with young children, measures of physiological reactivity to sensory stimuli might provide an important complement to parent-report measures. Such measures could perhaps be collected in concert with structured observations of behavioural reactivity to stimuli.

### Summary

The present study finds no statistically significant evidence to support the theory that autism is characterized by or associated with elevated “neural noise” in the form of inter-trial variability of brain responses. On the contrary, the present study finds significantly greater ITPC in autistic participants in responses to softer 50 dB and 60 dB sounds, which could be interpreted as a sign of reduced neural noise in ASD. However, we believe it would be premature to suggest that the theory that neural noise is reduced in ASD has any robust empirical support from the present study, insofar as there were no significant between-group differences in the majority of the inter-trial variability analyses included in this report. Furthermore, although we did observe a significant association between heightened loudness discomfort and reduced phase coherence of responses to 50 dB tones in autistic participants, no significant correlations were detected in any other test we examined. Thus, we similarly believe it would be premature to suggest that subjective sensory sensitivity in autism is driven by elevated neural noise. We believe the most parsimonious interpretation of the present study results is that inter-trial variability of brain responses in ASD and TD is broadly comparable, and that such inter-trial variability has no consistent relationship with subjective sensory distress. Future research aiming to find evidence of elevated “neural noise” in autism might find it useful to consider examining ongoing, internal noise as manifested in experiences such as tinnitus and visual snow, as well as variability in the neural correlates of top-down, task-related processes, rather than inter-trial variability in responses to passively-presented external stimuli.

### Supplementary Information

Below is the link to the electronic supplementary material.

Supplementary file1 (DOCX 12446 KB)

## Footnotes

1 Autism identifying language remains a controversial area, including among individuals on the autism spectrum themselves (Bury et al., 2020). In deference to the preferences of many individuals on the autism spectrum (Bury et al., 2020; Kenny et al., 2016), and in light of concern that person-first language may be conducive towards (or at least reflective of) stigma (Gernsbacher, 2017), we have chosen to use identity-first language (e.g., “autistic person”). We have also chosen to use neutral descriptive terms such as “autism spectrum development” in preference to value-laden terms like “disorder” or “condition,” again partly in deference to autistic opinion, but also out of a desire to avoid subjective value judgements in academic terminology (Dwyer et al., 2022).

2 Puts et al. (2017) did find that high levels of GABA, generally an inhibitory neurotransmitter, are related to poorer tactile acuity in ASD. However, in this study, the effect of GABA appears to have reflected inhibition specifically related to prior subthreshold tactile stimulation, not general stochastic resonance; no association was observed between GABA and tactile acuity in the absence of prior subthreshold tactile stimulation.

3 The median absolute deviation was selected as an index of variability in preference to the standard deviation, which is vulnerable to outliers, as well as in preference to coefficients of variation that are normalized by medians or means. EEG voltages can be either positive to negative, and often approach zero, complicating any attempt to calculate a coherent mean or median; while one could still add a constant to bring all voltage values above zero, varying the magnitude of the constant would then change the coefficient of variation. See Al-Jawahiri et al. (2019) for another example of the MAD being used as an index of variability in EEG amplitudes.
