# Supplementary material for: “Neural Noise” in Auditory Responses in Young Autistic and Neurotypical Children
Source: J Autism Dev Disord. Author manuscript; Available in PMC 2024 Feb 16. (PMC10209352; doi:10.1007/s10803-022-05797-4)
Supplement: Supplemental File 1 (includes all supplemental material) [file NIHMS1894919-supplement-Supplemental_File_1__includes_all_supplemental_material_.docx]

# Appendix A. Characteristics of Participants with Unusable EEG Data

We carried out a supplementary analysis to compare the 127 autistic and 79 typically-developing participants included in EEG analyses in the main text with autistic and typically-developing participants excluded from the study due to bad EEG data. The goal of this analysis was to determine whether there were variables associated with provision of poor-quality EEG data in the study.

Specifically, in the bad-data sample from this analysis, we examined 83 autistic and 21 typically-developing participants. This group comprised the 47 participants (40 ASD, 7 TD) with incomplete recordings, the 47 participants (37 ASD, 10 TD) with poor-quality recordings, the 5 sleepy participants (3 ASD, 2 TD), and the 5 participants (3 ASD, 2 TD) with discontinuities in trial-by-trial global field power.

Participants excluded due to technical issues, neuroanatomical abnormalities, and ineligibility for the study sample were not examined here, as the quality of their EEG data was not related to the reasons for their exclusion.

Ordinal Wilcoxon tests were conducted separately in each diagnostic group. Cliff’s delta is reported as an effect size.

We chose to examine the following measures:

- Sensory Profile Hyperacusis Index (SPHI) scores, in case loudness discomfort caused by tone stimuli may have caused participants to enter sensory distress and/or sensory meltdowns.
- Short Sensory Profile (SSP) tactile sensitivity scores, in case tactile sensitivities related to EEG capping may have similarly caused sensory distress to some participants. Note that tactile sensitivity scores do not reflect the original scale described by McIntosh and colleagues (1999); the scores are based on the factor analysis reported by Williams and colleagues (2018), reverse-scored for consistency with SPHI, such that higher scores reflect greater tactile sensitivity.
- Standard scores reflecting cognitive ability (Mullen Scales Developmental Quotient) and adaptive behaviour (Vineland-II composite) scores, in case participants with higher or lower levels of cognitive ability or adaptive behaviour found the process of EEG capping and recordings more challenging.
- Internalizing and externalizing behaviour T-scores from the Childhood Behaviour Checklist, in case participants exhibiting these patterns reacted more negatively to EEG capping and recordings.

## Short Sensory Profile (SSP) Results

Autistic participants with and without good EEG data did not significantly differ in their estimated loudness discomfort scores from the Sensory Profile Hyperacusis Index (SPHI), Wilcoxon *p* = .21, Cliff’s δ = .12 (*Supplementary Figure 1*). Furthermore, we observed no differences in SSP tactile sensitivity scores between autistic participants with and without usable EEG data, Wilcoxon *p* = .18, Cliff’s δ = 13 (*Supplementary Figure 2*).

Typically-developing participants with and without good EEG data also did not significantly differ in loudness discomfort, Wilcoxon *p* = .55, Cliff’s δ = –.09 (*Supplementary Figure 1*), or tactile sensitivity, Wilcoxon *p* = .74, Cliff’s δ = –.05 (*Supplementary Figure 2*).

## Mullen Scales Developmental Quotient (DQ) Results

Autistic participants with good EEG data had significantly higher Mullen DQ scores than autistic participants who provided bad EEG data, Wilcoxon *p* = .002, Cliff’s δ = .25 (*Supplementary Figure 3*). This finding should be interpreted with some caution, as participants who do not cooperate with attempts at EEG data collection may also be less interested in complying with researcher demands in the context of an assessment such as the Mullen Scales.

Typically-developing participants with and without good EEG data did not significantly differ in their DQ, Wilcoxon *p* = .29, Cliff’s δ = .16 (*Supplementary Figure 3*). This may reflect the more restricted range of cognitive ability scores in the TD group.

## Vineland Adaptive Behavior Scales (VABS)-II Composite Standard Scores Results

Autistic participants with and without good EEG data did not significantly differ in their VABS-II composite scores, Wilcoxon *p* = .97, Cliff’s δ = –.003 (*Supplementary Figure 4*).

Typically-developing participants with and without good EEG data also did not significantly differ in VABS-II composite scores, Wilcoxon *p* = .88, Cliff’s δ = –.02 (*Supplementary Figure 4*).

## Childhood Behavior Checklist (CBCL) Results

Autistic participants with and without good EEG data did not significantly differ in their CBCL internalizing scores, *p* = .91, Cliff’s δ = –.01 (*Supplementary Figure 5*), or their externalizing scores, *p* = .28, Cliff’s δ = .09 (*Supplementary Figure 6*).

Despite some strong trends, typically-developing participants with and without good EEG data also did not significantly differ in CBCL internalizing scores, *p* = .09, Cliff’s δ = .24 (*Supplementary Figure 5*), or their CBCL externalizing scores, *p* = .057, Cliff’s δ = .28 (*Supplementary Figure 6*). The direction of the observed trends in the TD group was surprising to us: typically-developing participants with *good* EEG data appeared to have *more* internalizing and externalizing behaviour problems according to their parents’ reports.

| 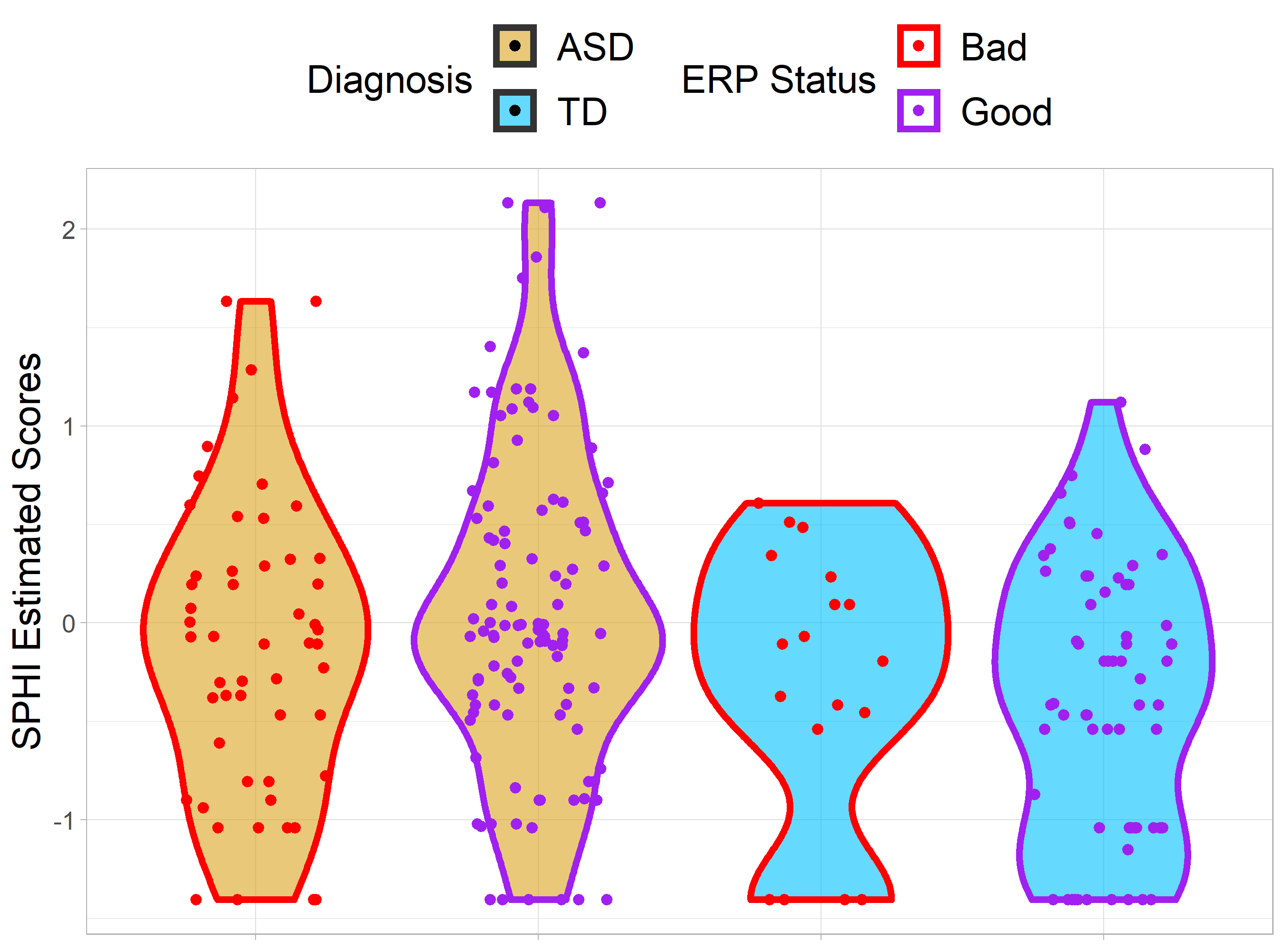 |
| --- |
| *Supplementary Figure 1*. Loudness discomfort scores by diagnostic group and by whether EEG data were recorded successfully or not. Higher scores reflect greater loudness discomfort. Participants with and without successfully-recorded EEG data did not differ in their loudness discomfort scores in either diagnostic group. |

| 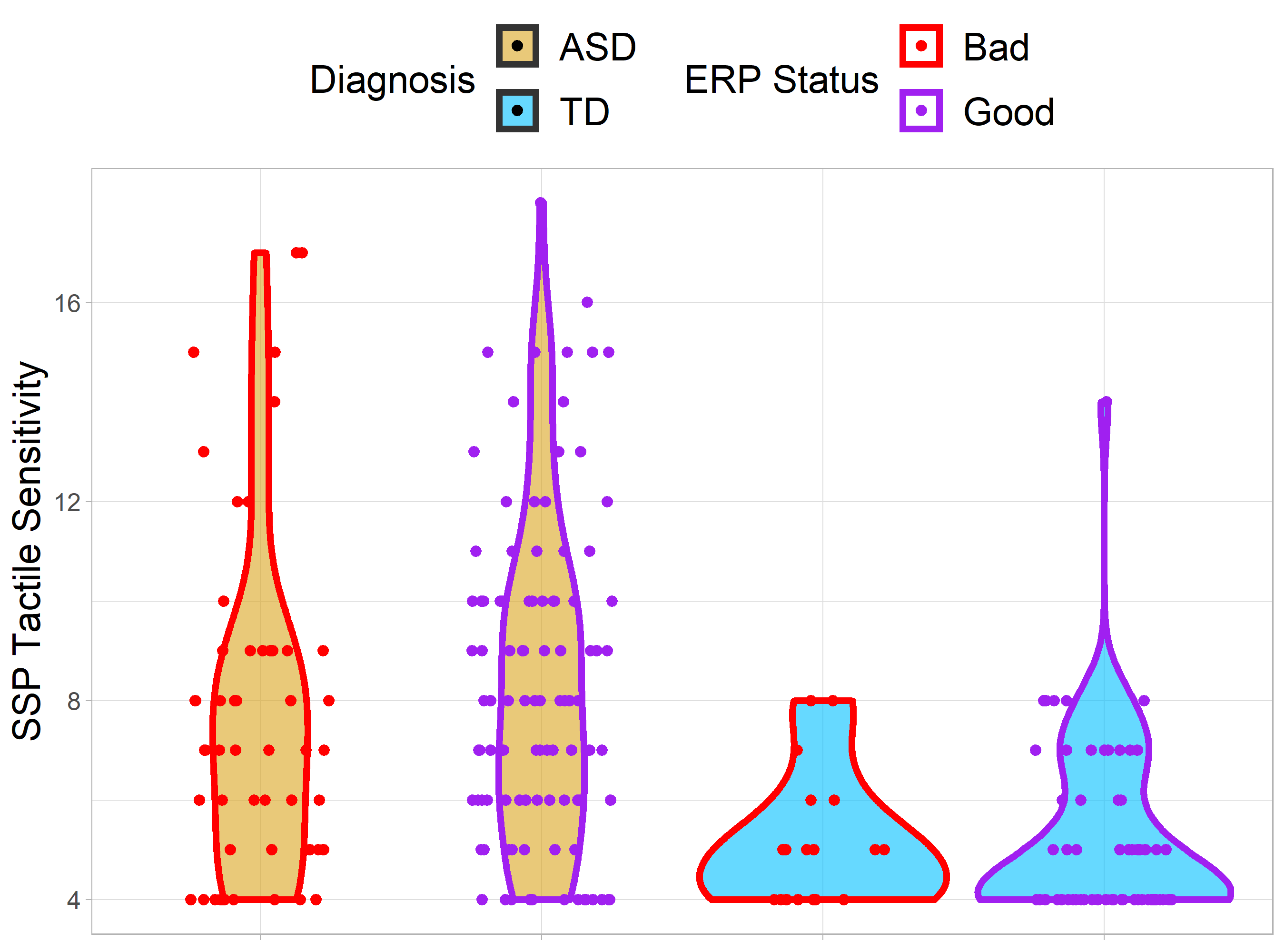  *Supplementary Figure 2*. Tactile sensitivity scores by diagnostic group and by whether EEG data were recorded successfully or not. Higher scores reflect greater tactile sensitivity. Participants with and without successfully-recorded EEG data did not differ in their tactile sensitivity scores in either diagnostic group. |
| --- |

| 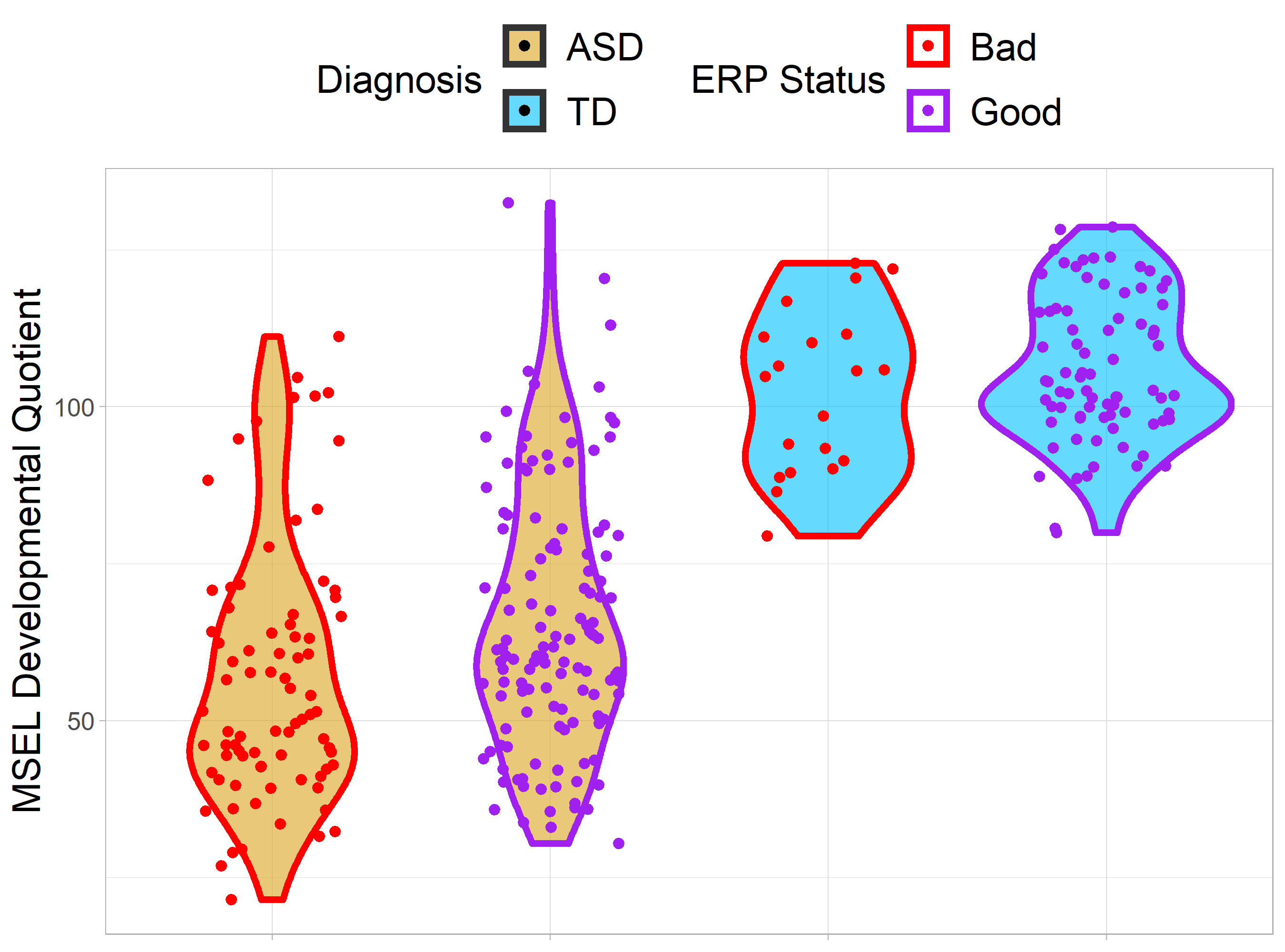 *Supplementary Figure 3*. Cognitive ability scores by diagnostic group and by whether EEG data were recorded successfully or not. In the autism group, participants with usable EEG data had higher cognitive ability scores than participants from whom EEG data were not collected. Typically-developing participants with and without successfully-recorded EEG data did not differ in cognitive ability. |
| --- |

| 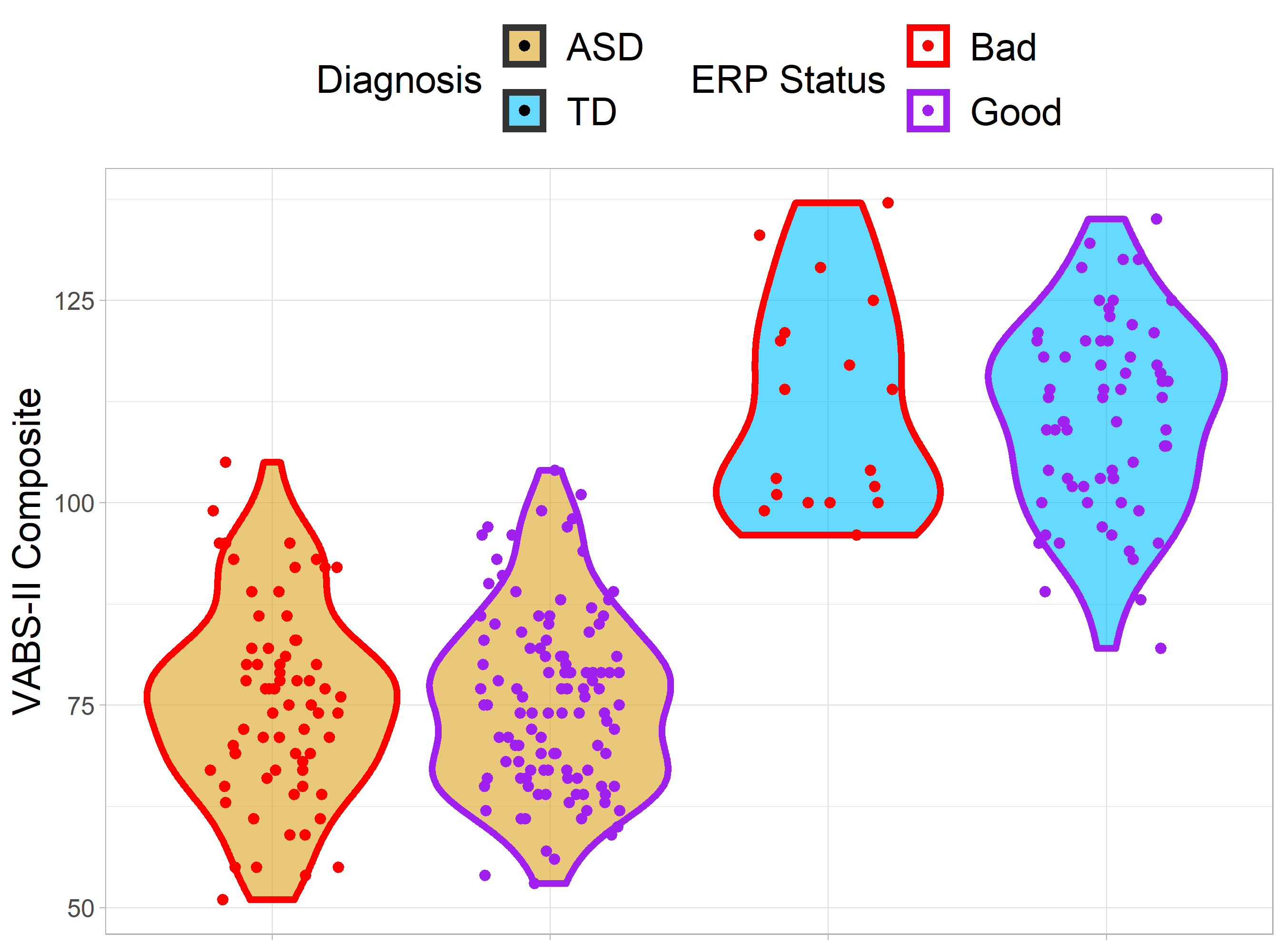 *Supplementary Figure 4*. Adaptive behaviour composite scores by diagnostic group and by whether EEG data were recorded successfully or not. Participants with and without successfully-recorded EEG data did not differ in their adaptive behaviour scores in either diagnostic group. |
| --- |

| 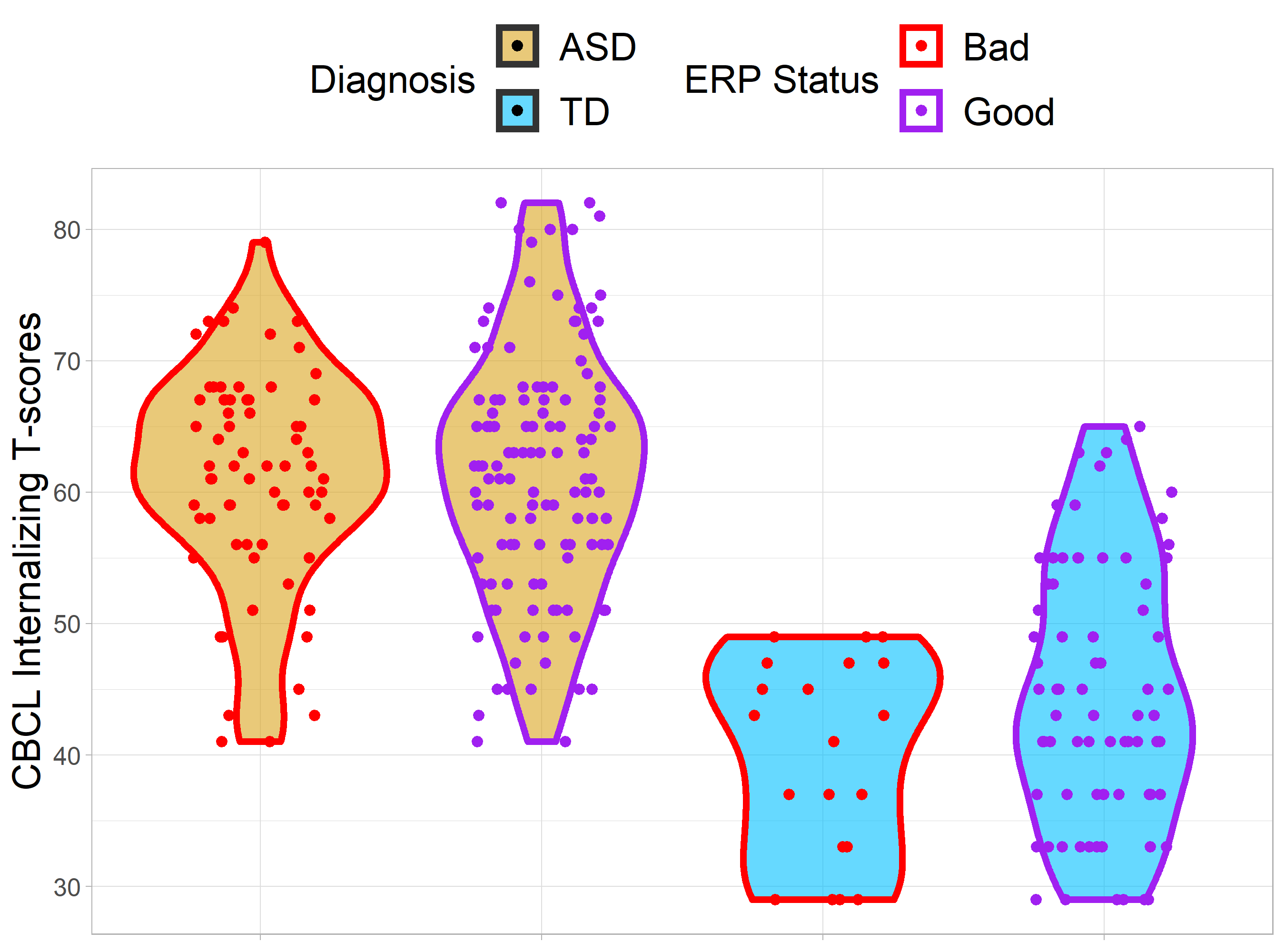 *Supplementary Figure 5*. Internalizing scores by diagnostic group and by whether EEG data were recorded successfully or not. Participants with and without successfully-recorded EEG data did not significantly differ in their internalizing scores in either diagnostic group, despite a trend for typically-developing children with usable EEG data to have higher levels of parent-reported internalizing behaviour. |
| --- |

| 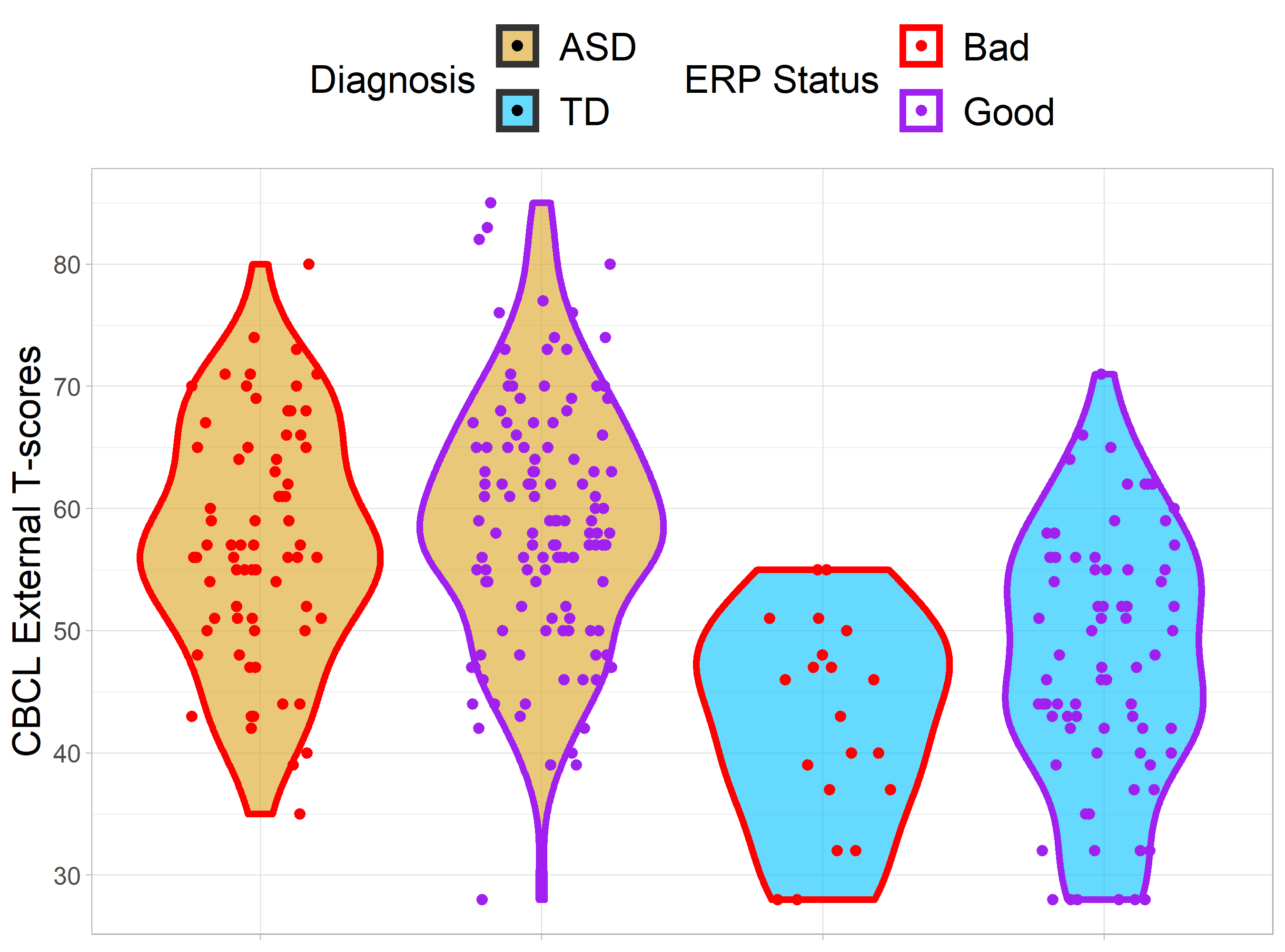 *Supplementary Figure 6*. Externalizing scores by diagnostic group and by whether EEG data were recorded successfully or not. Participants with and without successfully-recorded EEG data did not significantly differ in their externalizing scores in either diagnostic group, despite a strong trend for typically-developing children with usable EEG data to have higher levels of parent-reported externalizing behaviour. |
| --- |

# Appendix B. Sex Differences

We carried out supplementary analyses to compare male and female participants based on their ITPC and MAD values.

## Median Absolute Deviations (MADs)

For analyses of sex differences in MADs, we employed essentially the same statistical approach that was used to compare MADs across diagnostic groups in the main text. MADs were examined in time points between 1 and 350 ms post-stimulus, and cluster-based permutation t-tests (Maris & Oostenveld, 2007) with 10,000 permutations were used to compare male and female participants, separately in each diagnostic group and intensity condition.

In the ASD group, there was a nonsignificantly trending difference between male and female participants in the 50 dB condition, *p* = 08, driven by posterior channels (*Supplementary Figure 7*). This appeared to reflect lower median absolute deviations, i.e., reduced inter-trial variability, in female participants. Similar patterns were visually apparent in other conditions (*Supplementary Figures 8-10*), but differences did not closely approach statistical significance in the 60 dB condition, *p* ≥. 16, the 70 dB condition, *p* ≥ .13, or the 80 dB condition, *p* ≥ .14.

Although these sex differences over posterior channels were not statistically significant and although the analysis of sex differences in MADs involved eight multiple comparisons (2 groups*4 intensities), if the effects do reflect some systematic pattern and not random chance, it seems unlikely that this pattern would be attributable to auditory event-related responses. Not only was the MAD not clearly modulated by event-related responses (as depicted in *Figure 1* of the main text), suggesting it may relate to endogenous noise rather than variability in stimulus-evoked responses, but the topography of a posterior effect would be inconsistent with the frontocentral topography of tangential dipoles originating in auditory cortex, or the temporal topography of radial dipoles from the same location (see Ponton et al., 2002). While the average reference used in the present study could lead to partial subtraction of responses centred elsewhere from posterior scalp channels, this would not explain a posterior-*only* distribution. An effect with a posterior/occipital topographic distribution might be expected to be more related to processing of visual stimulation from the quiet movies viewed by participants. However, attributing a trending effect in the MAD to visual stimuli seems inconsistent with the general lack of event-relatedness observed in the MAD generally. It is possible that the trending sex difference in ASD, if not artefactual and if not due to chance, could instead reflect variability of EEG oscillations such as occipital alpha rhythms.

In the TD group, we observed no significant differences between male and female participants in any intensity condition, all cluster-based permutation *p* > .99 (*Supplementary Figure 11*).

## Inter-Trial Phase Coherence (ITPC)

Again, the methodological approach we use in this analysis was identical to the analyses comparing ITPC across both diagnostic groups, as described in the main text: ITPC was examined in frequencies falling between 6 and 40 Hz, with 2 Hz steps between each frequency, and time points between 1 and 350 ms, and cluster-based permutation t-tests (Maris & Oostenveld, 2007) were used to compare male and female participants, separately in each diagnostic group and intensity condition.

In the ASD group, we observed no statistically significant ITPC differences between male and female participants in the 50 dB condition, *p*≥.20 (*Supplementary Figure 12,* left), the 60 dB condition, *p*≥.32 (*Supplementary Figure 13,* left), the 70 dB condition, *p*≥.24 (*Supplementary Figure 14,* left), or the 80 dB condition, *p*≥.29 (*Supplementary Figure 15,* left).

In the TD group, we also observed no statistically significant ITPC differences between male and female participants in the 50 dB condition, *p*≥.60 (*Supplementary Figure 12*, right), the 60 dB condition, *p*≥.77 (*Supplementary Figure 13*, right), the 70 dB condition, *p*≥.51 (*Supplementary Figure 14*, right), or the 80 dB condition, *p*≥.44 (*Supplementary Figure 15*, right).

| 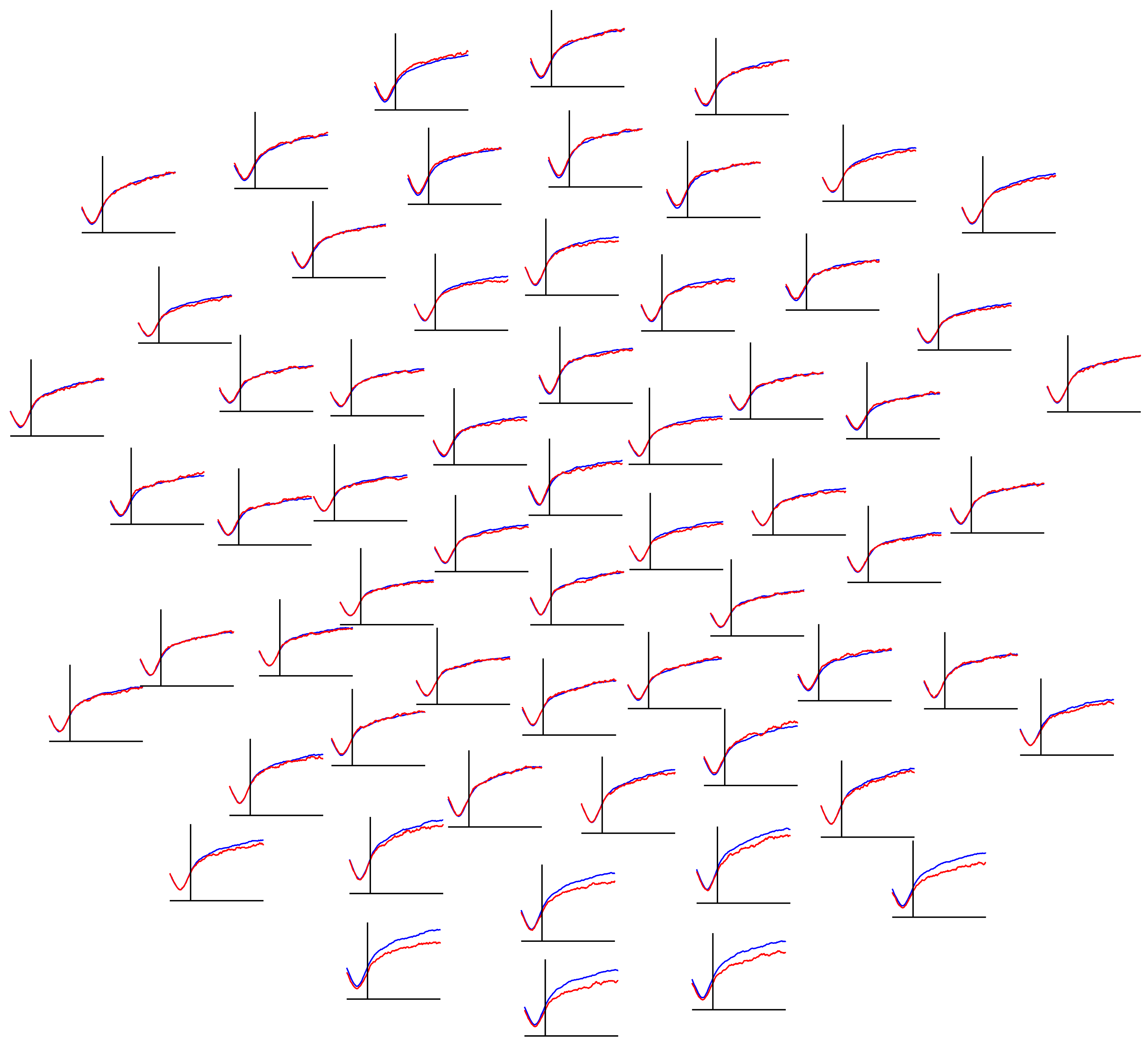 *Supplementary Figure 7*. In the **autistic** group and in the **50 dB** condition, waveforms at each electrode depicting sex differences in median absolute deviations of EEG amplitudes across trials between 1 ms (left of each channel subplot) and 350 ms (right of each channel subplot). MAD values on the Y-axis of each subplot range from 2.5 to 25.0. **Female** participants are marked by **red lines**, while **male** participants are marked by **blue lines**. There was a nonsignificant trend towards a sex difference over occipital channels, *p* = .08. |
| --- |

| 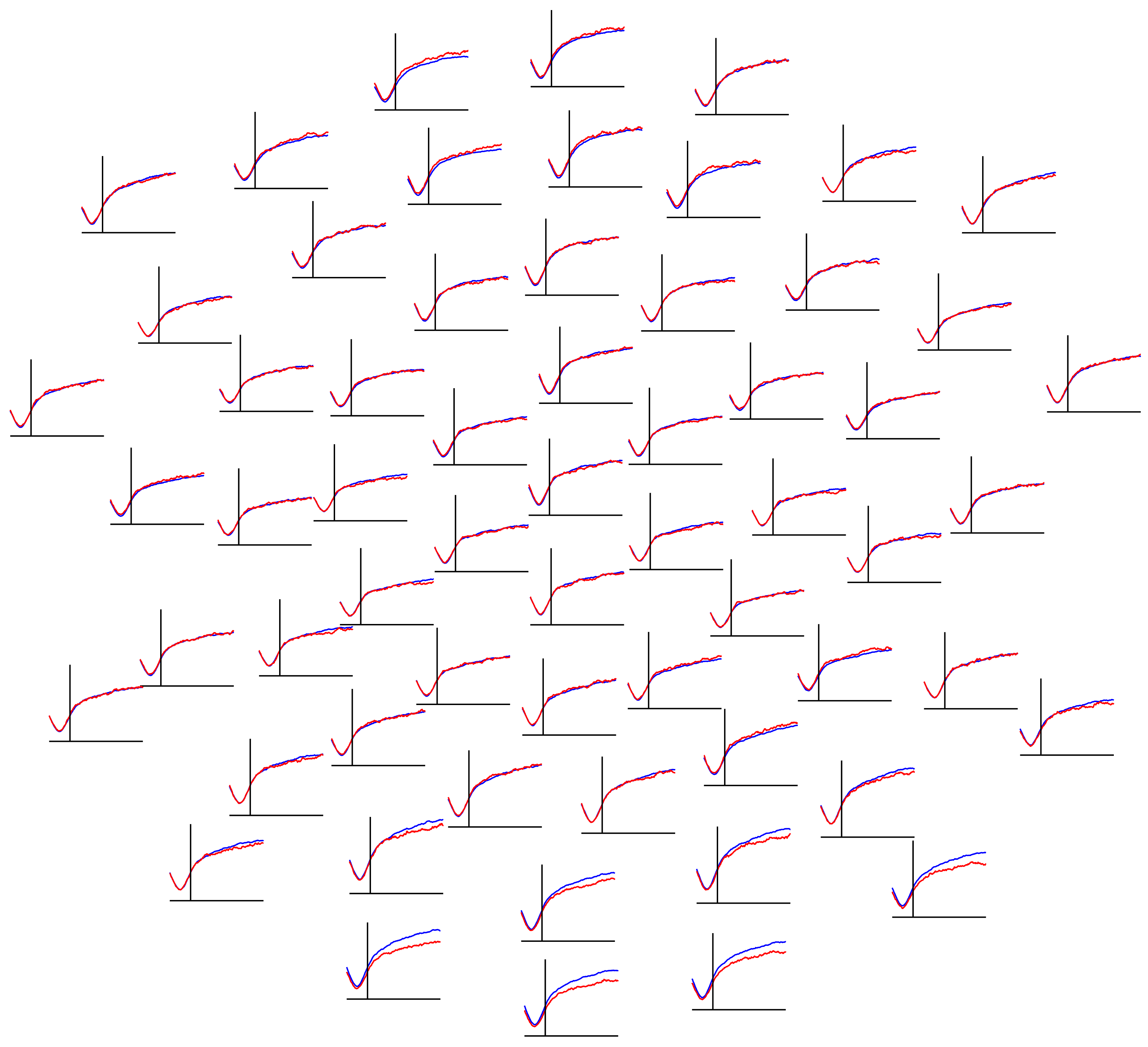*Supplementary Figure 8*. In the **autistic** group and in the **60 dB** condition, waveforms at each electrode depicting sex differences in median absolute deviations of EEG amplitudes across trials between 1 ms (left of each channel subplot) and 350 ms (right of each channel subplot). MAD values on the Y-axis of each subplot range from 2.5 to 25.0. **Female** participants are marked by **red lines**, while **male** participants are marked by **blue lines**. |
| --- |

| 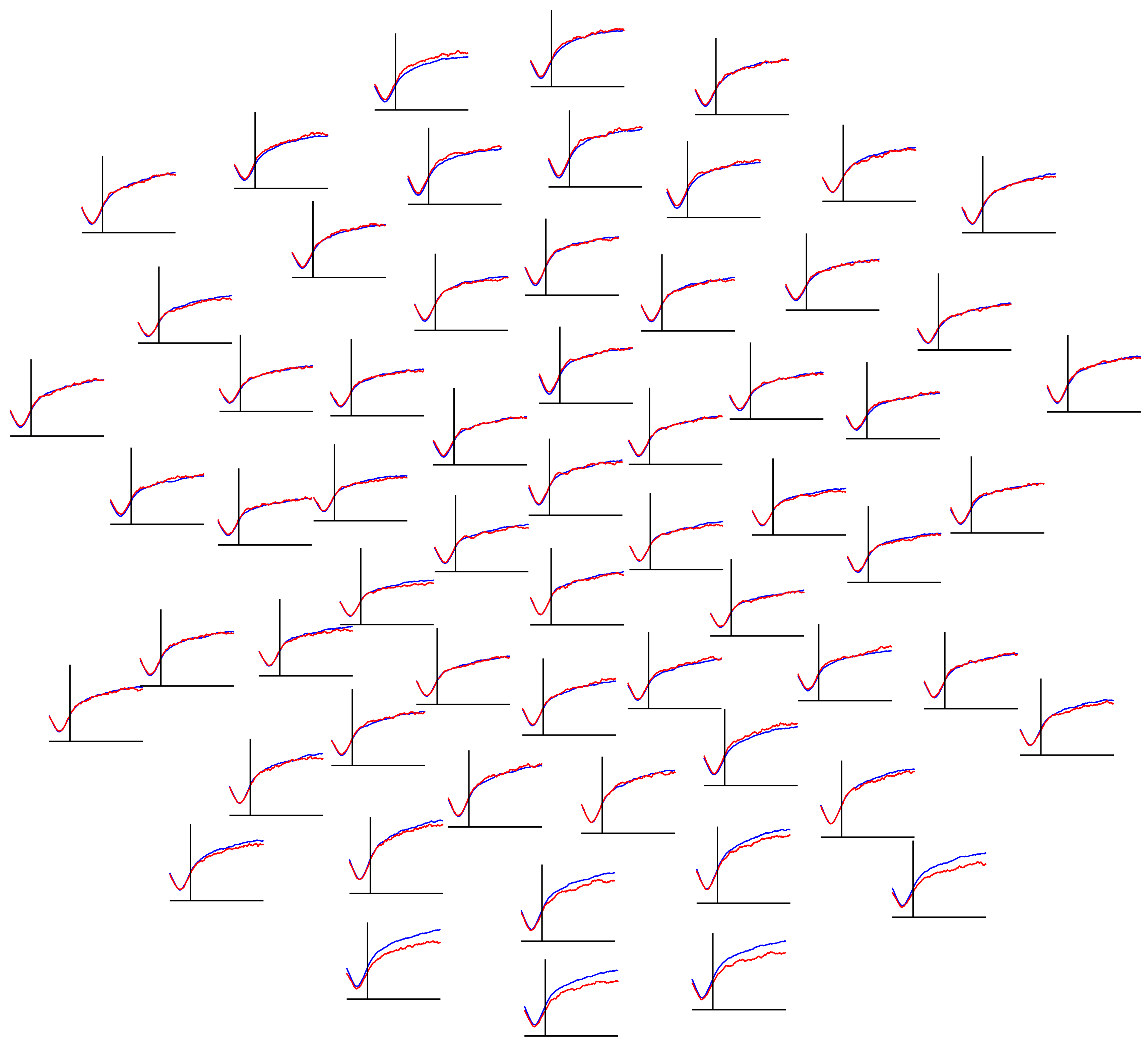*Supplementary Figure 9*. In the **autistic** group and in the **70 dB** condition, waveforms at each electrode depicting sex differences in median absolute deviations of EEG amplitudes across trials between 1 ms (left of each channel subplot) and 350 ms (right of each channel subplot). MAD values on the Y-axis of each subplot range from 2.5 to 25.0. **Female** participants are marked by **red lines**, while **male** participants are marked by **blue lines**. |
| --- |

| 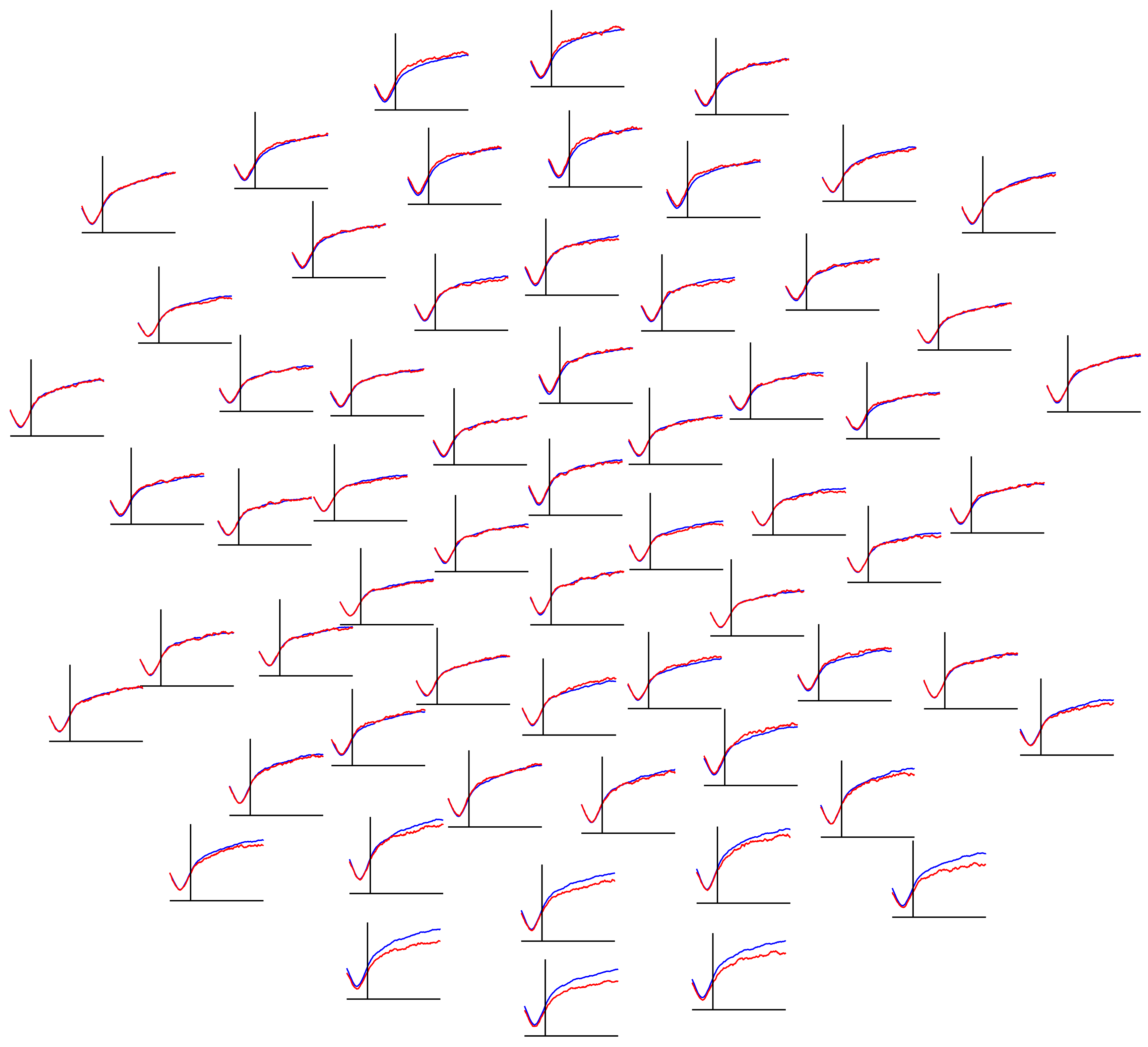*Supplementary Figure 10*. In the **autistic** group and in the **80 dB** condition, waveforms at each electrode depicting sex differences in median absolute deviations of EEG amplitudes across trials between 1 ms (left of each channel subplot) and 350 ms (right of each channel subplot). MAD values on the Y-axis of each subplot range from 2.5 to 25.0. **Female** participants are marked by **red lines**, while **male** participants are marked by **blue lines**. |
| --- |

| 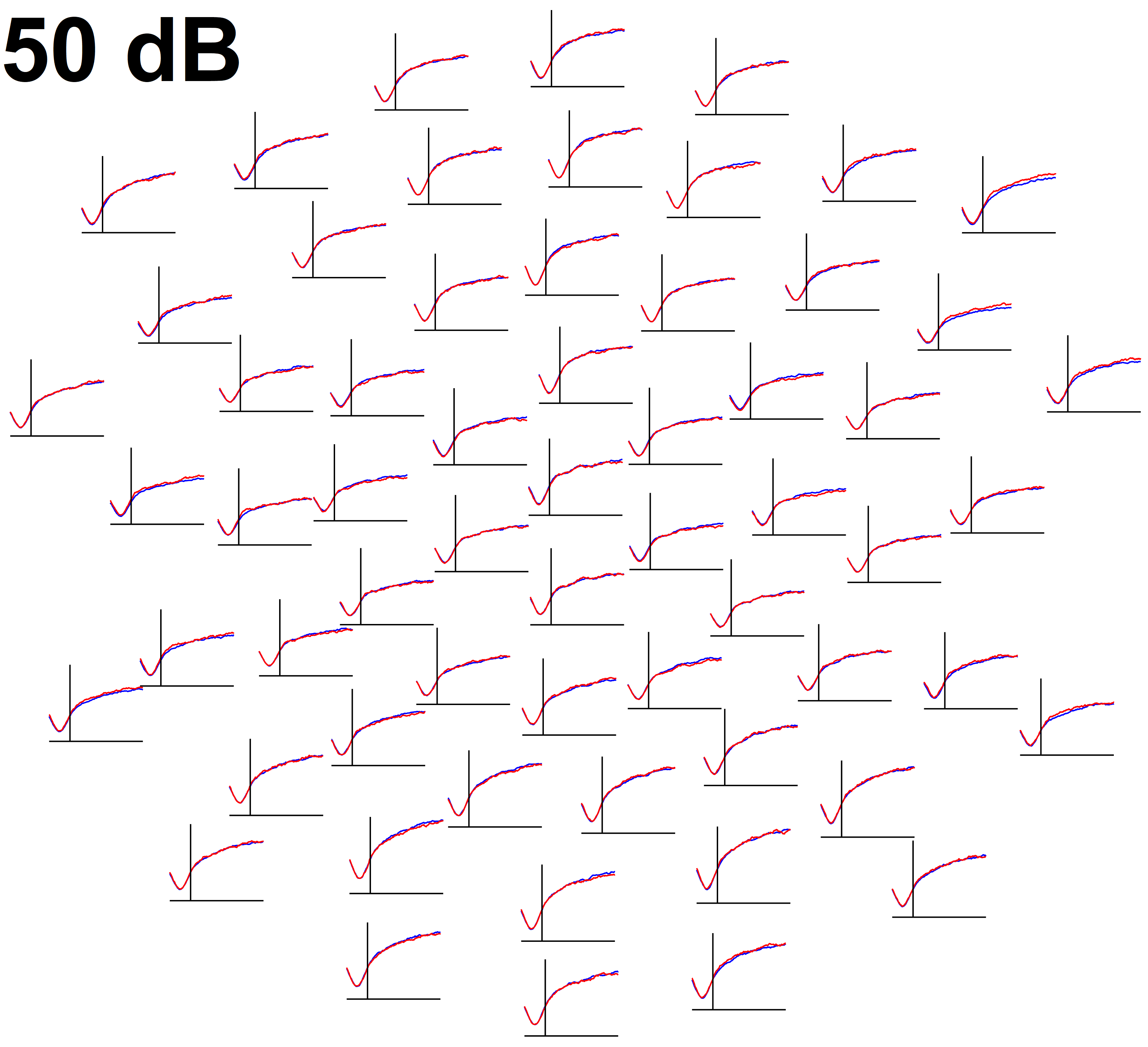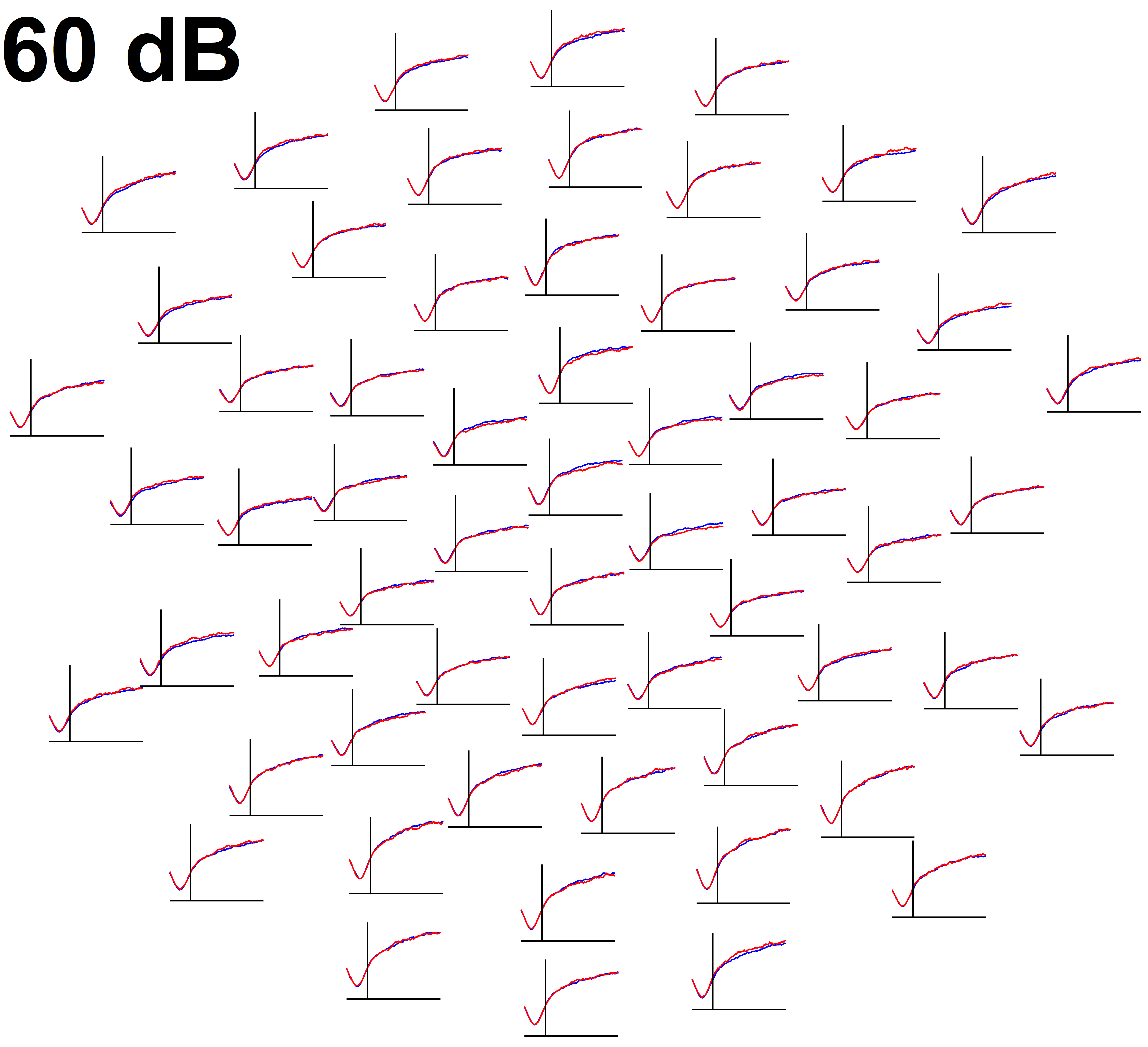  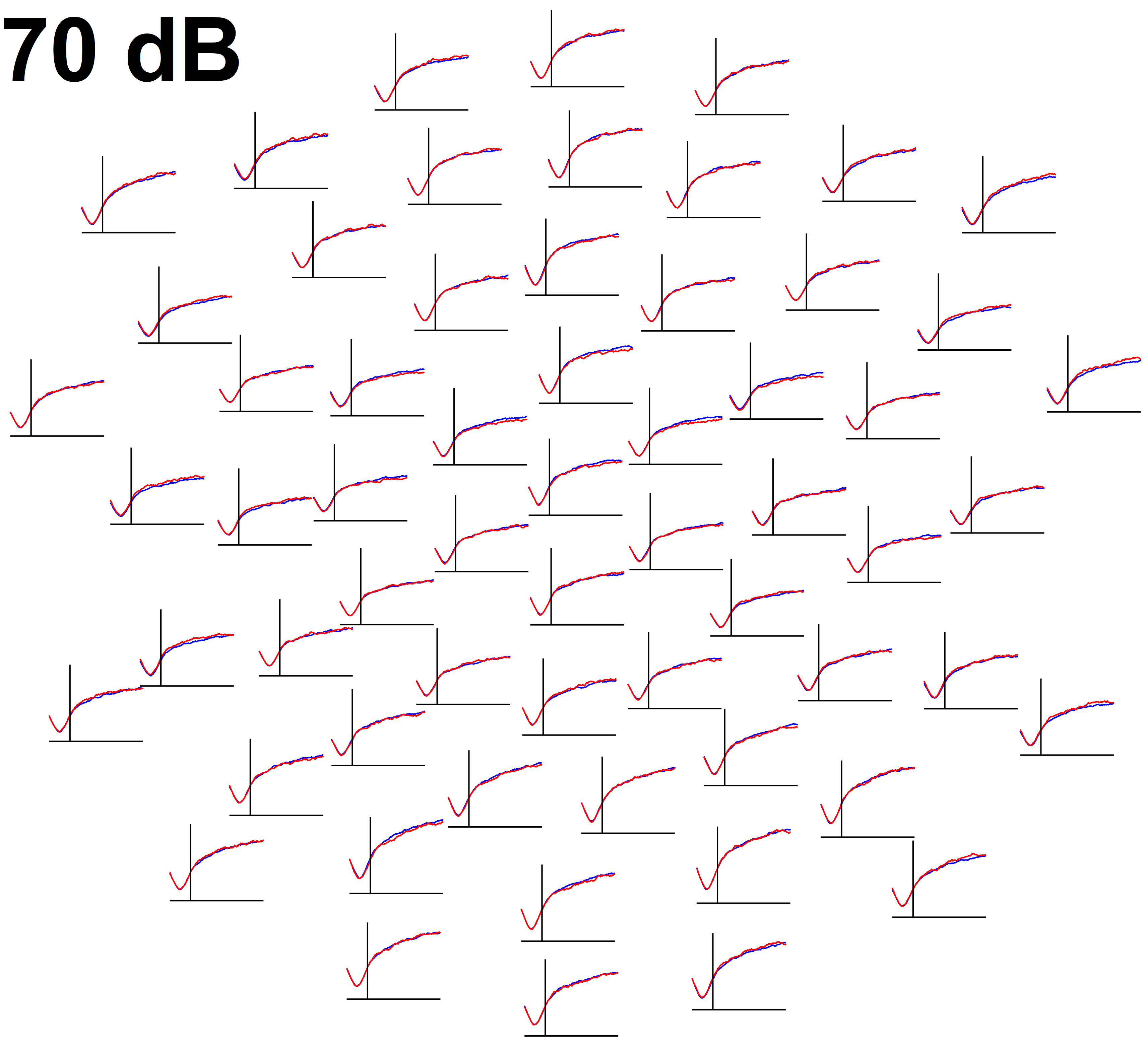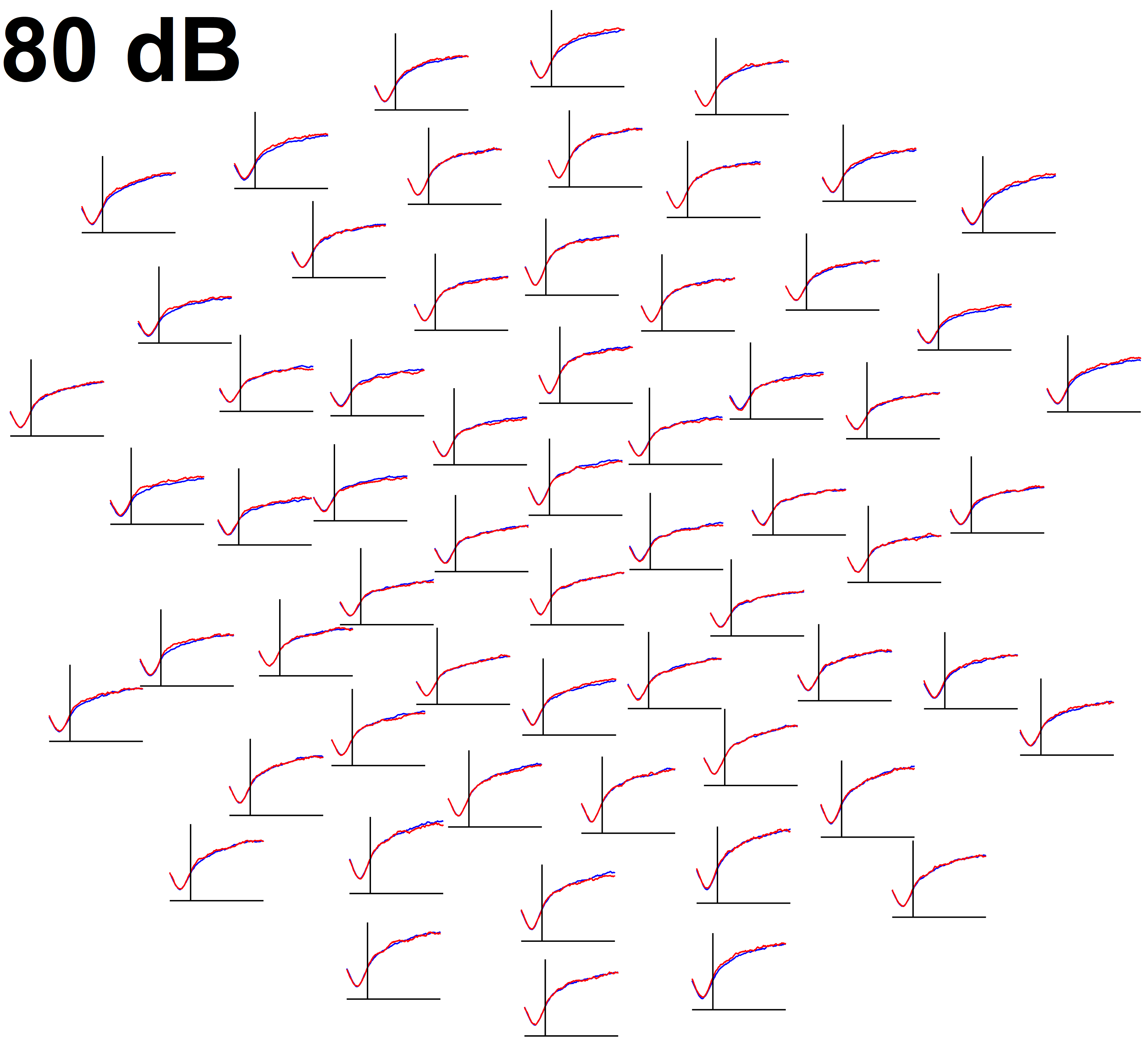  *Supplementary Figure 11*. In the **typically-developing** group, waveforms at each electrode depicting sex differences in median absolute deviations of EEG amplitudes across trials between 1 ms (left of each channel subplot) and 350 ms (right of each channel subplot). MAD values on the Y-axis of each subplot range from 2.5 to 25.0. **Female** participants are marked by **red lines**, while **male** participants are marked by **blue lines**. |
| --- |

| 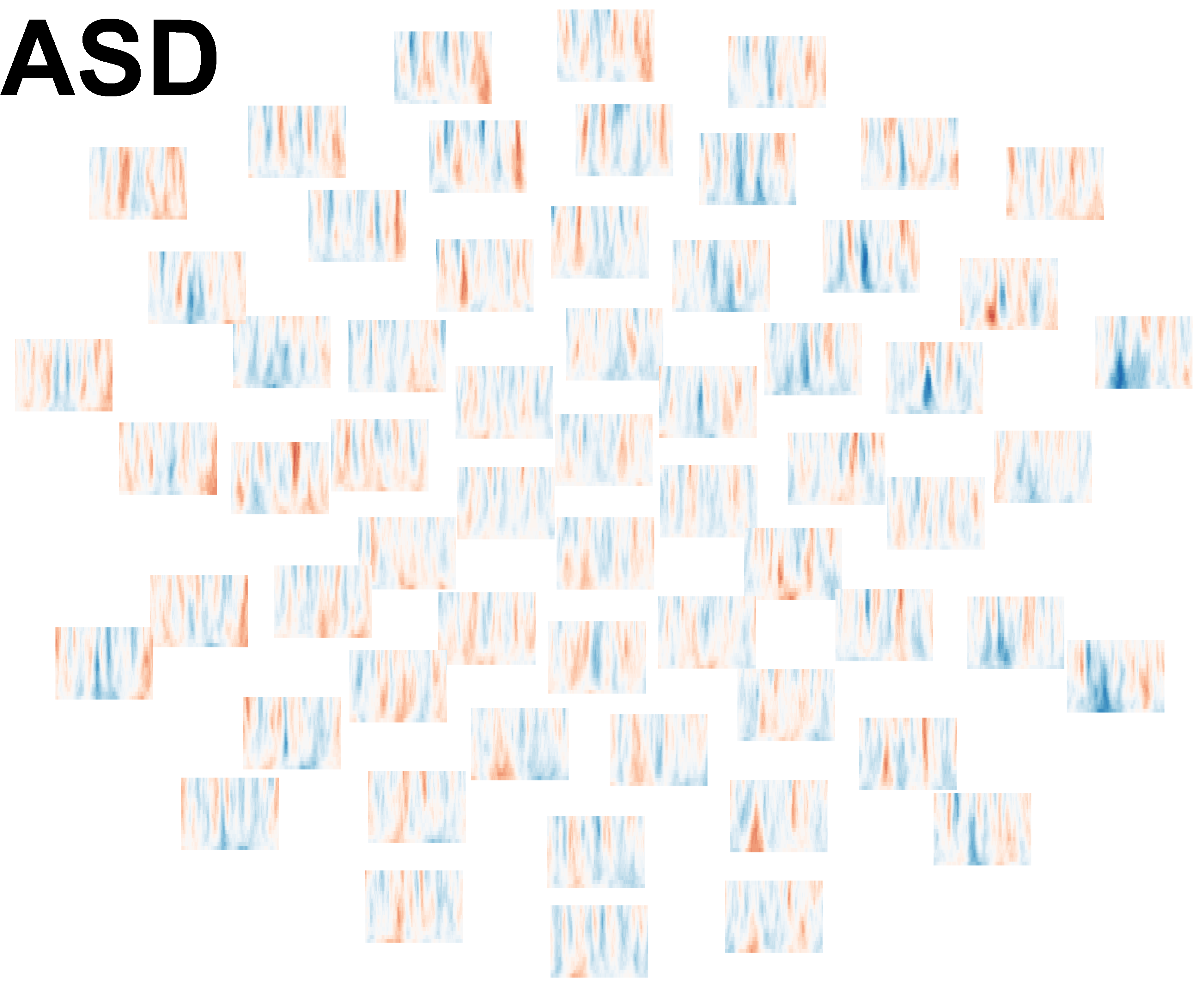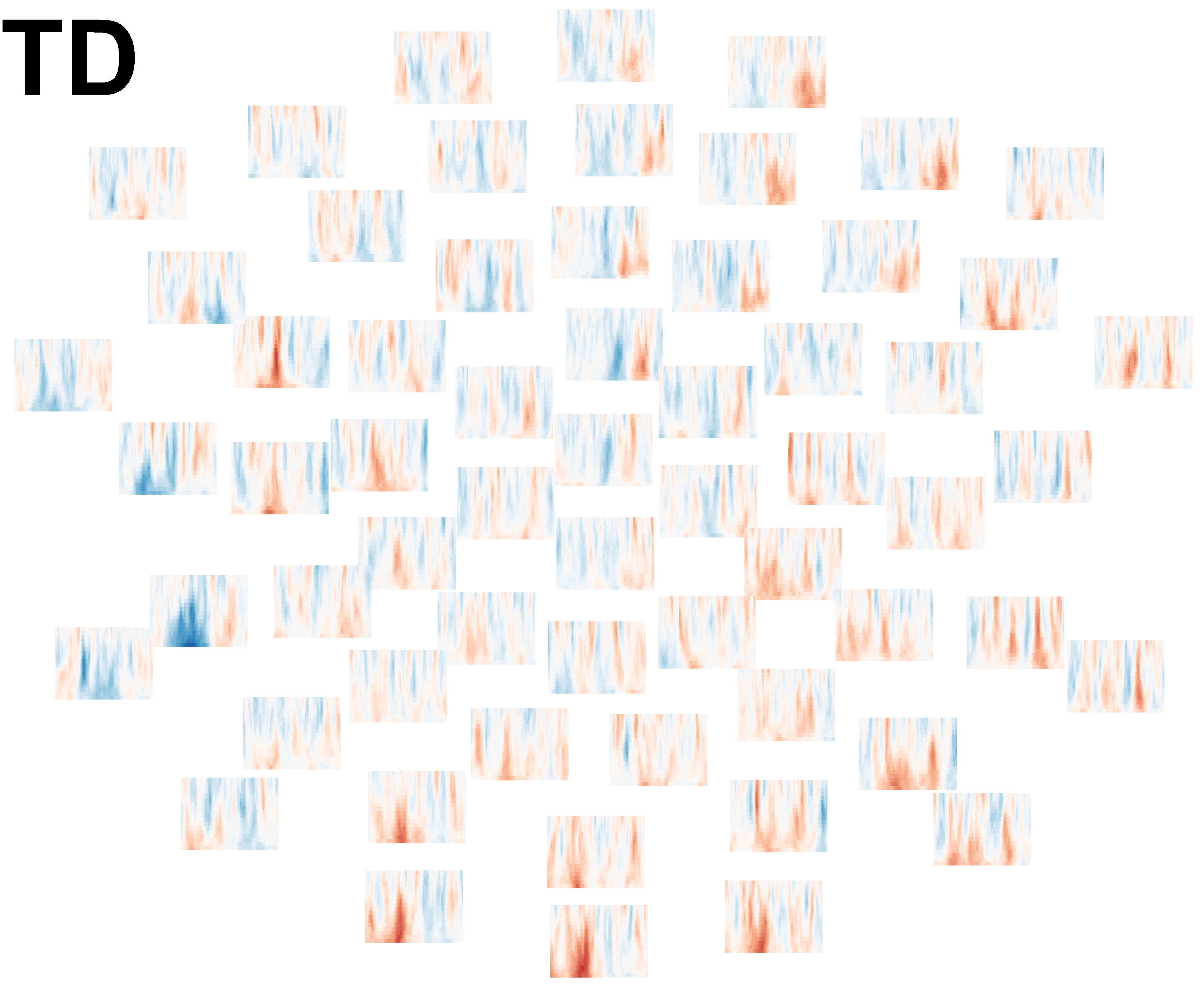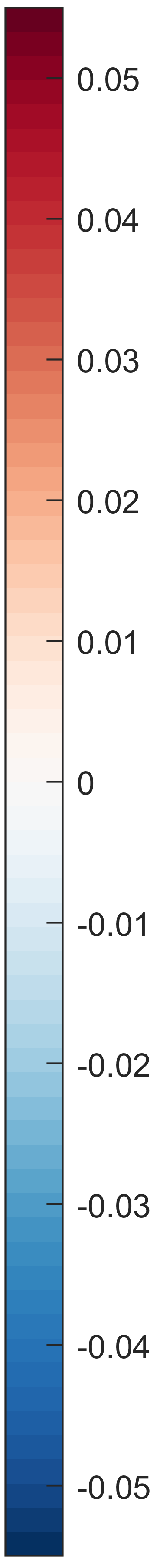  *Supplementary Figure 12*. In the **50 dB condition,** spectral plots at each electrode depicting sex differences in ITPC (female – male) across trials between 1 ms (left of each channel subplot) and 350 ms (right of each channel subplot). ITPC difference values on the Y-axis of each subplot range from – .055 to +.055. Higher ITPC in female participants is reflected by **positive (red) values**, while higher ITPC in male participants is marked by **negative (blue) values**. |
| --- |

| 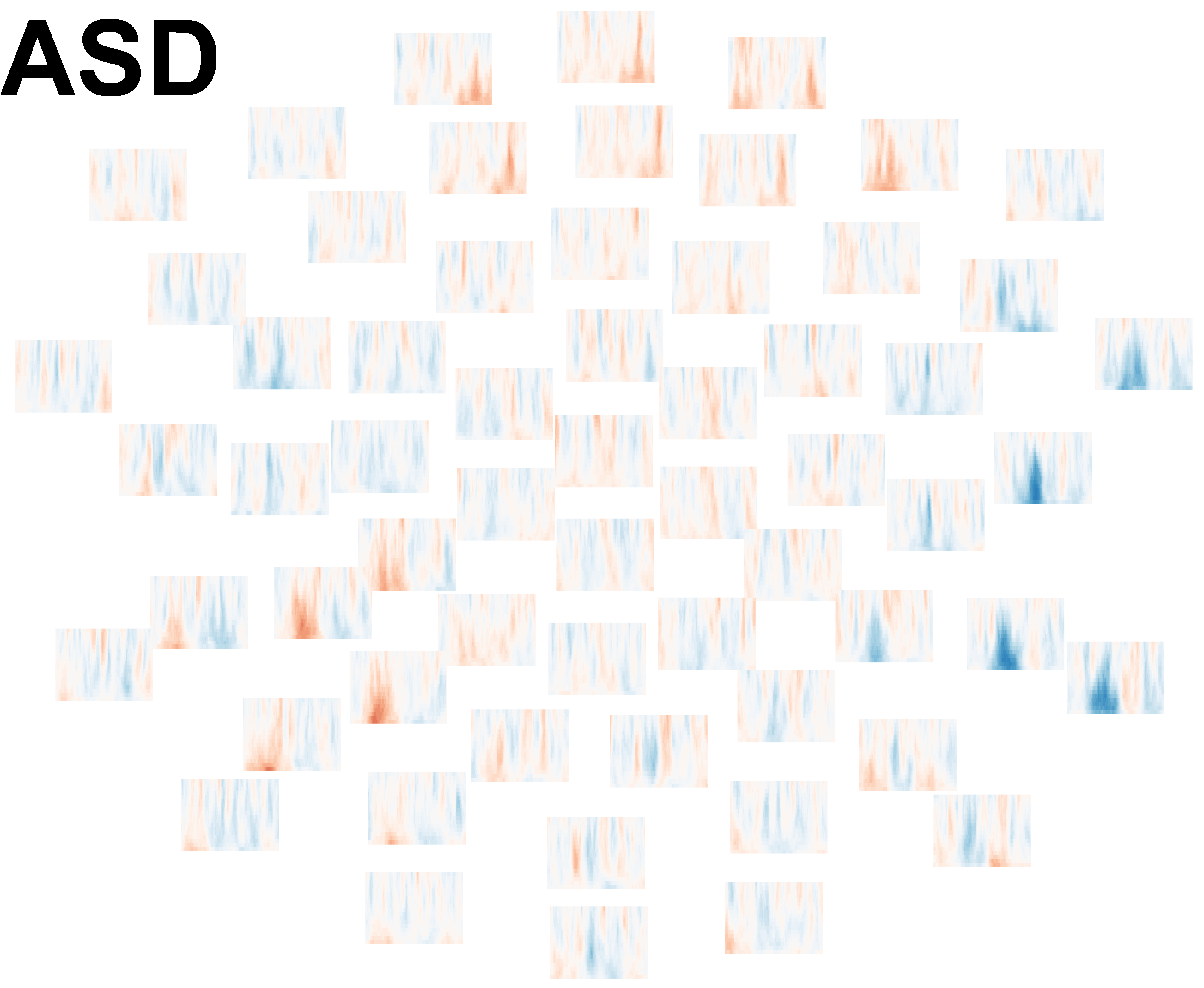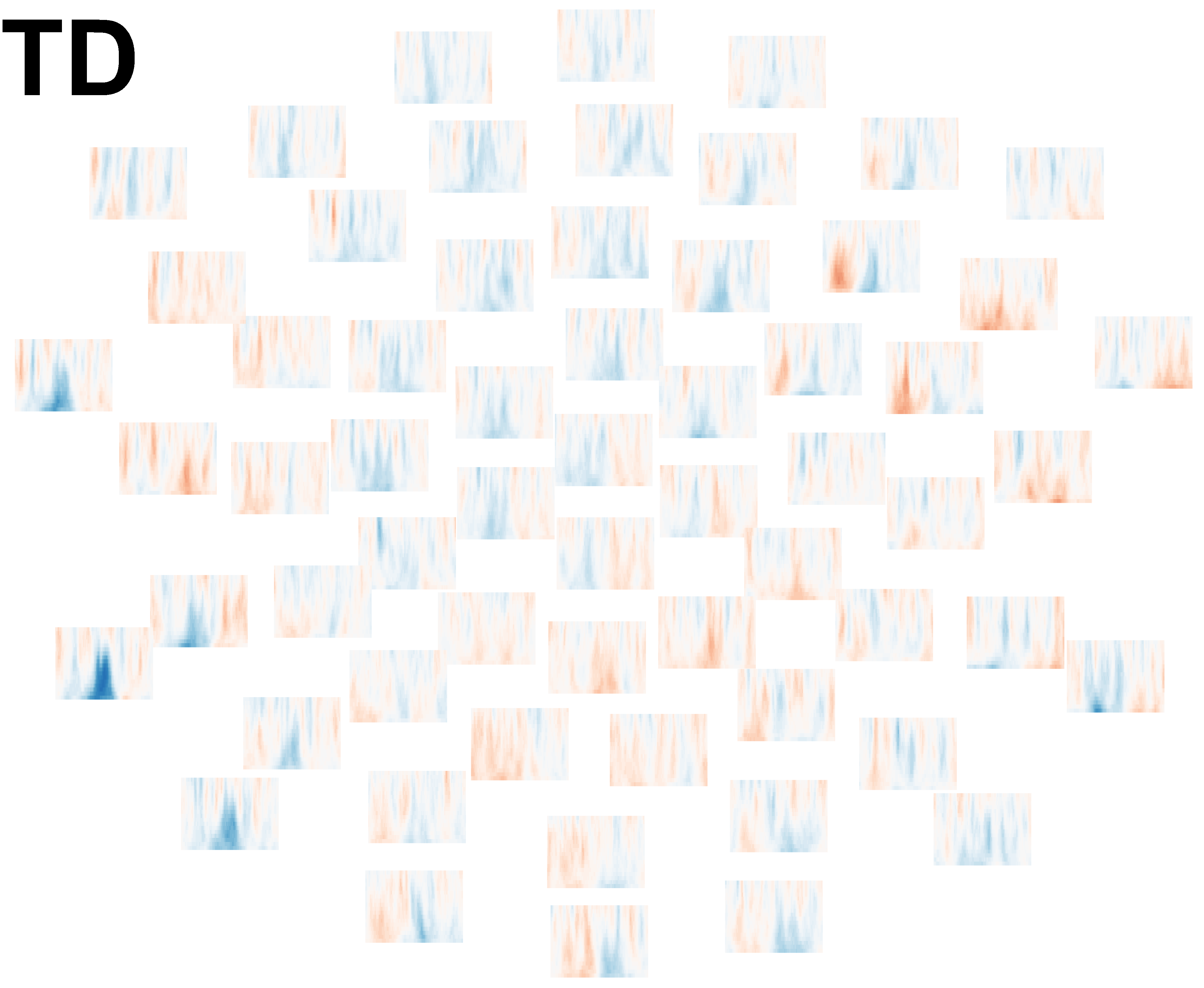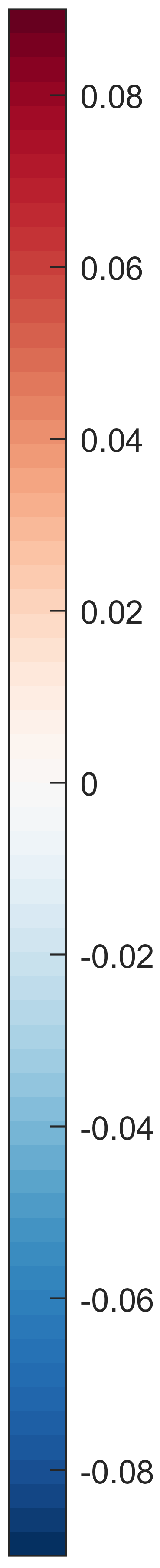  *Supplementary Figure 13*. In the **60 dB condition,** spectral plots at each electrode depicting sex differences in ITPC (female – male) across trials between 1 ms (left of each channel subplot) and 350 ms (right of each channel subplot). ITPC difference values on the Y-axis of each subplot range from – .09 to +.09. Higher ITPC in female participants is reflected by **positive (red) values**, while higher ITPC in male participants is marked by **negative (blue) values**. |
| --- |

| 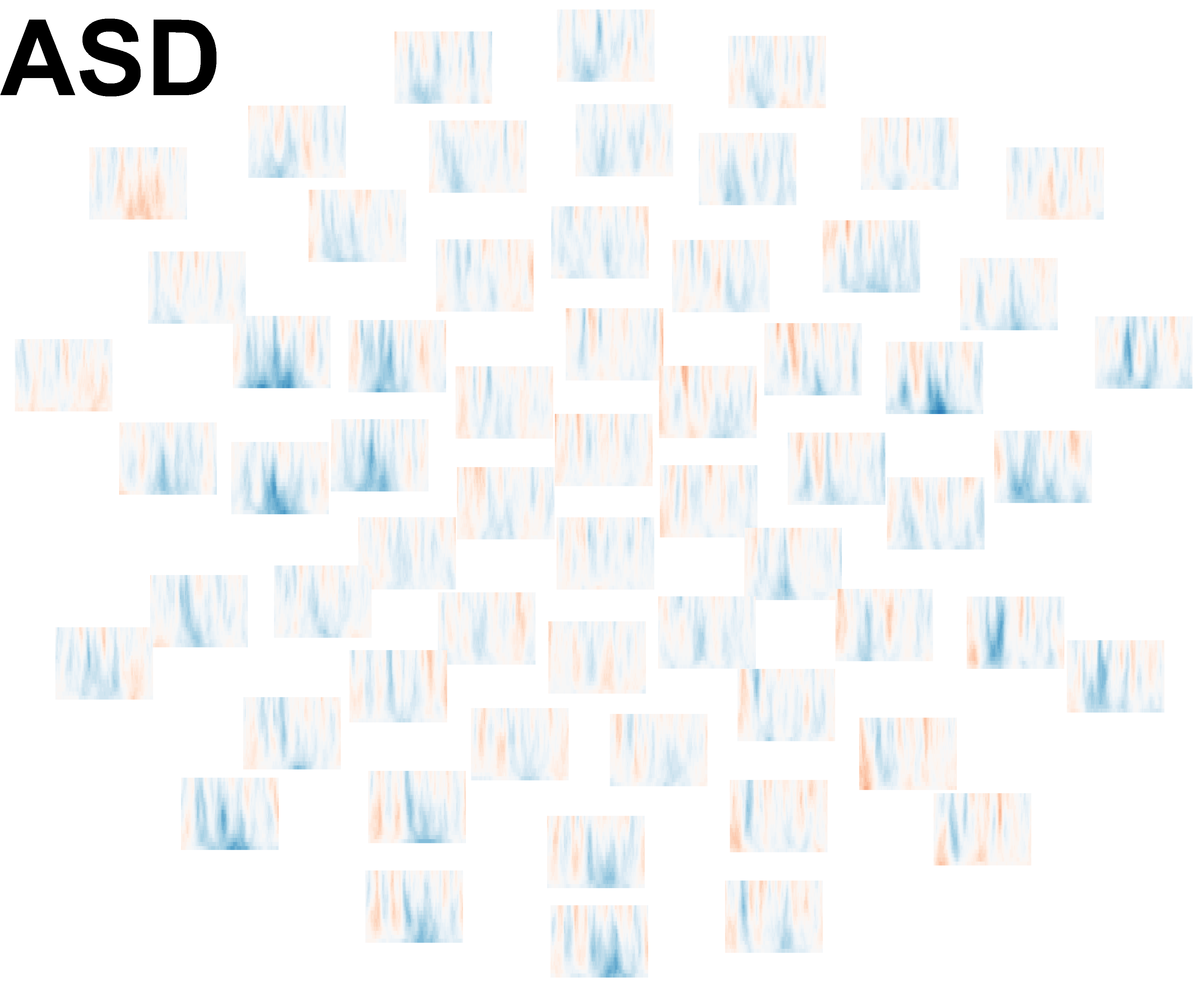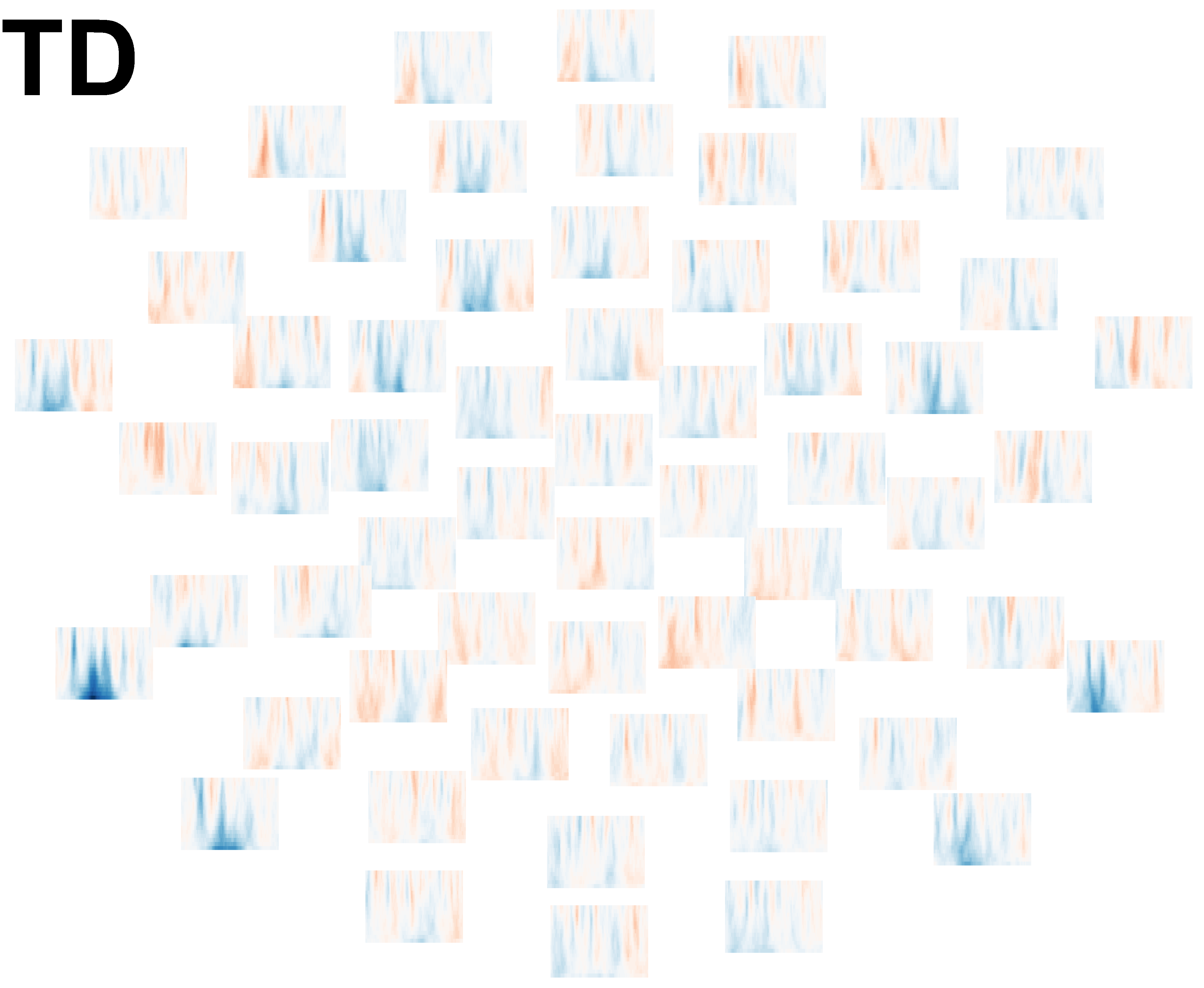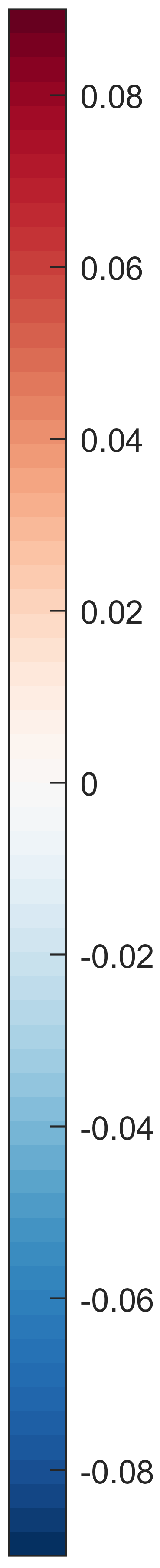  *Supplementary Figure 14*. In the **70 dB condition,** spectral plots at each electrode depicting sex differences in ITPC (female – male) across trials between 1 ms (left of each channel subplot) and 350 ms (right of each channel subplot). ITPC difference values on the Y-axis of each subplot range from – .09 to +.09. Higher ITPC in female participants is reflected by **positive (red) values**, while higher ITPC in male participants is marked by **negative (blue) values**. |
| --- |

| 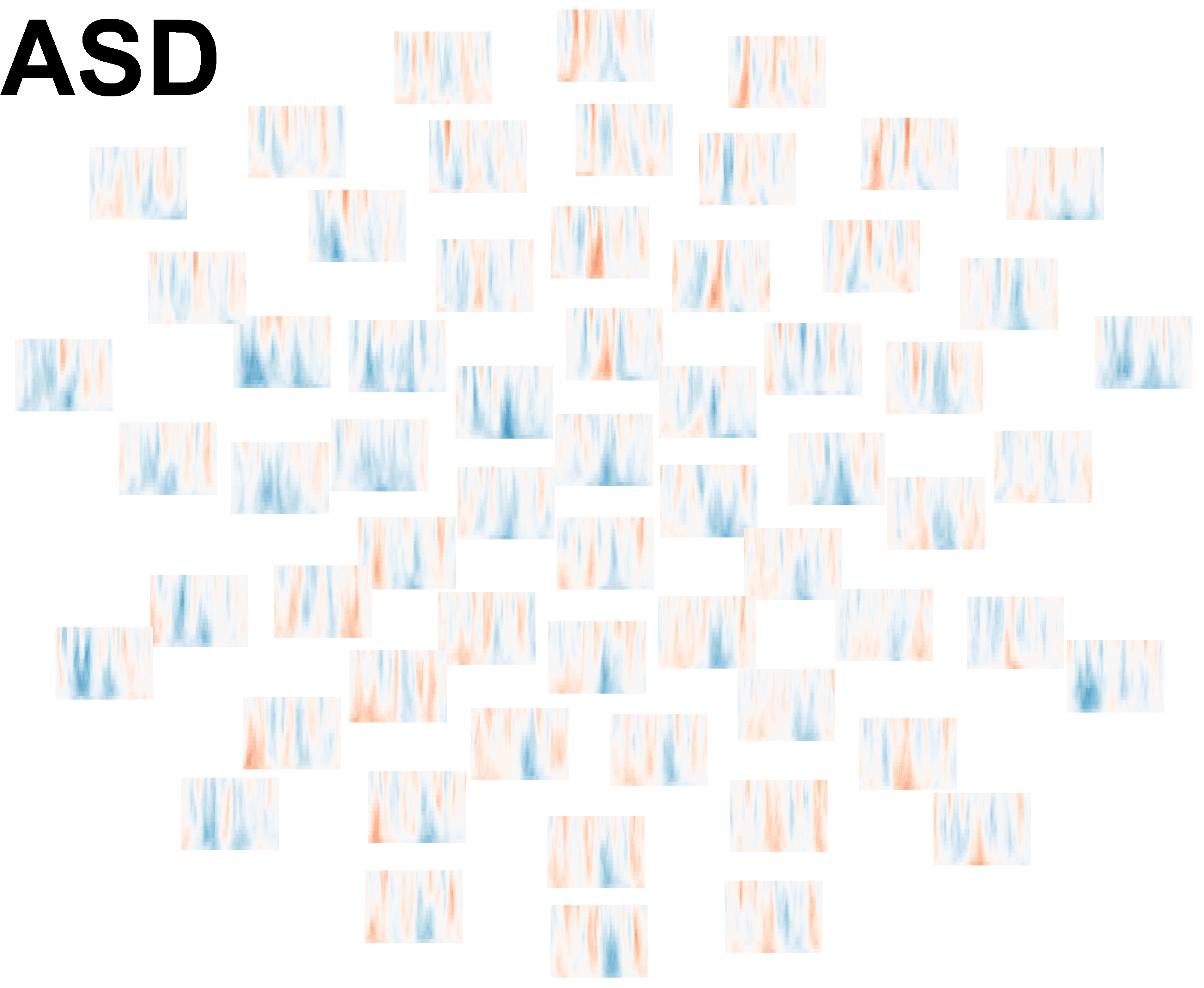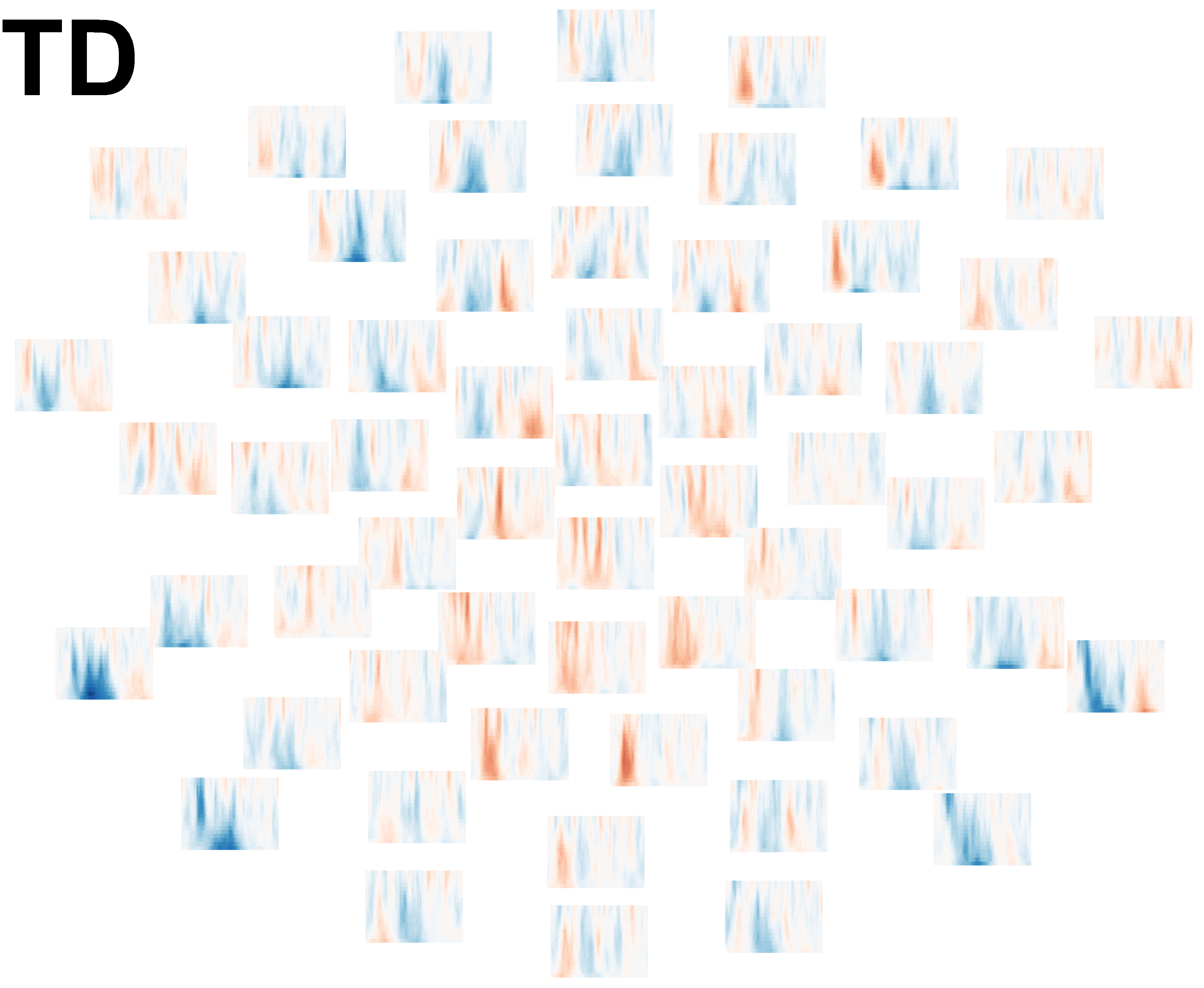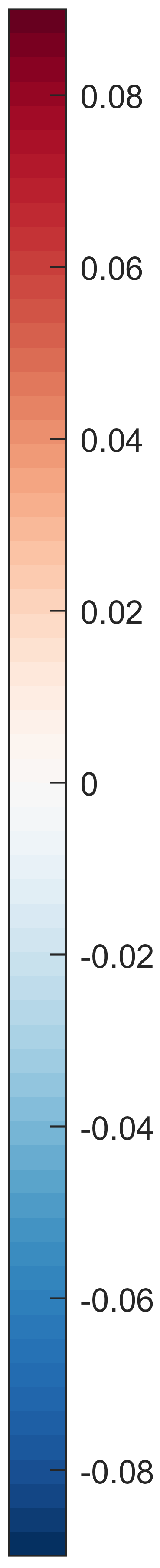  *Supplementary Figure 15*. In the **80 dB condition,** spectral plots at each electrode depicting sex differences in ITPC (female – male) across trials between 1 ms (left of each channel subplot) and 350 ms (right of each channel subplot). ITPC difference values on the Y-axis of each subplot range from – .09 to +.09. Higher ITPC in female participants is reflected by **positive (red) values**, while higher ITPC in male participants is marked by **negative (blue) values**. |
| --- |

# Appendix C. Correlations with Loudness Discomfort in Typically-Developing Participants

In addition to conducting hypothesis-driven analyses exploring correlations between loudness discomfort and inter-trial variability metrics in autistic participants, in the main text, we also carried out a supplementary and exploratory analysis here examining similar correlations in typically-developing participants.

## Median Absolute Deviations (MADs)

In the typically-developing group, we used cluster-based permutation tests (Maris & Oostenveld, 2007) to explore Spearman’s ρ (rho) correlations between SPHI loudness discomfort scores and MADs in all channels and all time points between 1 and 350 ms, separately in each intensity condition. The method used was identical to that used in the autistic group and presented in the main text.

No statistically significant associations were observed between SPHI scores and MADs in typically-developing participants in any intensity condition: not in the 50 dB condition, *p*≥.49, the 60 dB condition, *p*≥.43, the 70 dB condition, *p*≥.39, or the 80 dB condition, *p*≥.44 (*Supplementary Figure 16*).

## Inter-Trial Phase Coherence (ITPC)

In the typically-developing group, we used cluster-based permutation tests (Maris & Oostenveld, 2007) to explore Spearman’s ρ (rho) correlations between SPHI loudness discomfort scores and ITPC in all channels, all frequencies between 6 and 40 Hz, and all time points between 1 and 350 ms, separately in each intensity condition. The method used was identical to that used in the autistic group and presented in the main text.

No statistically significant associations were observed between SPHI scores and ITPC in typically-developing participants in any intensity condition: not in the 50 dB condition, *p*≥.41 (*Supplementary Figure 17*), the 60 dB condition, *p*>.99 (*Supplementary Figure 18*), the 70 dB condition, *p*≥.31 (*Supplementary Figure 19*), or the 80 dB condition, *p*≥.85 (*Supplementary Figure 20*).

| 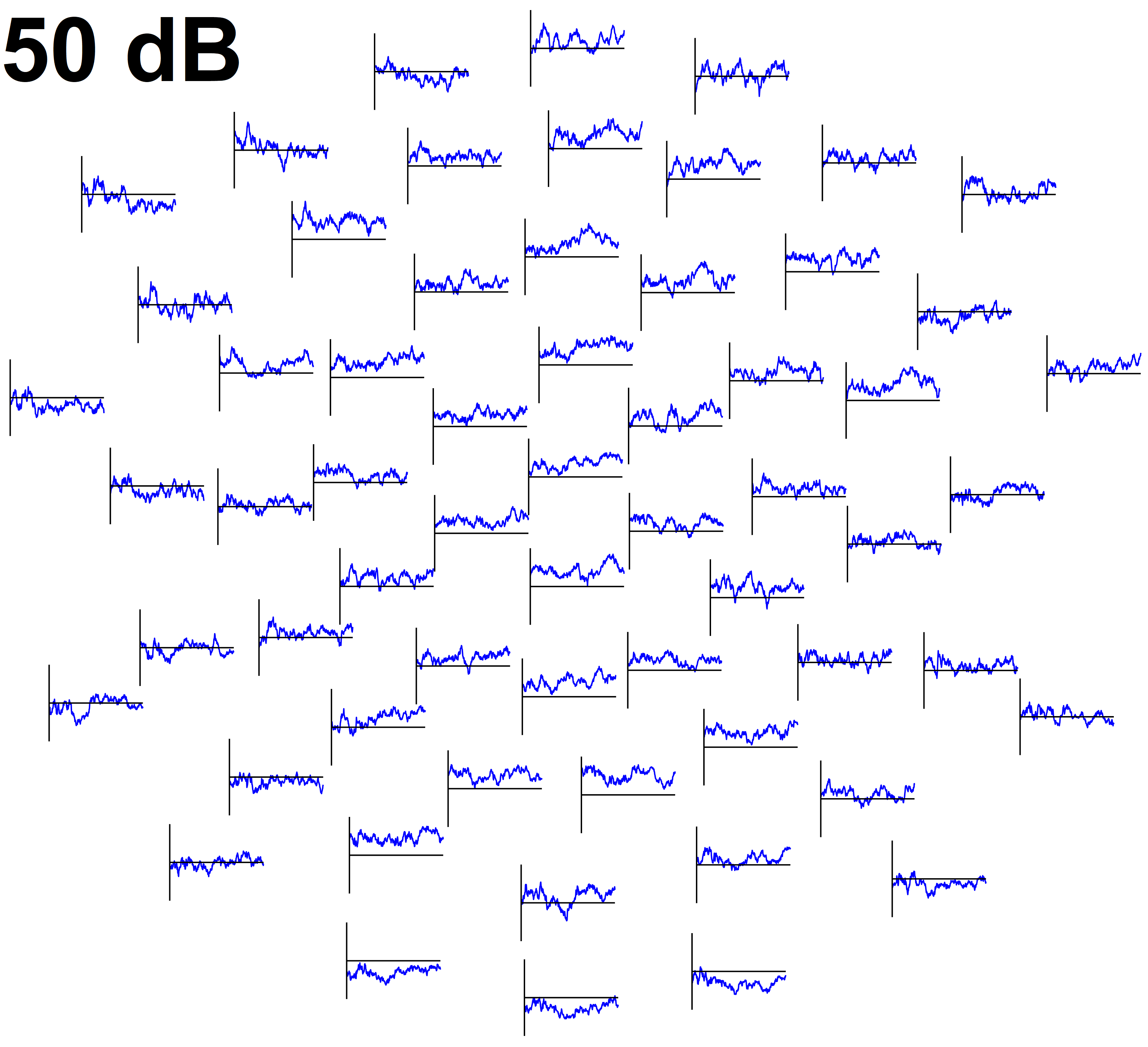 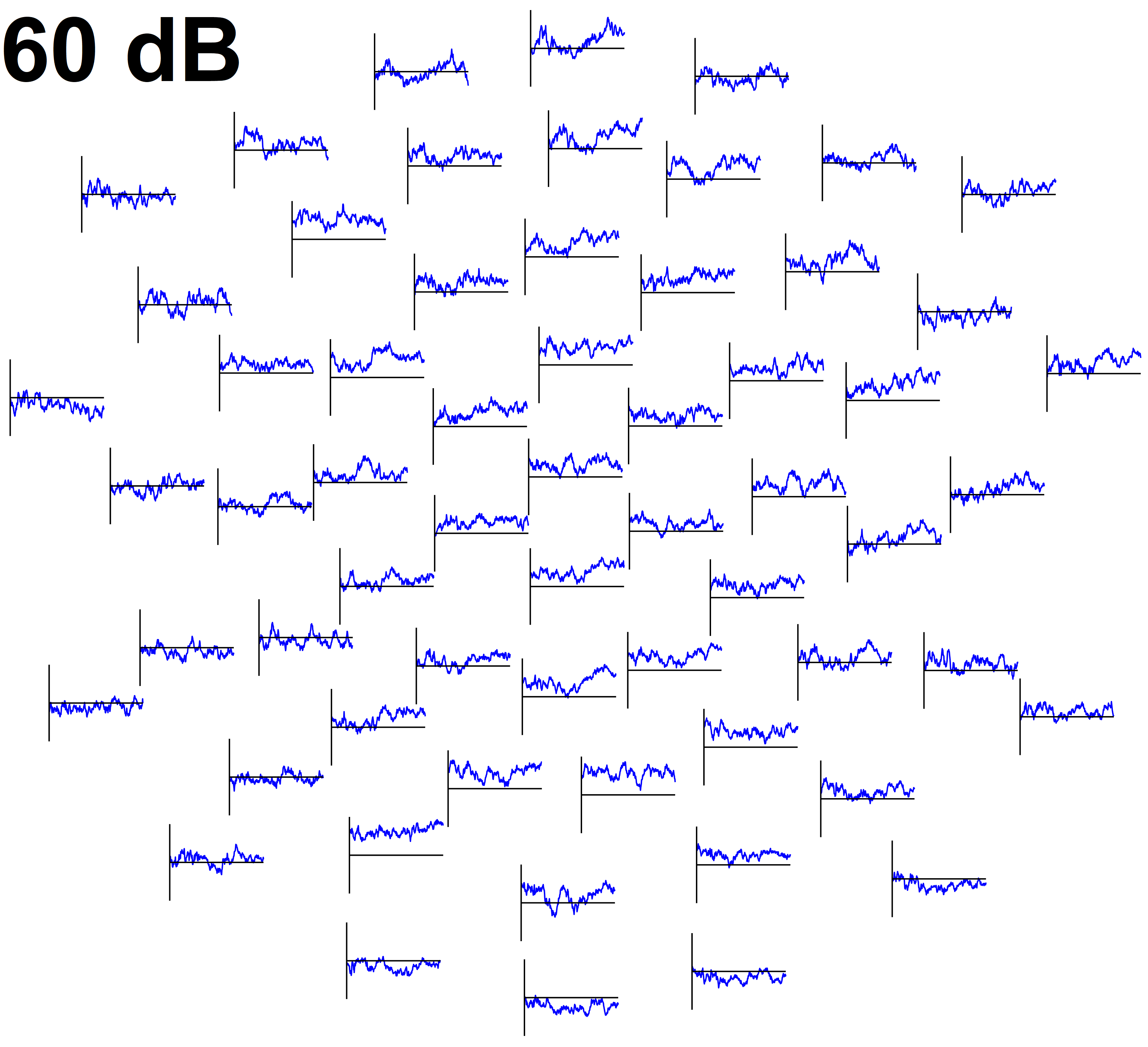  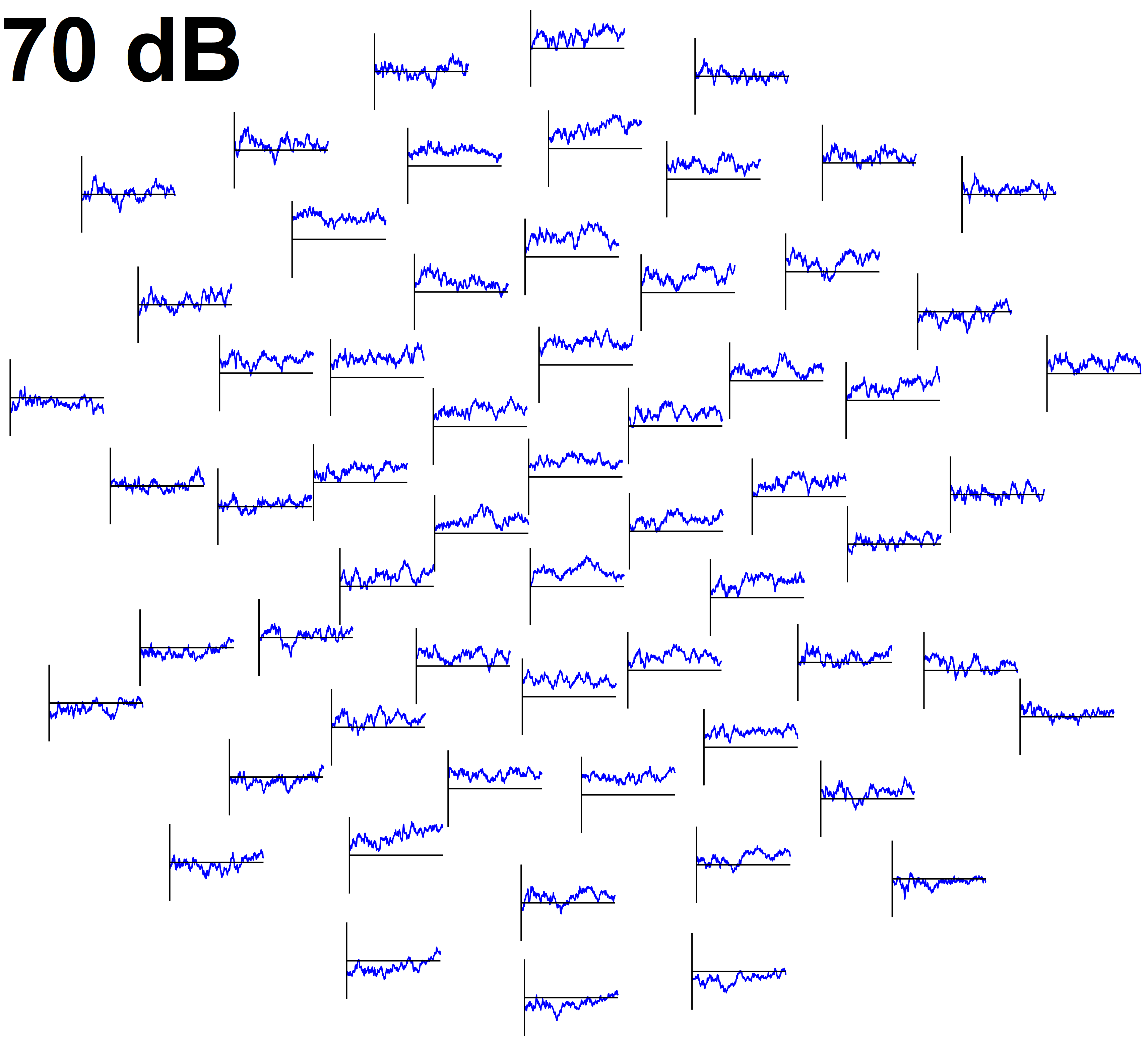 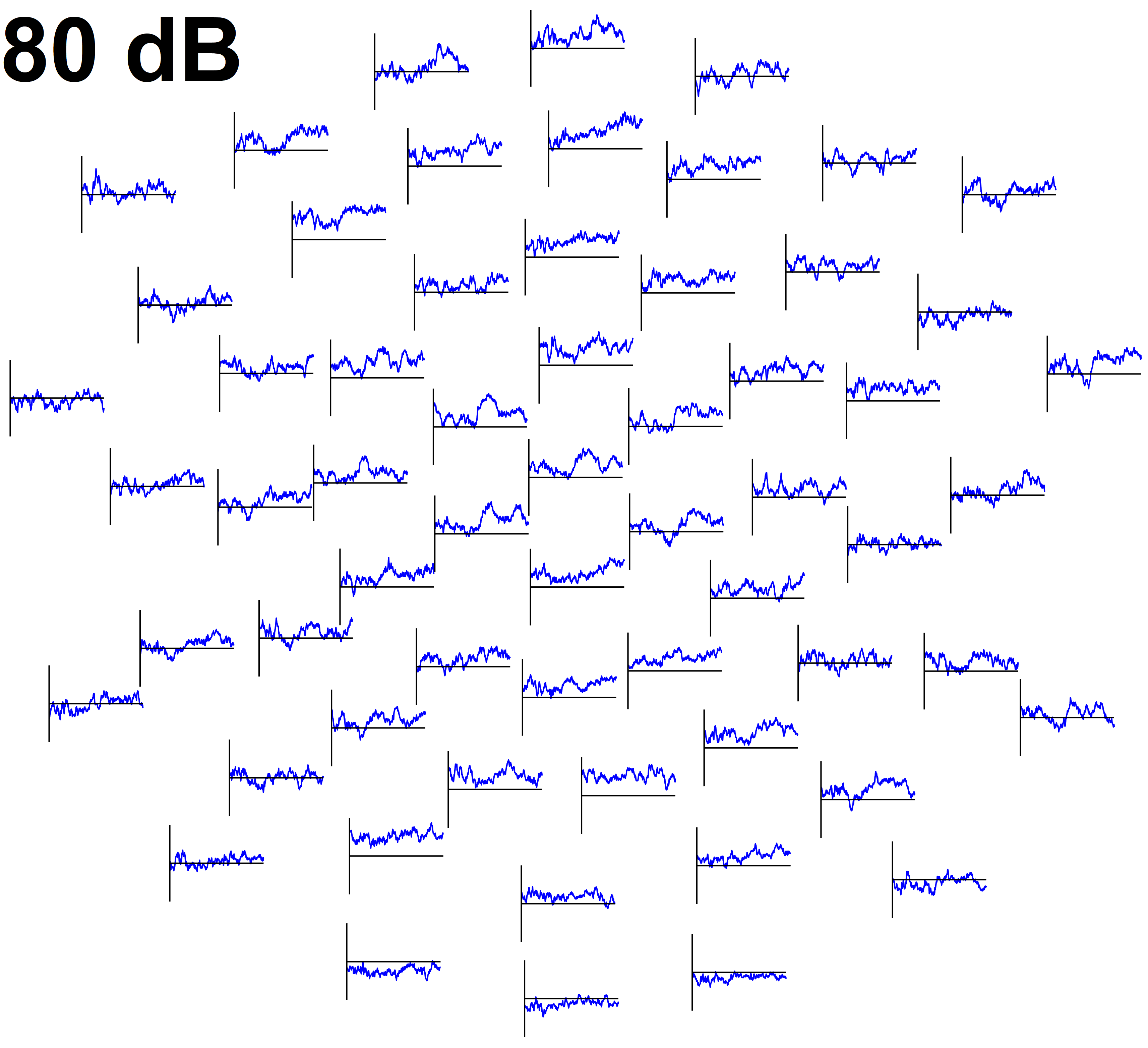  *Supplementary Figure 16*. In the typically-developing group, waveforms at each electrode depicting Spearman’s ρ ordinal correlation coefficients between loudness discomfort and median absolute deviations of EEG amplitudes across trials between 1 ms (left of each channel subplot) and 350 ms (right of each channel subplot). The Y-axis, representing correlation coefficients, ranges from −0.38 (bottom) to +0.38 (top). There were no significant correlations in any intensity condition. |
| --- |

| 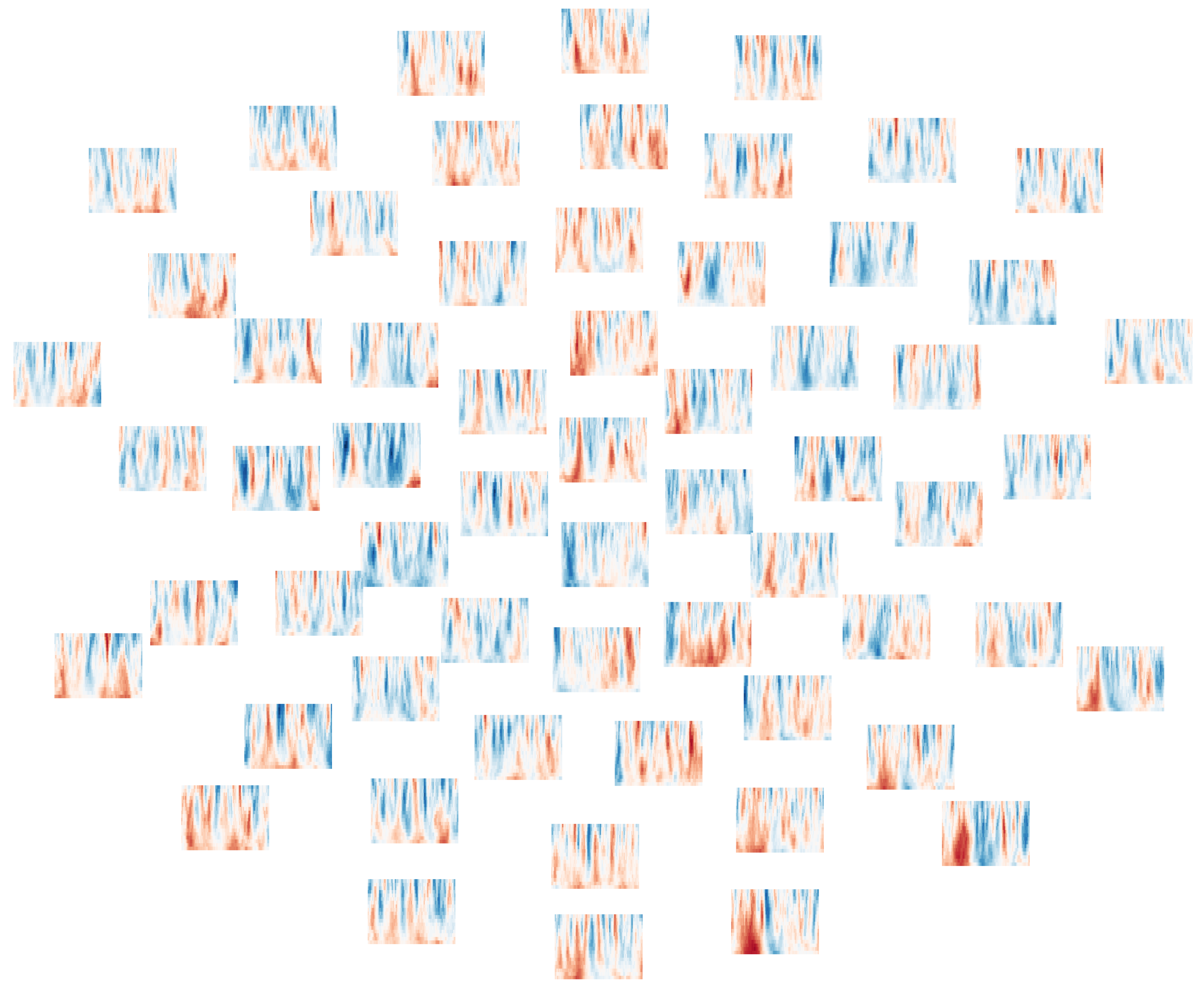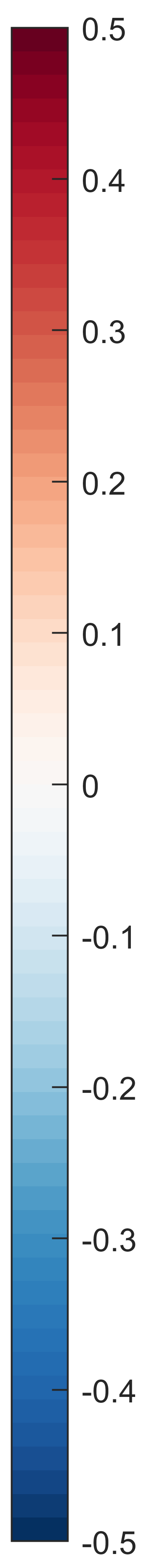*Supplementary Figure 17*. In the typically-developing group and in the 50 dB condition, spectral plots at each electrode depicting Spearman’s ρ ordinal correlation coefficients between loudness discomfort and ITPC of EEG responses in each frequency (40 Hz at top; 6 Hz at bottom of each subplot) between 1 ms (left of each channel subplot) and 350 ms (right of each channel subplot). There were no significant correlations. |
| --- |

| 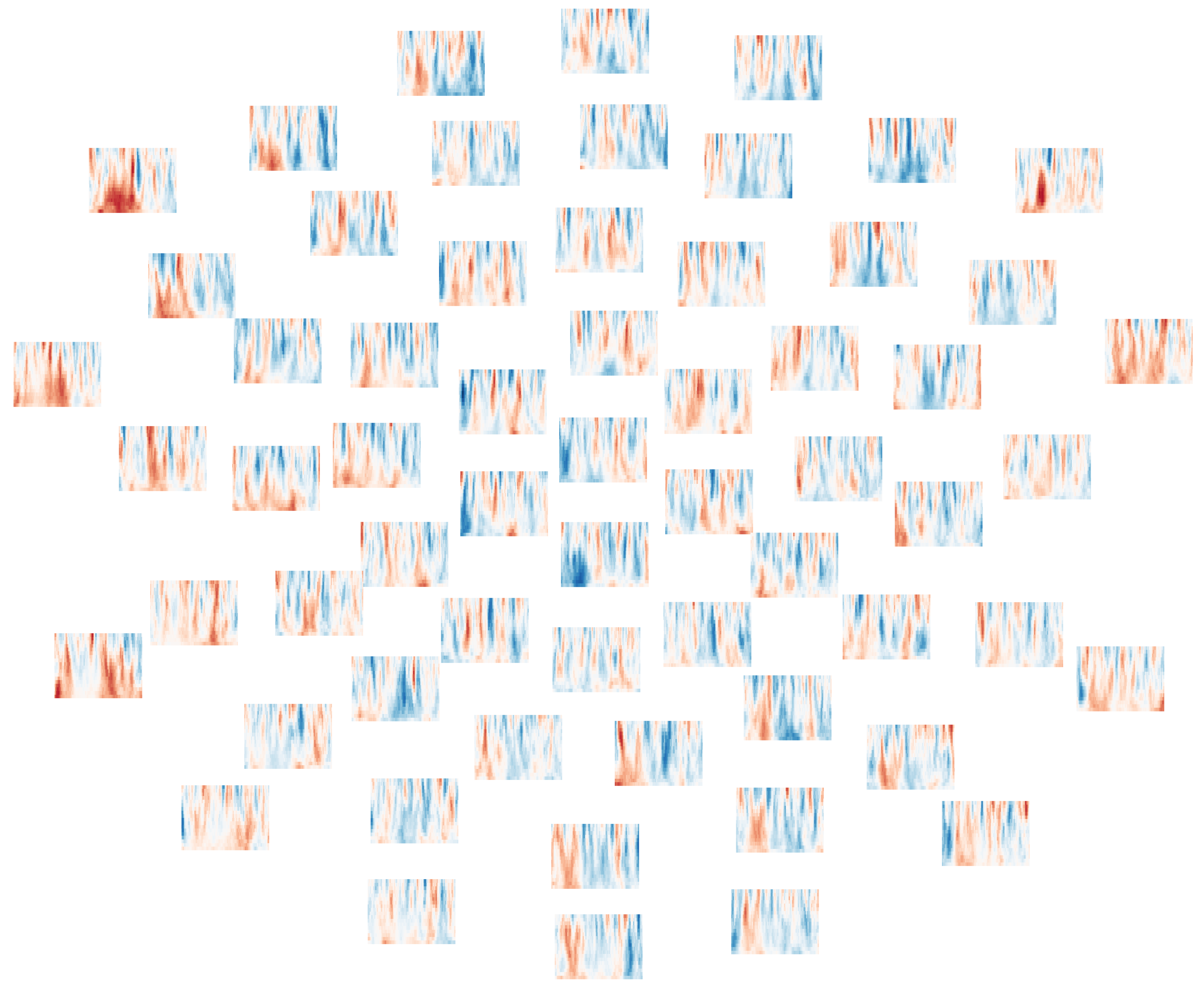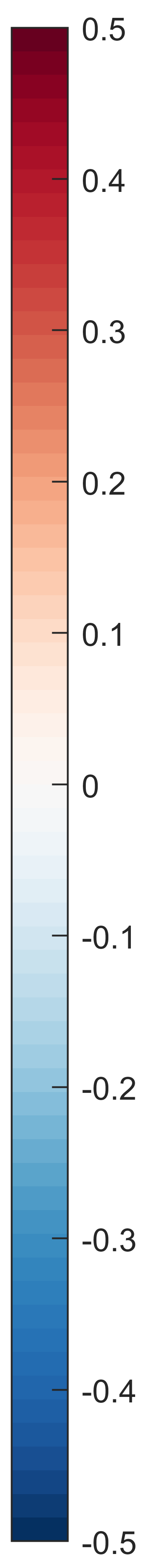*Supplementary Figure 18*. In the typically-developing group and in the 60 dB condition, spectral plots at each electrode depicting Spearman’s ρ ordinal correlation coefficients between loudness discomfort and ITPC of EEG responses in each frequency (40 Hz at top; 6 Hz at bottom of each subplot) between 1 ms (left of each channel subplot) and 350 ms (right of each channel subplot). There were no significant correlations. |
| --- |

| 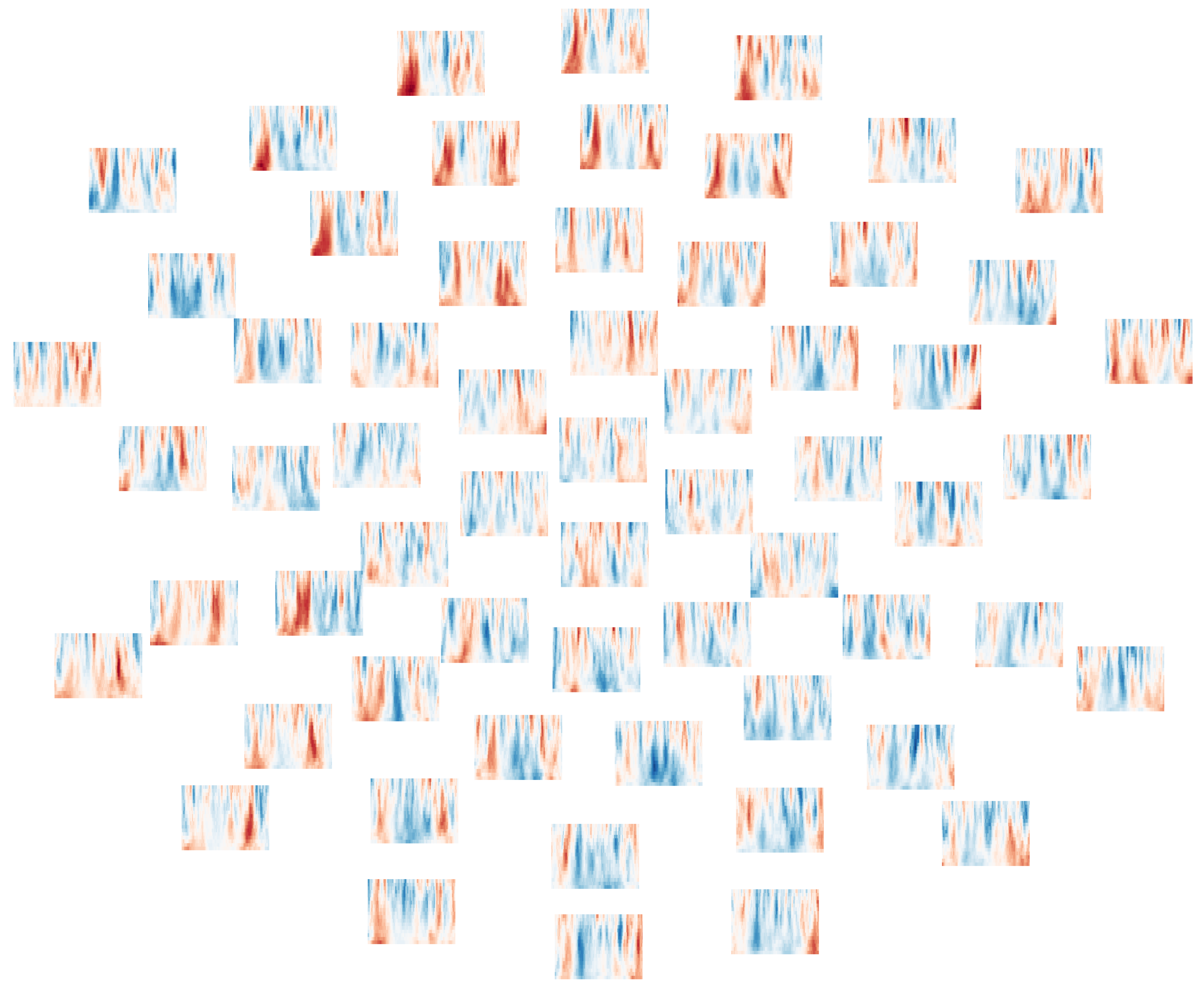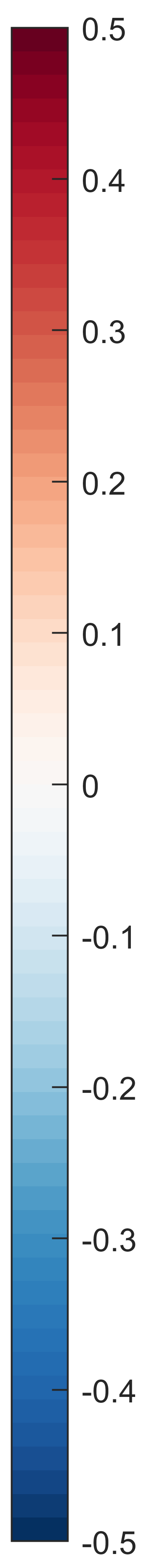*Supplementary Figure 19*. In the typically-developing group and in the 70 dB condition, spectral plots at each electrode depicting Spearman’s ρ ordinal correlation coefficients between loudness discomfort and ITPC of EEG responses in each frequency (40 Hz at top; 6 Hz at bottom of each subplot) between 1 ms (left of each channel subplot) and 350 ms (right of each channel subplot). There were no significant correlations. |
| --- |

| 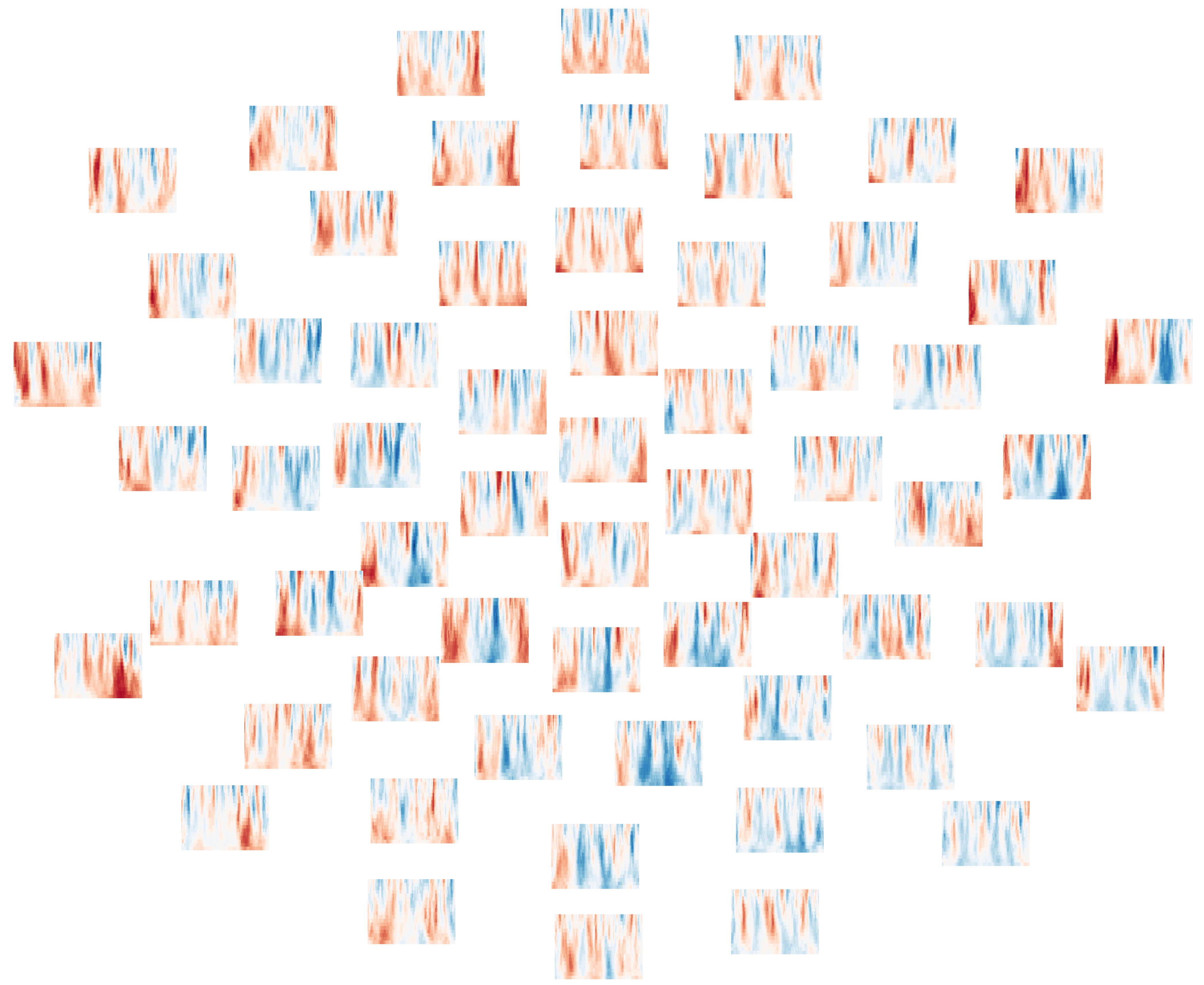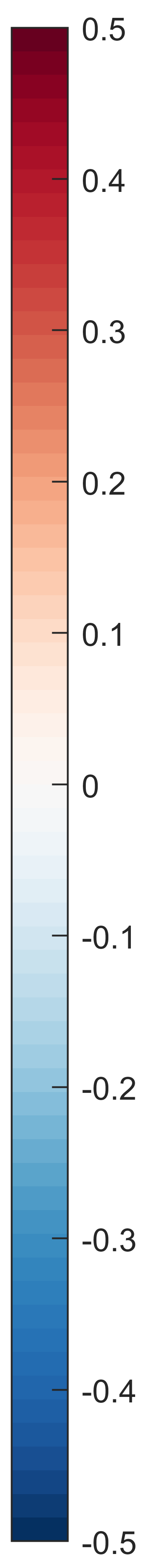*Supplementary Figure 20*. In the typically-developing group and in the 80 dB condition, spectral plots at each electrode depicting Spearman’s ρ ordinal correlation coefficients between loudness discomfort and ITPC of EEG responses in each frequency (40 Hz at top; 6 Hz at bottom of each subplot) between 1 ms (left of each channel subplot) and 350 ms (right of each channel subplot). There were no significant correlations. |
| --- |

# Appendix D. Bayesian Analysis of Median Absolute Deviations

As our failure to observe significant effects of diagnostic group on median absolute deviations from the main text does not prove such effects are absent, we conducted a supplementary Bayesian regression analysis to further examine whether there were meaningful effects of diagnostic group on median absolute deviations of EEG amplitudes across trials.

We also aimed to use this analysis to investigate further the possibility of a diagnosis*sex interaction, as supplementary analyses reported above found trending effects of sex in autistic participants over posterior channels in the 50 dB condition.

Unlike the main text analysis, which uses cluster-based permutation tests to examine many median absolute deviation values across separate time-points and channels, the Bayesian analysis was conducted using single median absolute deviation averaged across spatiotemporal windows defined by the P1 and N2 event-related potentials (ERPs) from this dataset.

For the selection of these spatiotemporal regions of interest, ERPs were extracted from the EEG data. The P1 and N2 spatial windows were defined based on visual inspection of the ERP topography (*Supplementary Figure 25*); each window covered a region of left and right hemisphere frontocentral channels. P1 time windows were defined as +/-50 ms around the P1 peak, which yielded the following time windows: 73 – 173 ms (50 dB), 61 – 161 ms (60 dB), 45 – 145 ms (70 dB), and 43 – 143 ms (80 dB). As the N2 response lacked clearly defined peaks in softer intensity conditions such as 50 and 60 dB, we utilized a single 201 – 350 ms time window for measuring the frontocentral N2.

For the Bayesian analysis, median absolute deviations of EEG responses across trials were averaged across channels and time-points within these P1 and N2 ERP-based regions of interest.

It is important to note here that the median absolute deviations (as, e.g., depicted in *Figures 1-2* from the main text) show no clear modulation by event-related responses. Instead, they increase relatively steadily from the middle of the baseline period (where amplitude variability between trials is limited due to baseline subtraction) until the end of the epoch. Therefore, any systematic differences in neural variability they reflect may be endogenous, with relatively little stimulus-related activity. Thus, the decision to constrain the MAD analysis to spatiotemporal windows defined by ERPs is fairly arbitrary; for the Bayesian analysis to be computationally feasible, we required single averaged values rather than many separate multivariate data points. The ERP-based spatiotemporal windows provided a convenient set of *post hoc* windows for averaging of responses for this analysis, but we do not believe that the median absolute deviations of EEG amplitudes across trials in the P1 and N2 spatiotemporal windows should be taken to represent the variability of the P1 or N2 event-related responses themselves.

The Bayesian analysis was conducted using a hierarchical student-t regression model fit using the *brms* package in R (Bürkner, 2017). The analysis examined whether effects were likely to be *practically significant* (Kirk, 1996), as defined by an absolute value of Cohen’s *d* of 0.2 or larger, representing a “small” effect size: that is, in this analysis, the null hypothesis was that effects would lie within a “region of practical equivalence” (ROPE) defined as *d* = [-0.2, 0.2], while the alternative hypothesis was that effect sizes would have a larger absolute value of *d*. ROPE-based Bayes factors (*BF*_ROPE_) greater than 3 can be interpreted as substantial support for the alternative hypothesis, while values less than 1/3 can be interpreted as substantial support for the null hypothesis; values between 1/3 and 3 can be interpreted as inconclusive (Wagenmakers et al., 2011). 95% credible intervals (CrI_95%_) of values of *d* are also reported.

To transform data into a metric based on standard deviations (and thus equivalent to Cohen’s *d*), the mean MAD value (across all stimulus intensities) was subtracted from each MAD value, after which they were divided by the standard deviation of MAD values (across all stimulus intensities), generating standardized z-scores.

Fixed effects of diagnostic group and sex, and their interaction, were examined along with random intercepts by participant ID and stimulus intensity, as well as random slopes of diagnosis, sex, and diagnosis*sex by stimulus intensity level. Residual standard deviation was also allowed to vary by diagnosis, sex, diagnosis*sex, and stimulus intensity. Default brms priors were used, with the exception of a Normal(0,1) prior on fixed effect regression slopes.

**Results**

In the analysis of the P1 spatiotemporal window, the null hypothesis of no practically significant effects was supported for the effect of diagnostic group, CrI_95%_ = [–0.634, 0.453], *BF*_ROPE_ = 0.183, the effect of sex, CrI_95%_ = [–0.480, 0.447], *BF*_ROPE_ = 0.122, and the interaction of diagnosis and sex, CrI_95%_ = [–0.554, 0.664], *BF*_ROPE_ = 0.202.

Similarly, in the analysis of the N2 spatiotemporal window, the null hypothesis of no practically significant effects was supported for the effect of diagnosis, CrI_95%_ = [–0.604, 0.478], *BF*_ROPE_ = 0.172, the effect of sex, CrI_95%_ = [–0.364, 0.555], *BF*_ROPE_ = 0.134, and the interaction of diagnosis and sex, CrI_95%_ = [–0.619, 0.625], *BF*_ROPE_ = 0.207.

Overall, these results provide further support to the main text, suggesting that autistic participants do not exhibit greater trial-to-trial variability of EEG amplitudes than typically-developing participants.

These results also suggest that any trending sex differences in trial-to-trial variability of posterior EEG amplitudes do not influence variability of EEG amplitudes over the frontocentral channels associated with auditory responses (reflecting dipoles oriented tangentially relative to auditory cortex).

| 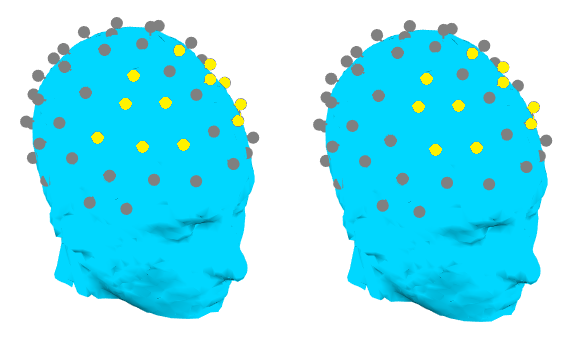 |
| --- |
| *Supplementary Figure 21*. Regions of interest over left and right hemisphere frontocentral scalp. *Left*. Spatiotemporal region of interest corresponding to the P1 ERP (yellow electrodes).  *Right*. Spatiotemporal region of interest corresponding to the N2 ERP (yellow electrodes). |

# Appendix E. Supplementary Median Absolute Deviation Figures

| 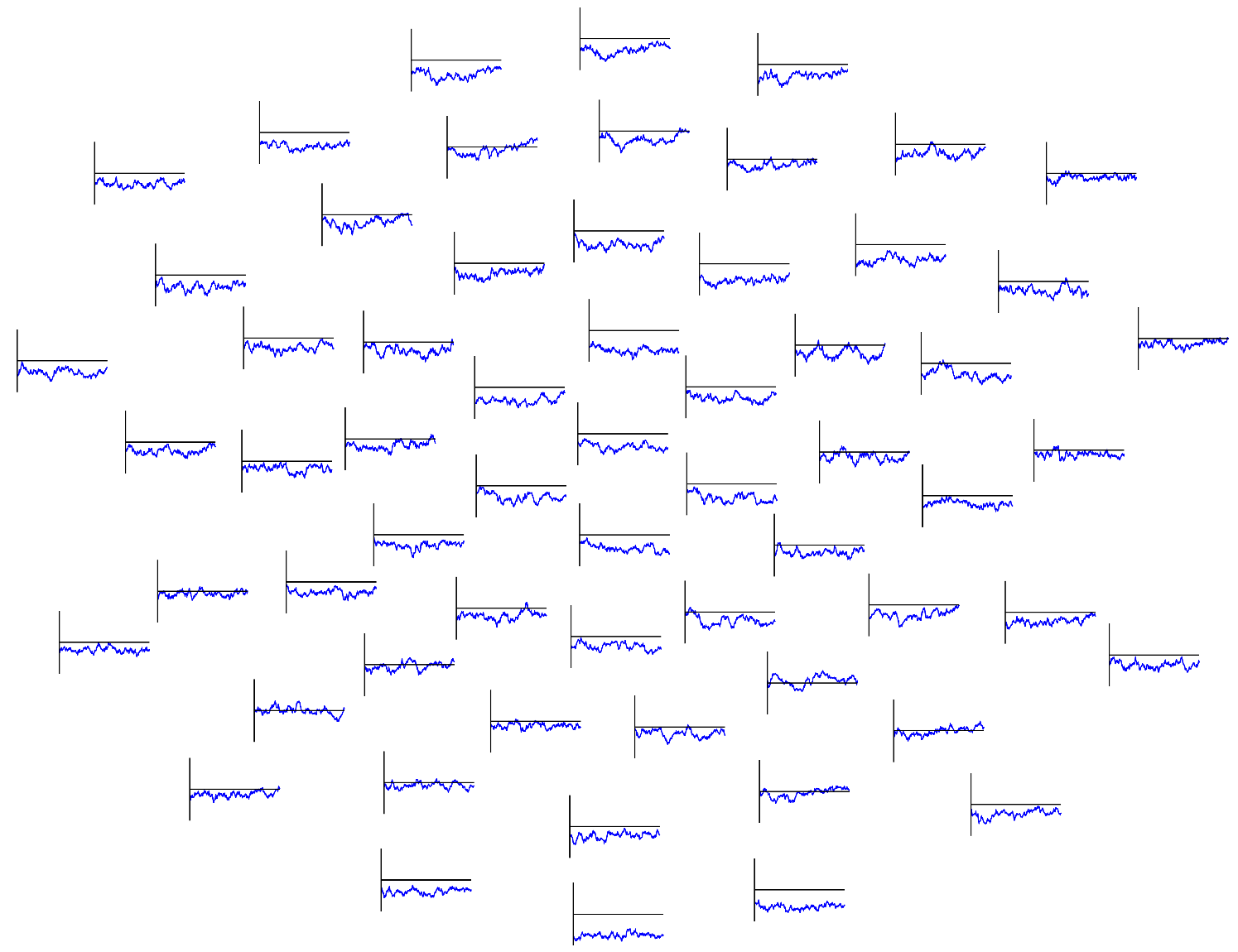 |
| --- |
| *Supplementary Figure 22*. In the autistic group and in the 50 dB condition, waveforms at each electrode depicting Spearman’s ρ ordinal correlation coefficients between loudness discomfort and median absolute deviations of EEG amplitudes across trials between 1 ms (left of each channel subplot) and 350 ms (right of each channel subplot). The Y-axis, representing correlation coefficients, ranges from −0.325 (bottom of each channel subplot) to +0.325 (top of each channel subplot). There were no significant correlations. |

| 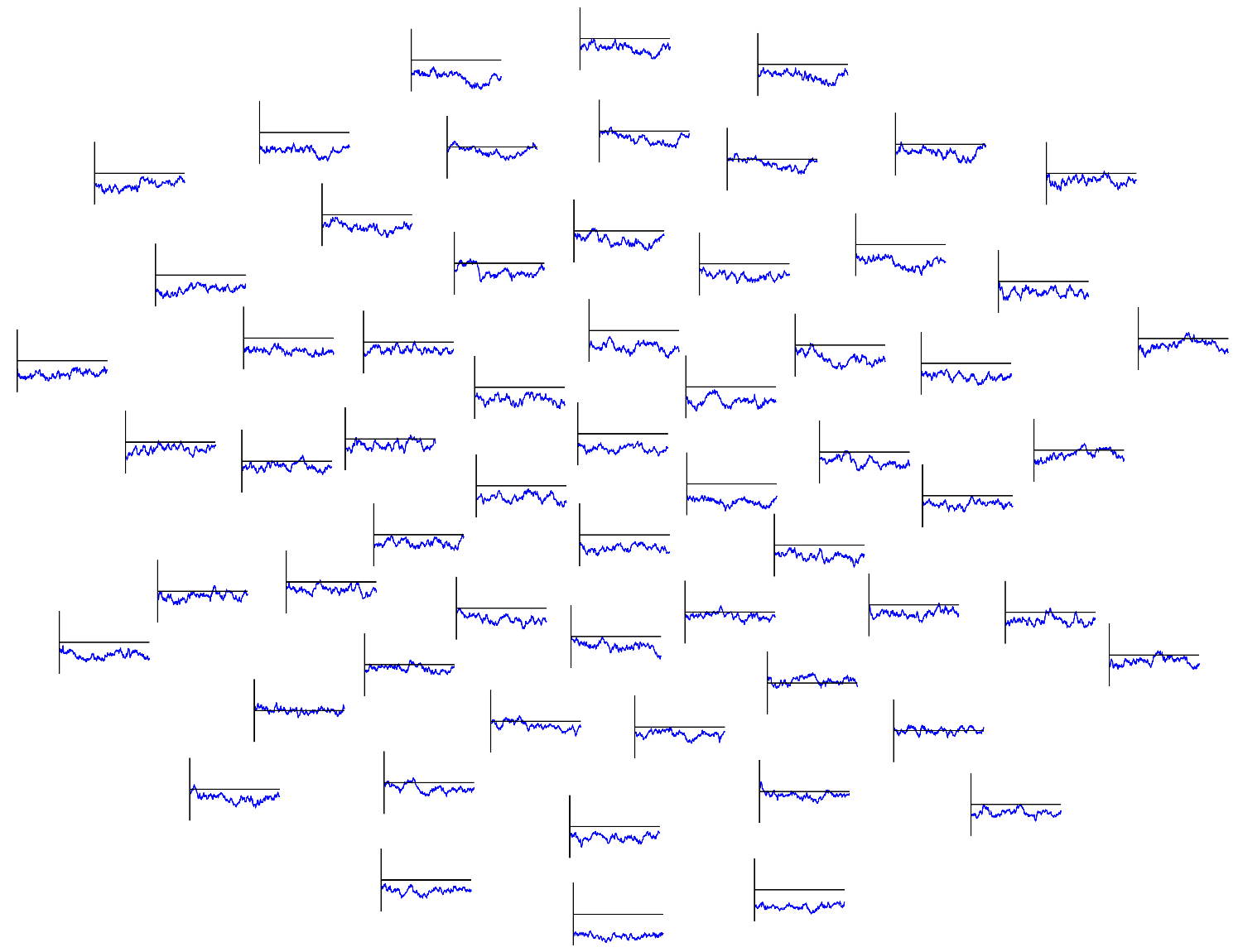 |
| --- |
| *Supplementary Figure 23*. In the autistic group and in the 60 dB condition, waveforms at each electrode depicting Spearman’s ρ ordinal correlation coefficients between loudness discomfort and median absolute deviations of EEG amplitudes across trials between 1 ms (left of each channel subplot) and 350 ms (right of each channel subplot). The Y-axis, representing correlation coefficients, ranges from −0.325 (bottom of each channel subplot) to +0.325 (top of each channel subplot). There were no significant correlations. |

| 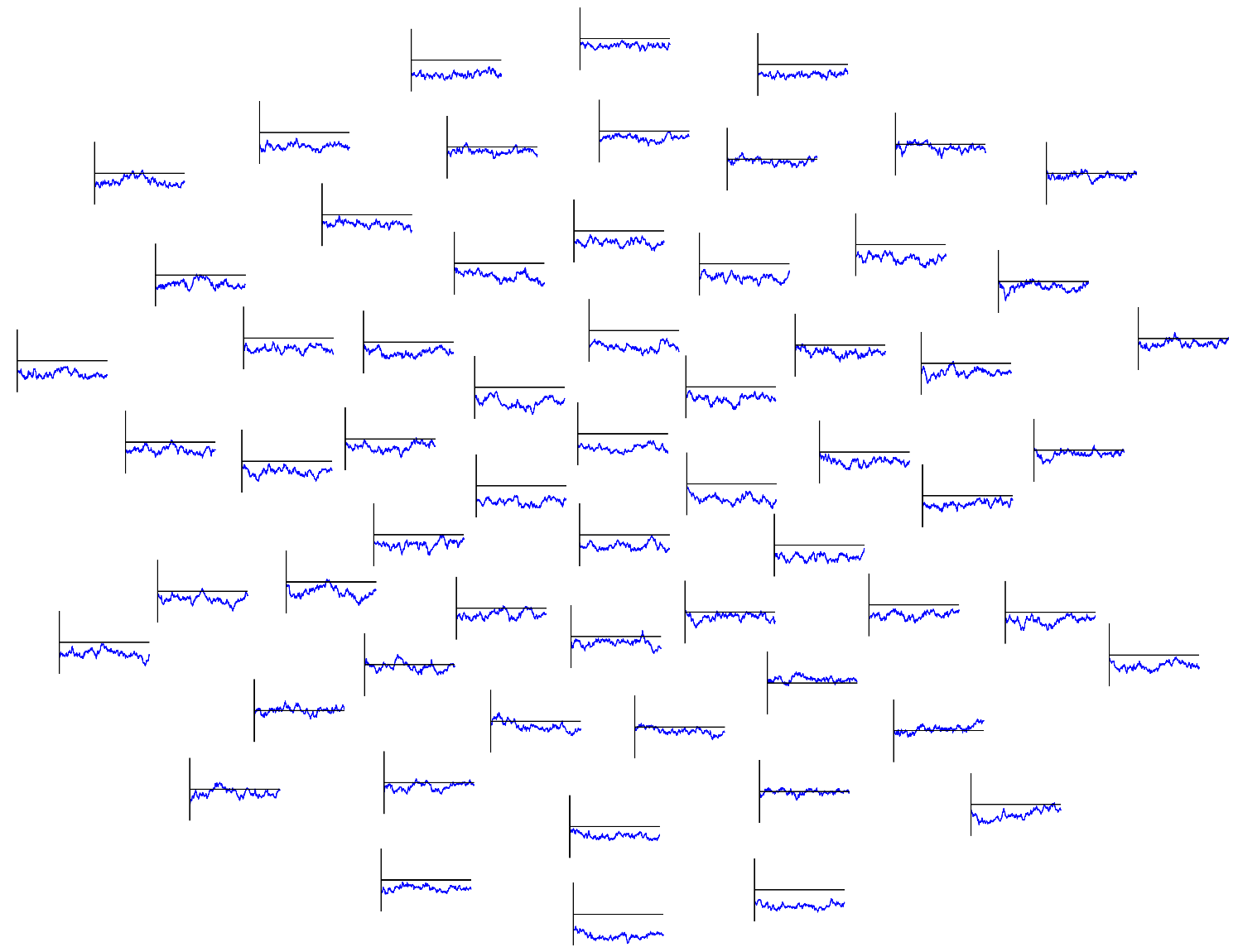 |
| --- |
| *Supplementary Figure 24*. In the autistic group and in the 70 dB condition, waveforms at each electrode depicting Spearman’s ρ ordinal correlation coefficients between loudness discomfort and median absolute deviations of EEG amplitudes across trials between 1 ms (left of each channel subplot) and 350 ms (right of each channel subplot). The Y-axis, representing correlation coefficients, ranges from −0.325 (bottom of each channel subplot) to +0.325 (top of each channel subplot). There were no significant correlations. |

| 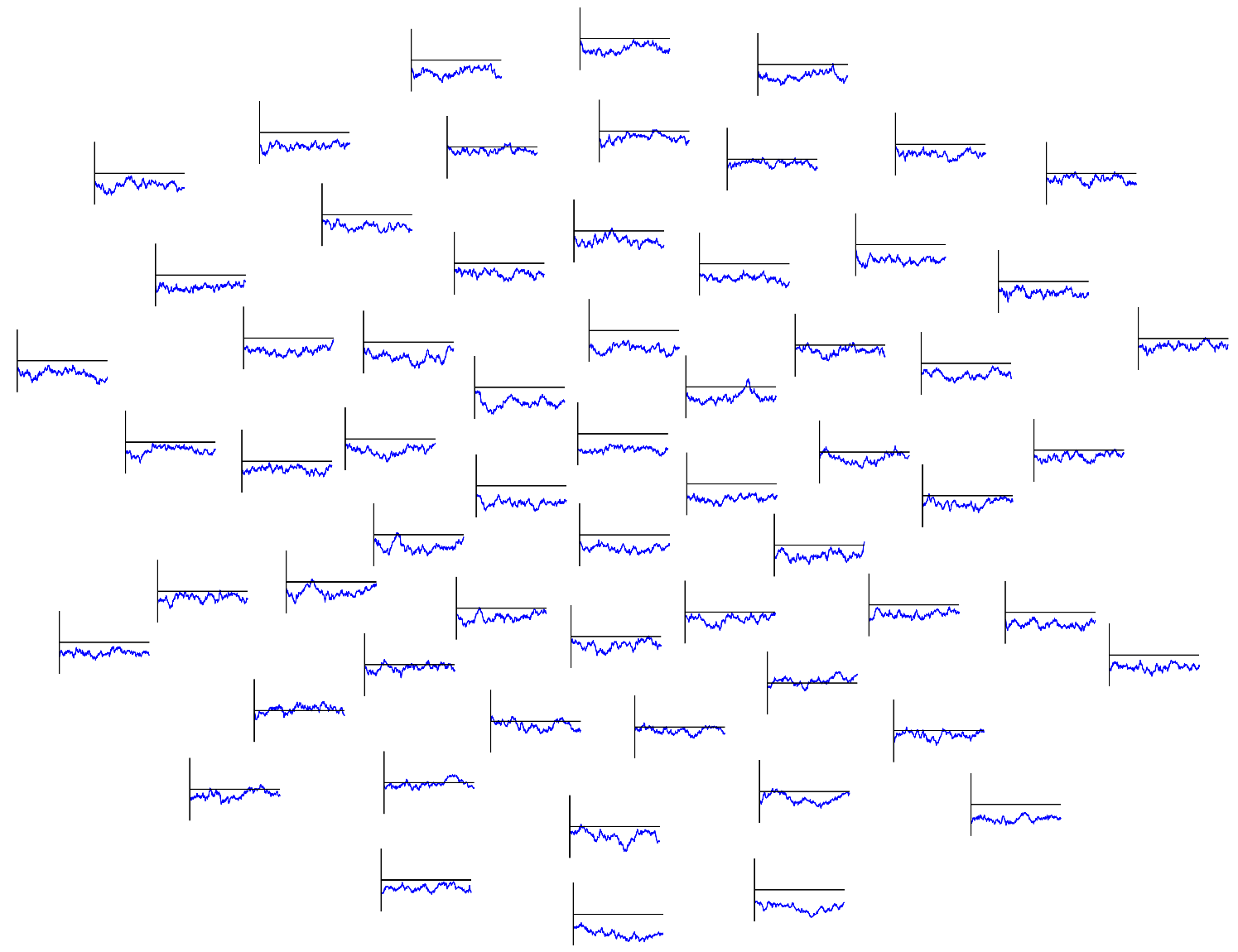 |
| --- |
| *Supplementary Figure 25*. In the autistic group and in the 80 dB condition, waveforms at each electrode depicting Spearman’s ρ ordinal correlation coefficients between loudness discomfort and median absolute deviations of EEG amplitudes across trials between 1 ms (left of each channel subplot) and 350 ms (right of each channel subplot). The Y-axis, representing correlation coefficients, ranges from −0.325 (bottom of each channel subplot) to +0.325 (top of each channel subplot). There were no significant correlations. |

# Appendix F. Supplementary ITPC Figures

| 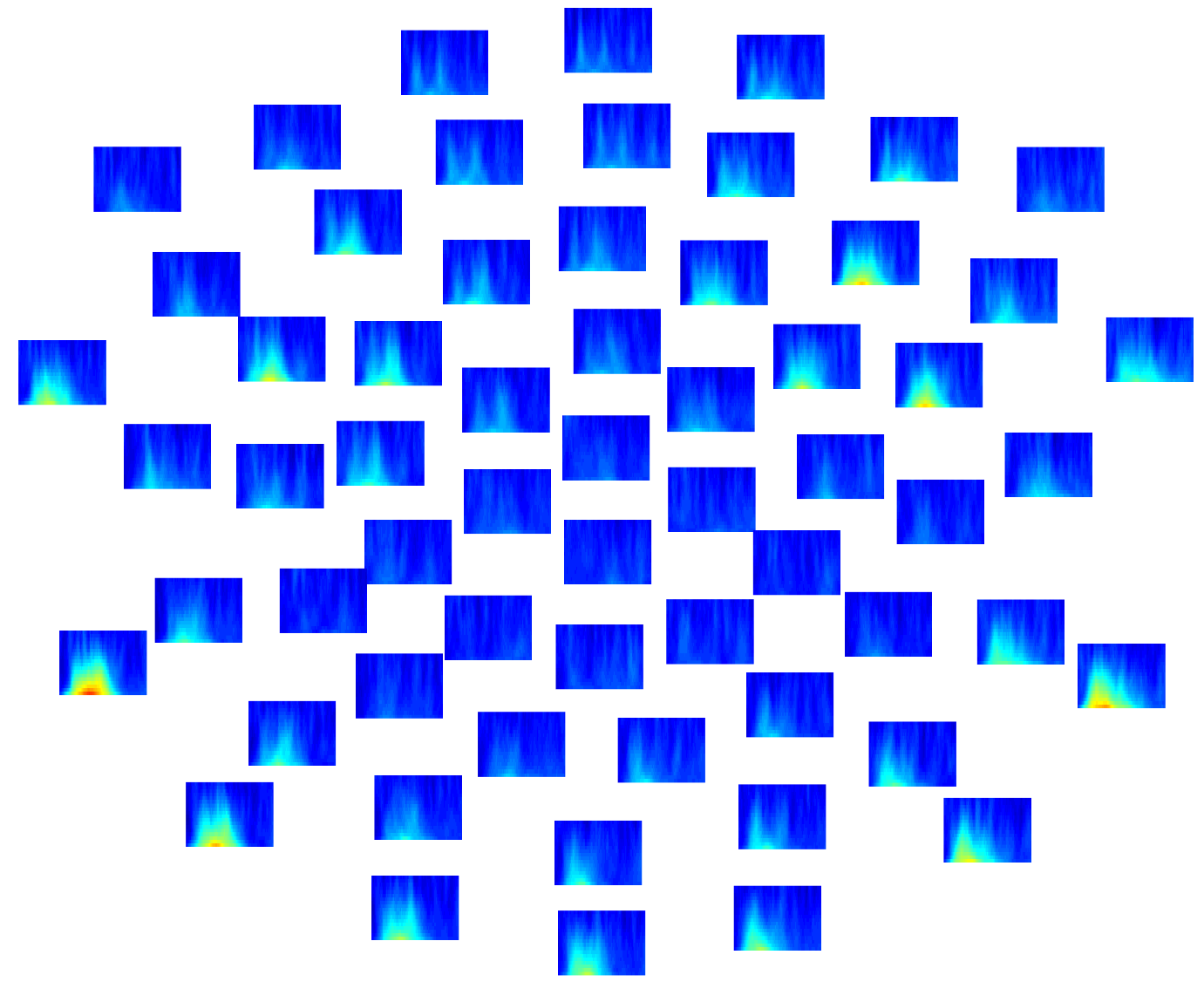 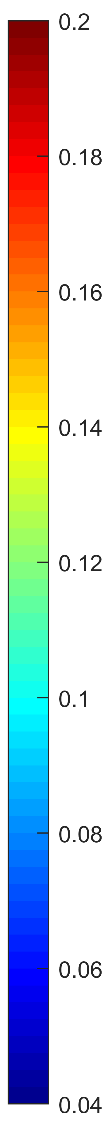 |
| --- |
| *Supplementary Figure 26*. Mean ITPC in the typically-developing group in the 50 dB condition at each channel at time points between 1 – 350 ms. A significant difference was observed in the 50 dB condition such that there was greater ITPC in the ASD group than the TD group, *p* = .05. Group differences are displayed in *Figure 4* from the main text. |

| 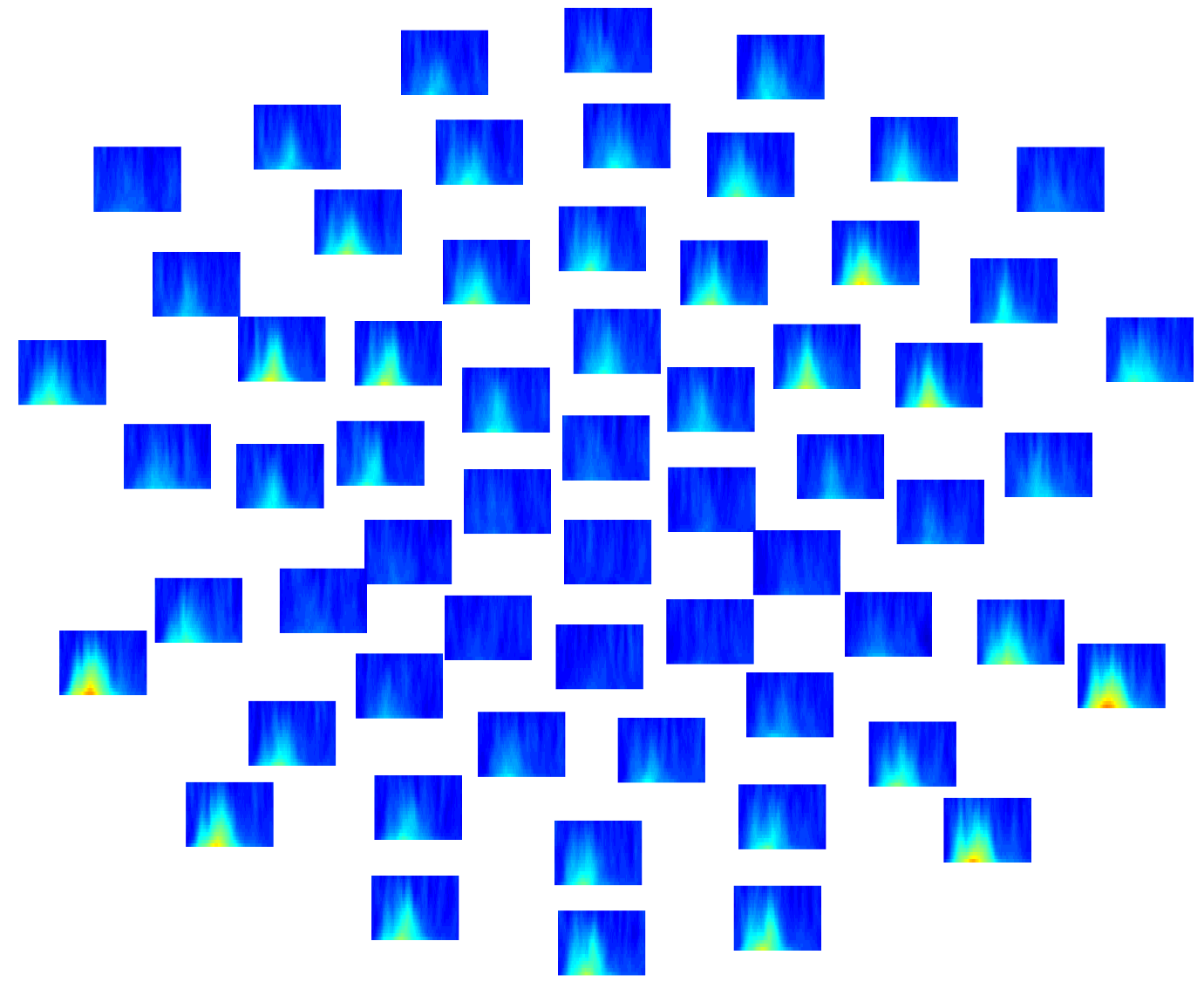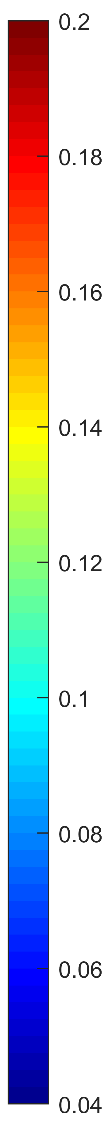 |
| --- |
| *Supplementary Figure 27*. Mean ITPC in the autistic group in the 50 dB condition at each channel at time points between 1 – 350 ms. A significant difference was observed in the 50 dB condition such that there was greater ITPC in the ASD group than the TD group, *p* = .05. Group differences are displayed in *Figure 4* from the main text. |

| 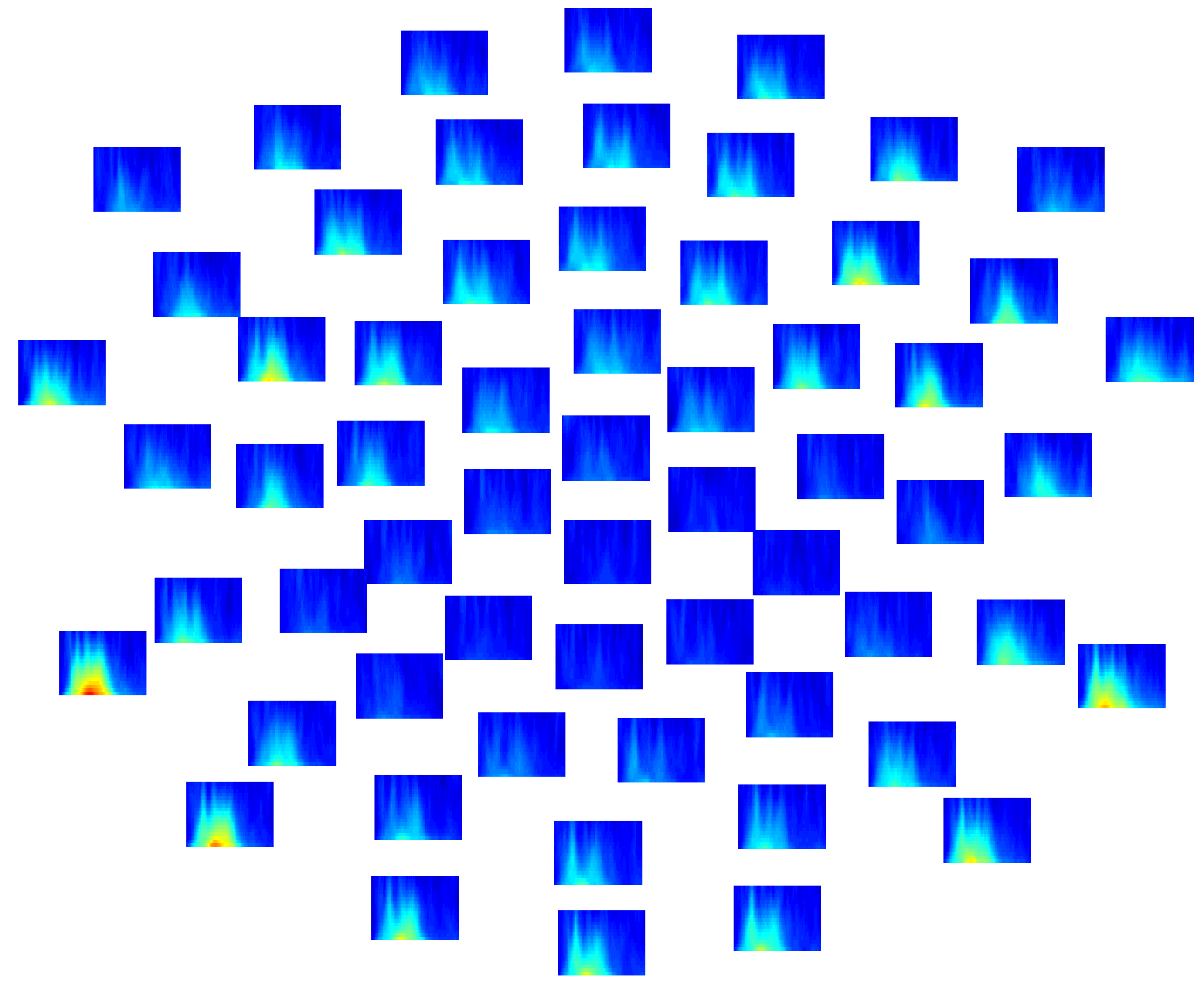 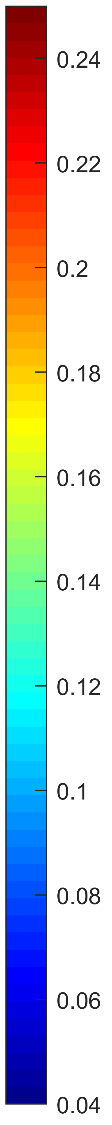 |
| --- |
| *Supplementary Figure 28*. Mean ITPC in the typically-developing group in the 60 dB condition at each channel at time points between 1 – 350 ms. A significant difference was observed in the 60 dB condition such that there was greater ITPC in the ASD group than the TD group, *p* = .03. Group differences are displayed in *Figure 5* from the main text. |

| 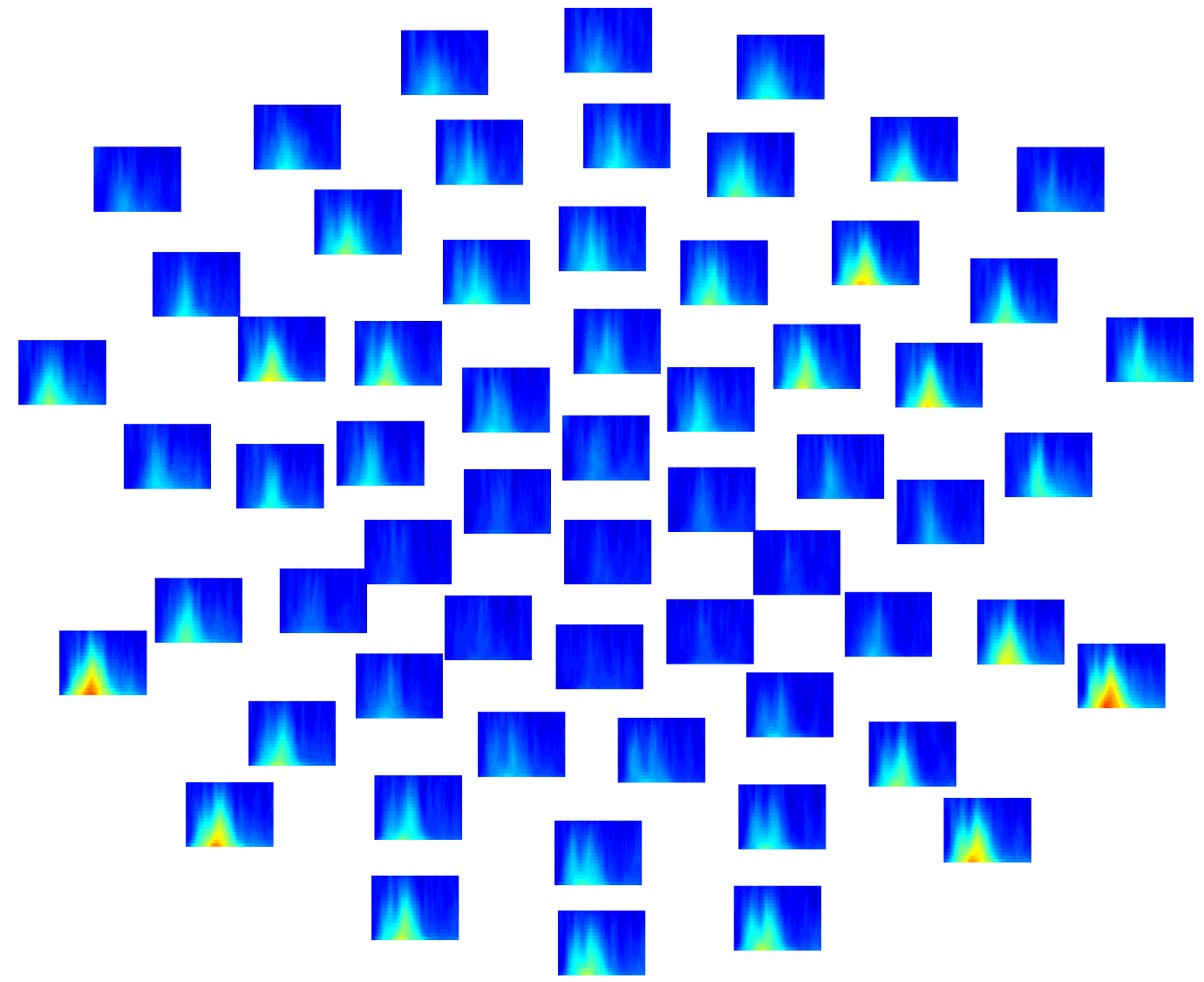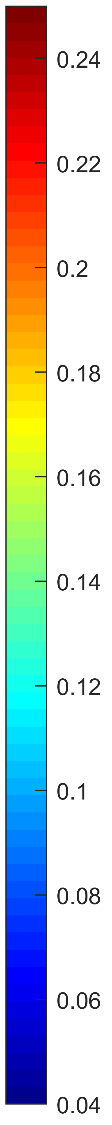 |
| --- |
| *Supplementary Figure 29*. Mean ITPC in the autistic group in the 60 dB condition at each channel at time points between 1 – 350 ms. A significant difference was observed in the 60 dB condition such that there was greater ITPC in the ASD group than the TD group, *p* = .03. Group differences are displayed in *Figure 5* from the main text. |

| 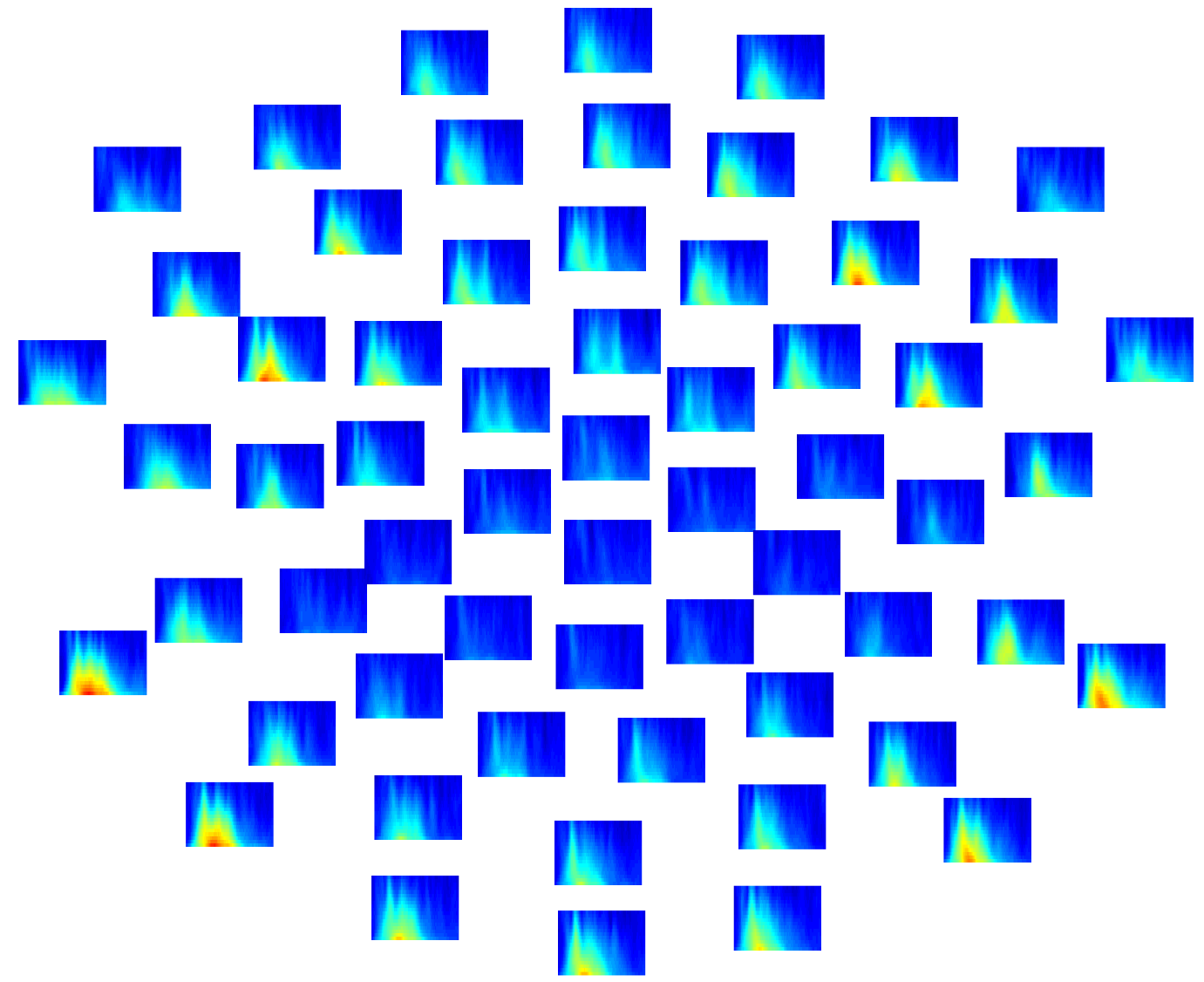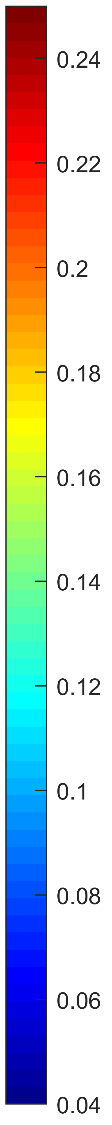 |
| --- |
| *Supplementary Figure 30*. Mean ITPC in the typically-developing group in the 70 dB condition at each channel at time points between 1 – 350 ms. No significant differences were observed between TD and ASD in the 70 dB condition. |

| 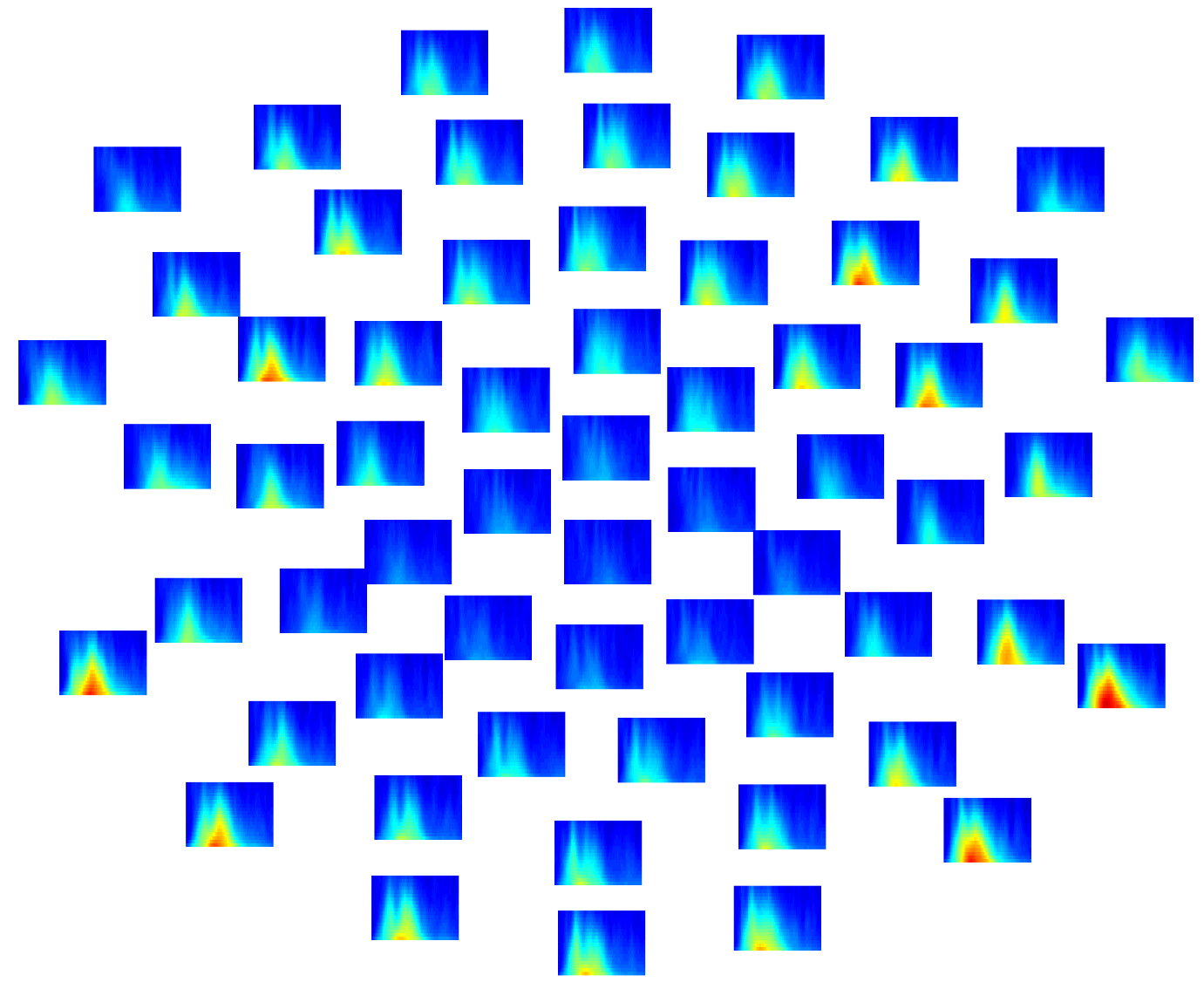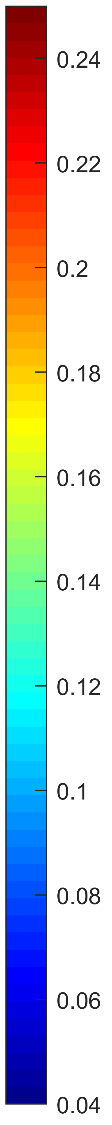 |
| --- |
| *Supplementary Figure 31*. Mean ITPC in the autistic group in the 70 dB condition at each channel at time points between 1 – 350 ms. No significant differences were observed between TD and ASD in the 70 dB condition. |

| 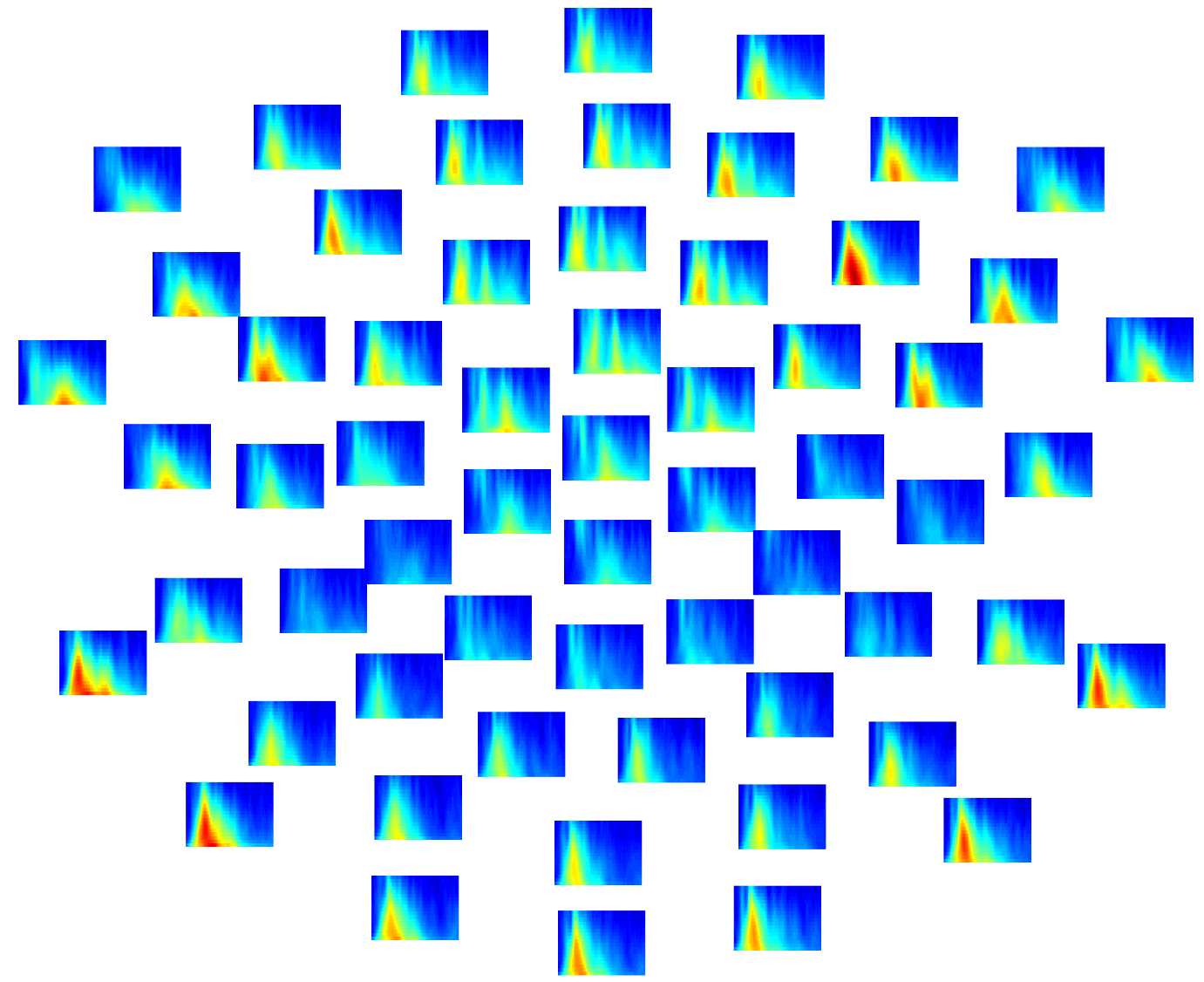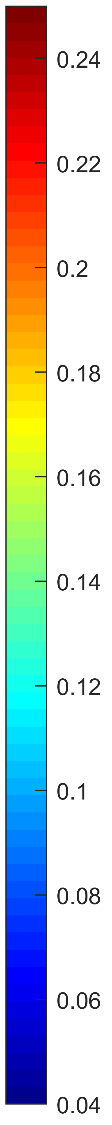 |
| --- |
| *Supplementary Figure 32*. Mean ITPC in the typically-developing group in the 80 dB condition at each channel at time points between 1 – 350 ms. No significant differences were observed between TD and ASD in the 80 dB condition. |

# Appendix G. Correlations of ITPC and Loudness Discomfort Items in Autism, 50 dB Condition

As noted in the main text, in autistic participants in the 50 dB condition, we observed a significant and unexpected correlation between SPHI loudness discomfort and ITPC, such that participants with less consistency of phase across trials exhibited more loudness discomfort. To aid in interpretation of this effect, we conducted unplanned, *post hoc* supplementary analyses exploring ordinal correlations, in autistic participants in the 50 dB condition, between ITPC and scores on each of the five Short Sensory Profile (SSP) items that are included in the SPHI.

As in the main text, we used cluster-based permutation tests (Maris & Oostenveld, 2007) to explore Spearman’s ρ (rho) correlations between scores on each item and ITPC in all channels, all frequencies between 6 and 40 Hz, and all time points between 1 and 350 ms.

SSP items are reverse-scored relative to the SPHI, with lower scores indicating greater loudness discomfort. Thus, negative correlations in the main text correspond to positive correlations below.

Results are presented in Supplementary Table 1. Only one correlation initially attained significance, involving the item, “Holds hands over ears to protect ears from sound”; however, this effect no longer reached statistical significance when a Benjamini-Hochberg correction for five comparisons was applied. The direction and topography of this effect was comparable to the SPHI correlation from the main text (*Supplementary Figure 33*).

| Supplementary Table 1. Lowest permutation test *p*-values reflecting correlations between ITPC and SSP items included in SPHI in autistic participants, 50 dB condition, presented both uncorrected and with a Benjamini-Hochberg correction for five comparisons. | | | | |
| --- | --- | --- | --- | --- |
| Item # | Text | Lowest *p* | | Direction |
|  |  | Raw | Corrected |  |
| 22 | Is distracted and has trouble functioning if there is a lot of noise around | .09 | .21 | Positive |
| 24 | Can’t work with background noise (for example, fan, refrigerator) | .26 | .32 | Positive |
| 25 | Has trouble completing tasks when radio is on | >.99 | >.99 | Positive |
| 34 | Responds negatively to unexpected or loud noises (for example, cries or hides at noise from vacuum cleaner, dog barking, hair dryer) | .13 | .22 | Negative |
| 35 | Holds hands over ears to protect ears from sound | .03 | .13 | Positive |

| 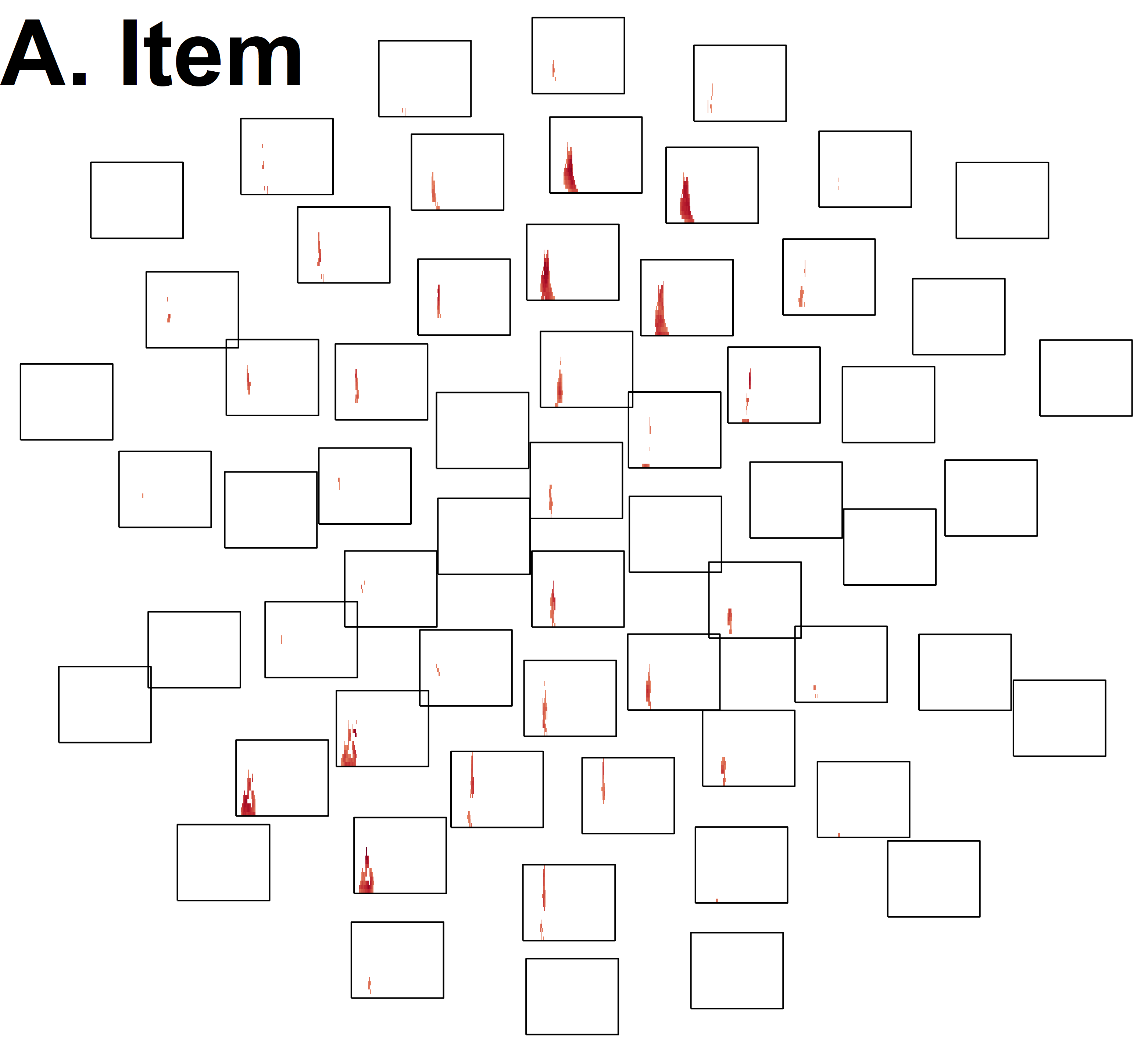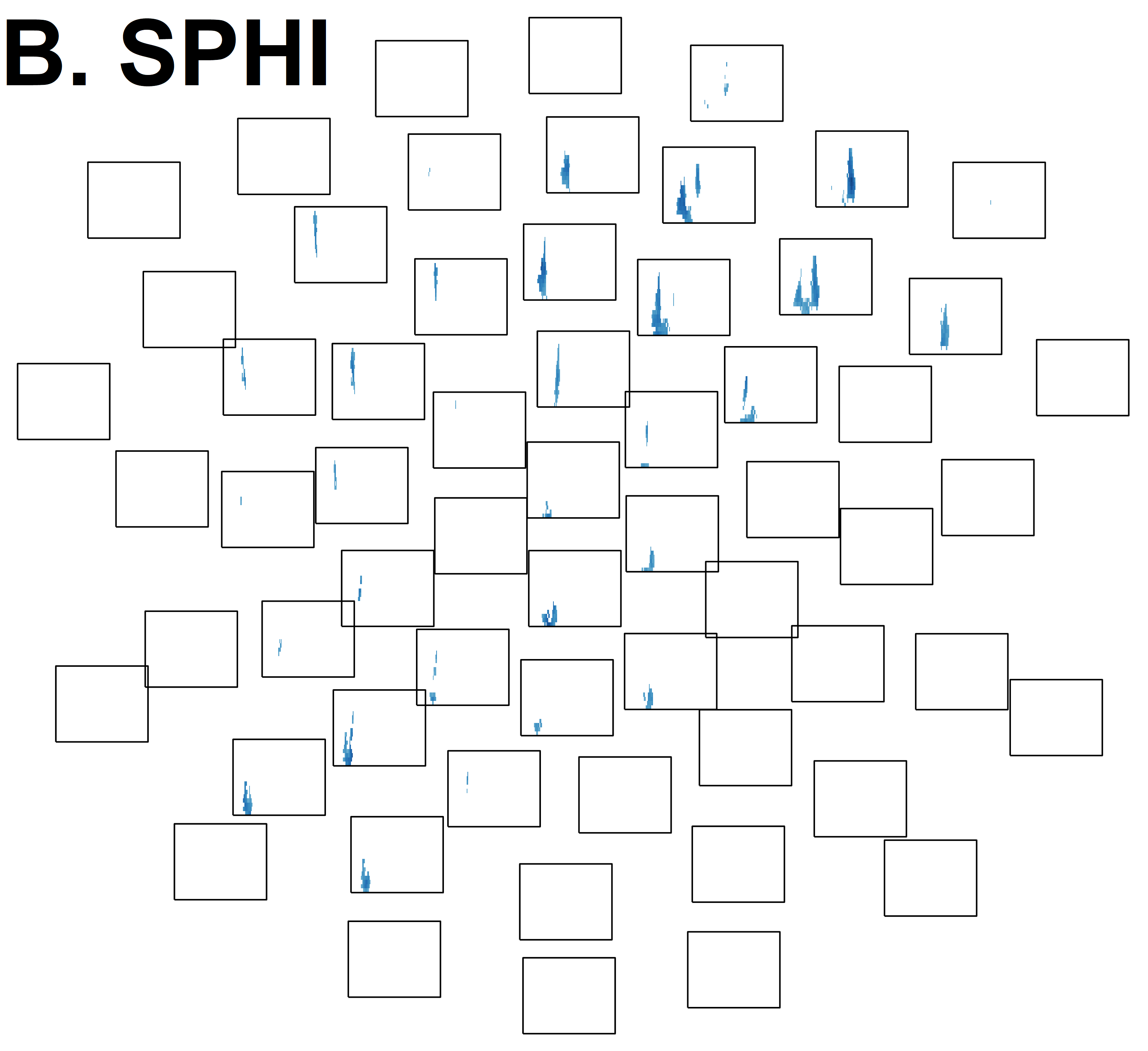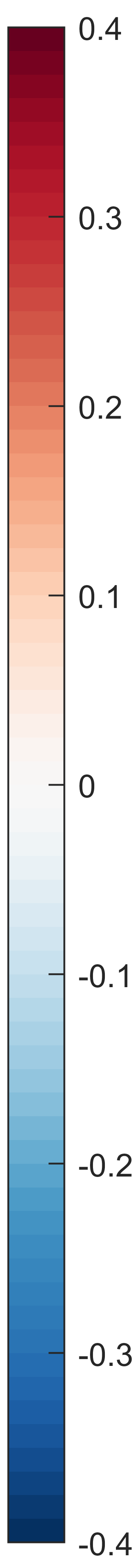 |
| --- |
| *Supplementary Figure 33*. In the autistic group, spectral plots at each electrode depicting, in the 50 dB condition, Spearman’s ρ ordinal correlation coefficients between loudness discomfort and ITPC of EEG responses in each frequency (40 Hz at top; 6 Hz at bottom of each subplot ) between 1 ms (left of each channel subplot) and 350 ms (right of each channel subplot). Only significant clusters are shown.  A (top left). The negative correlation between ITPC and scores on item 35, “Holds hands over ears to protect ears from sound.” Note that this correlation cluster effect did not achieve statistical significance after correction for the multiple comparisons involved in examining multiple items.  B (top right). The positive correlation between ITPC and overall SPHI estimates. Note that the scoring of the SPHI is reversed relative to the SSP items that comprise it, so the correlation effects are equivalent in terms of directionality. |

# Supplementary References

Bürkner, P. C. (2017). brms: An R package for Bayesian multilevel models using Stan. *Journal of Statistical Software, 80*(1), 1–28. https://doi.org/10.18637/jss.v080.i01

Kirk, R. E. (1996). Practical significance: A concept whose time has come. *Educational and Psychological Measurement, 56*(5), 746–759. https://doi.org/10.1177/0013164496056 005002

Maris, E., & Oostenveld, R. (2007). Nonparametric statistical testing of EEG- and MEG-data. *Journal of Neuroscience Methods, 164*(1), 177–190. https://doi.org/10.1016/j.jneumeth.2007.03.024

Ponton, C., Eggermont, J., Khosla, D., Kwong, B., & Don, M. (2002). Maturation of human central auditory system activity: Separating auditory evoked potentials by dipole source modeling. Clinical *Neurophysiology, 113*, 407–420. https://doi.org/10.1016/S1388-2457(01)00733-7

Wagenmakers, E.-J., Wetzels, R., Borsboom, D., & van der Maas, H. L. J. (2011). Why psychologists must change the way they analyze their data: The case of psi: Comment on Bem (2011). *Journal of Personality and Social Psychology, 100*(3),426–432. https://doi.org/10.1037/a0022790
